# Eremophilane-Type Sesquiterpenoids From the Endophytic Fungus *Rhizopycnis vagum* and Their Antibacterial, Cytotoxic, and Phytotoxic Activities

**DOI:** 10.3389/fchem.2020.596889

**Published:** 2020-10-26

**Authors:** Ali Wang, Ruya Yin, Zhiyao Zhou, Gan Gu, Jungui Dai, Daowan Lai, Ligang Zhou

**Affiliations:** ^1^Department of Plant Pathology, College of Plant Protection, China Agricultural University, Beijing, China; ^2^State Key Laboratory of Bioactive Substance and Function of Natural Medicines, Institute of Materia Medica, Chinese Academy of Medical Science, Peking Union Medical College, Beijing, China

**Keywords:** *Rhizopycnis vagum*, eremophilanes, sesquiterpenoids, rhizoperemophilanes, structure elucidation, biological activities

## Abstract

Fourteen new eremophilane-type sesquiterpenoids, named rhizoperemophilanes A~N (**1**~**14**), together with eight known congeners, were isolated from the culture of the endophytic fungus *Rhizopycnis vagum*. The structures of the new compounds were elucidated by extensive spectroscopic analyses, as well as ECD calculations and the modified Mosher's method for the assignment of the absolute configurations. Rhizoperemophilane J (**10**) contains an uncommon C-4/C-11 epoxy ring, while rhizoperemophilane N (**14**) features an unprecedented 3-nor-eremophilane lactone-lactam skeleton. These metabolites were evaluated for their antibacterial, cytotoxic, and phytotoxic activities. Among them, compounds **11**, **16**, and **20** displayed antibacterial activities, while **14** showed selective cytotoxicity against NCI-H1650 and BGC823 tumor cells. Moreover, compounds **5**, **6**, **12**, **13**, **16**, and **19** exhibited strong phytotoxic activities against the radicle elongation of rice seedlings.

## Introduction

Eremophilanes are one type of sesquiterpenoids found in fungi and plants of various origins (Hou et al., 2014; Yuyama et al., 2017). So far, around 180 members were reported from fungi (Yuyama et al., 2017; Niu et al., 2018), which displayed a wide range of bioactivities including antimicrobial, cytotoxic, and anti-inflammatory properties. For examples, berkleasmins A and C showed potent cytotoxicities (Isaka et al., 2009); penicilleremophilane A and sporogen AO-1 displayed significant antimalarial activities against *Plasmodium falciparum* (Daengrot et al., 2015); periconianones A and B exhibited strong anti-inflammatory activities (Zhang et al., 2014). In recent years, a growing number of bioactive eremophilanes have been isolated from endophytic fungi (Liu et al., 2015, 2016, 2017).

*Rhizopycnis vagum* was an endophytic fungus previously isolated from *Nicotiana tabacum*. This fungus was capable to produce a plethora of dibenzo-α-pyrone derivatives and two anisic acid derivatives (Lai et al., 2016; Wang et al., 2018, 2020). In our continuing interests in searching for new bioactive substances, we carried out a further chemical investigation of this fungus. This led to the isolation of twenty-two eremophilane-type sesquiterpenoids, including fourteen new compounds (**1**~**14**). Such type of metabolites has not been reported from the genus *Rhizopycnis* previously. Herein, we report the isolation, structure elucidation, and bioactivities of these metabolites.

## Materials and Methods

### General Experimental Procedures

UV spectra were recorded on a TU-1810 UV–vis spectrophotometer (Beijing Persee General Instrument Co., Ltd., Beijing, China). Specific rotations were recorded on a Rudolph Autopol IV automatic polarimeter (Rudolph Research Analytical, NJ, USA). Circular dichroism (CD) spectra were recorded on a JASCO J-810 CD spectrometer (JASCO Corp., Tokyo, Japan). Infrared (IR) spectra were measured on a Thermo Nicolet Nexus 470 FT-IR spectrometer (Thermo Electron Scientific Instrument Crop., WI, USA). High-resolution electrospray ionization mass spectrometry (HRESIMS) spectra were recorded on an LC 1260-Q-TOF/MS 6520 machine (Agilent Technologies, CA, USA). Mass condition was as followed. ESI source: gas Temp 350°C, drying gas 12 l/min, nebulizer 40 psig, VCap 4000 V (+)/3500 V (–); MS TOF: fragmentor 140 V, Skimmer 65 V, OCT 1 RF Vpp 750 V; TOF spectra: mass range 50–1100 *m/z*, acquisition rate 2 spectra/s. ^1^H, ^13^C, and 2D NMR spectra were measured on an Avance 400 NMR spectrometer (Bruker BioSpin, Zurich, Switzerland). Chemical shifts are expressed in δ (ppm), and coupling constants (*J*) in hertz. The ^1^H NMR (δ_H_) data were referenced to the inner standard tetramethylsilane, while the ^13^C NMR (δ_C_) data were referenced to the solvent residual peaks at 77.0 (CDCl_3_), 49.0 (CD_3_OD), and 39.5 (DMSO-*d*_6_), respectively. Silica gel (200~300 mesh, Qingdao Marine Chemical Inc., Qingdao, China) and Sephadex LH-20 (Pharmacia Biotech, Uppsala, Sweden) were used for column chromatography. Medium-pressure liquid chromatography (MPLC) separation was carried out on an Eyela-VSP-3050 instrument (Tokyo Rikakikai Co., Tokyo, Japan). HPLC-DAD analysis was performed using a Shimadzu LC-20A instrument with an SPD-M20A photodiode array detector (Shimadzu Corp., Tokyo, Japan) and an analytic C_18_ column (250 mm × 4.6 mm i.d., 5 μm; Phenomenex Inc., Torrance, CA, USA). Semi-preparative HPLC separation was carried out on a Lumtech instrument (Lumiere Tech. Ltd., Beijing, China) equipped with a K-501 pump (flow rate: 3 mL/min) and a K-2501 UV detector using a Luna-C_18_ column (250 mm × 10 mm i.d., 5 μm, Phenomenex Inc.). Precoated silica gel GF-254 plates (Qingdao Marine Chemical Inc.) were used for analytical TLC. Spots were visualized under UV light (254 nm or 356 nm) or by spraying with 10% H_2_SO_4_ in 95% EtOH followed by heating.

### Fungal Source, Fermentation, and Extraction

The endophytic fungus *Rhizopycnis vagum* (strain: Nitaf22, GenBank accession no. KM095527) was isolated previously (Lai et al., 2016) and deposited in our lab. The fungus was cultured on potato dextrose agar for 5 days at 25°C, before inoculating the potato dextrose broth (150 mL) in a 250-mL flask. The culture was shaken for 7 days at 150 rpm and 25°C, which was then used as inoculum to inoculate the autoclaved rice media in 1-L Erlenmeyer flasks each containing 100 g of rice and 110 mL of distilled water. The fermentation was carried out on a total of 10 kg of rice under static conditions at room temperature for 50 days. The cultures were then extracted with EtOAc for three times, which afforded 163 g of crude extract.

### Isolation of Secondary Metabolites

The extract was subjected to vacuum liquid chromatography (VLC) over silica gel (i.d. 8 × 30 cm) by eluting with a different mixture of petroleum ether (PE) and acetone, then CH_2_Cl_2_-MeOH. Fractions were pooled according to TLC, and five fractions were obtained (Frs. A~E).

Fr. A (35.4 g) was again subjected to VLC over silica gel by eluting with a gradient of PE/acetone (100:0→50:50, v/v) to give eight subfractions (Frs. A1~A8). Among them, Fr. A5 and Fr. A6 were found to contain sesquiterpenoids. Then, Fr. A5 was fractionated by medium-pressure liquid chromatography (MPLC) over silica gel employing the same solvent system as that of Fr. A, to yield five further fractions (Frs. A5-1~A5-5). Fr. A5-2 was chromatographed over Sephadex LH-20 using PE/CH_2_Cl_2_/MeOH (5:5:1, v/v) as the mobile phase (note: this solvent system was used for all the chromatography over Sephadex LH-20 in the following process) to obtain four fractions, which were purified by semi-preparative HPLC to yield **10** (5.9 mg) from Fr. A5-2-1 (38% MeOH/H_2_O as the mobile phase), **9** (1.0 mg) from Fr. A5-2-2 (40% MeOH/H_2_O), **6** (12.0 mg) from Fr. A5-2-3 (48% MeOH/H_2_O), and **5** (5.0 mg) from Fr. A5-2-4 (48% MeOH/H_2_O). Likewise, Fr. A6 was processed in a similar manner, by MPLC eluting with PE/acetone (50:1→50:50) to obtain Frs. A6-1~A6-5. These fractions were subjected to column chromatography (CC) over Sephadex LH-20 prior to purification by semi-preparative HPLC. Compounds **16** (9.7 mg) and **17** (1.8 mg) were obtained from Fr. A6-1 (65% MeOH/H_2_O) and Fr. A6-2 (55% MeOH/H_2_O), respectively, while **20** (2.5 mg) and **12** (1.7 mg) were purified from Fr. A6-3 (50% MeOH/H_2_O).

Fr. B (26.6 g) was subjected to MPLC over silica gel by eluting with a gradient of CH_2_Cl_2_/MeOH (100:0→50:50, v/v) to give seven subfractions (Frs. B1~B7). Fr. B1 was chromatographed over ODS using a gradient of MeOH/H_2_O (35%→100%) as the mobile phase to give nine subfractions Frs. B1-1~B1-9. Compound **19** (15.0 mg) was obtained from Fr. B1-1 through repeated crystallization, while **21** (2.8 mg) was purified from Fr. B1-6 by semi-preparative HPLC (63% MeOH/H_2_O). Fr. B2 was subjected to MPLC over silica gel eluting with PE/acetone (50:0→50:50, v/v) to give six subfractions (Frs. B2-1~B2-6), which were then chromatographed over Sephadex LH-20 before a final purification by semi-preparative HPLC (50% MeOH/H_2_O). This led to the isolation of **11** (1.6 mg) from Fr. B2-2, and **13** (7.5 mg) from Fr. B2-3. Similarly, Fr. B4 was subjected to CC over Sephadex LH-20 to yield five subfractions (Frs. B4-1~B4-5). Compound **22** (3.3 mg) was purified from Fr. B4-1 by semi-preparative HPLC (56% MeOH/H_2_O), while **2** (3.3 mg) and **18** (2.0 mg) were obtained from Fr. B4-2 using the same process. Likewise, Fr. B5 was fractionated by the same way as that of Fr. B4 to give four subfractions (Frs. B5-1~B5-4). **15** (3.7 mg) and **14** (4.3 mg) were purified from Fr. B5-2 by semi-preparative HPLC (42% MeOH/H_2_O).

Fr. C (7.7 g) was processed in the same way as that of Fr. B to afford seven fractions (Frs. C1~C7). Among them, Fr. C5 was subjected to CC over Sephadex LH-20, before purification by semi-preparative HPLC (52% MeOH/H_2_O) to yield **1** (7.0 mg). Fr. C7 was processed likewise to obtain six fractions (Frs. C7-1~C7-6) after CC over Sephadex LH-20. These were further purified by semi-preparative HPLC to afford **7** (2.0 mg), **8** (8.0 mg), and **4** (2.6 mg), from Fr. C7-2 (eluting with 43% MeOH/H_2_O), Fr. C7-4 (45% MeOH/H_2_O), and Fr. C7-5 (40% MeOH/H_2_O), respectively.

Fr. D (6.1 g) was fractionated in a similar manner as that of Fr. B to obtain eight subfractions (Frs. D1~D8). Fr. D4 was subjected to CC over Sephadex LH-20 and purified by semi-preparative HPLC eluting with 45% MeOH/H_2_O to yield **3** (2.3 mg).

#### Rhizoperemophilane A (1)

Colorless oil; ${[\alpha ]}_{\text{D}}^{26}$ +74.0 (*c* 0.1, CHCl_3_); UV (MeOH) λ_max_ (log ε) 206 (3.52), 250 (3.59), 280 (3.40) nm; ECD (*c* = 2.0 ×10^−3^ M, MeOH) λ (Δε) 220 (−0.29), 248 (+9.40), 284 (−2.93) nm; IR (KBr) ν_max_ 3403, 2963, 2918, 2878, 1656, 1624, 1453, 1371, 1296, 1262, 1227, 1125, 1070, 1022, 979, 931, 910, 896, 801, 728, 616 cm^−1^; ^1^H NMR, and ^13^C NMR, see Tables 1, 2; HRESIMS *m*/*z* 251.1640 [M+H]^+^ (Calcd. for C_15_H_23_O_3_, 251.1642), 273.1463 [M+Na]^+^ (Calcd. for C_15_H_22_O_3_Na, 273.1461).

**Table 1:** ^13^C NMR (100 MHz) data of compounds **1**~**14**.

**Position**	**1^a^**	**2^a^**	**3^a^**	**4^a^**	**5^a^**	**6^a^**	**7^a^**	**8^a^**	**9^a^**	**10^a^**	**11^a^**	**12^a^**	**13^c^**	**14^a^**	**14^b^**
1	65.2, CH	76.4, CH	78.7, CH	129.06, CH*^d^*	74.3, CH	32.6, CH_2_	128.5, CH	65.5, CH	32.6, CH_2_	58.1, CH	146.4, C	127.8, CH	124.7, CH	114.7, CH	113.5, CH
2	43.8, CH_2_	72.3, CH	72.1, CH	136.1, CH	35.1, CH_2_	41.8, CH_2_	135.9, CH	44.5, CH_2_	40.6, CH_2_	61.3, CH	176.3, C	181.4, C	192.6, C	167.0, C	163.5, C
3	72.3, CH	34.3, CH_2_	77.6, CH	74.3, CH	26.2, CH_2_	211.4, C	74.2, CH	72.0, CH	212.1, C	73.7, CH	145.5, C	146.7, C	72.8, CH	-	-
4	47.1, CH	41.1, CH	45.0, CH	75.0, C	48.7, CH	57.4, CH	76.1, C	46.7, CH	54.3, CH	82.0, C	135.9, C	136.0, C	42.3, CH	106.8, C	104.9, C
5	43.5, C	41.7, C	40.8, C	44.6, C	41.6, C	43.6, C	43.5, C	41.4, C	43.2, C	44.5, C	41.7, C	46.9, C	38.8, C	43.7, C	41.6, C
6	43.7, CH_2_	43.4, CH_2_	44.7, CH_2_	35.9, CH_2_	48.8, CH_2_	44.2, CH_2_	39.5, CH_2_	42.8, CH_2_	40.7, CH_2_	39.1, CH_2_	33.0, CH_2_	32.9, CH_2_	33.8, CH_2_	29.9, CH_2_	28.2, CH_2_
7	128.9, C	129.3, C	128.9, C	129.7, C	78.9, C	78.2, C	78.6, C	77.3, C	76.7, C	77.3, C	147.5, C	142.4, C	139.0, C	139.9, C	138.1, C
8	194.4, C	194.4, C	194.0, C	194.1, C	205.2, C	203.3, C	203.6, C	202.8, C	201.5, C	200.0, C	151.2, C	148.0, C	142.2, C	144.7, C	142.9, C
9	122.6, CH	130.7, CH	130.7, CH	129.10, CH^d^	123.5, CH	123.2, CH	124.8, CH	118.5, CH	124.3, CH	126.5, CH	103.6, C	174.8, C	107.4, C	106.0, CH	103.8, CH
10	174.0, C	168.1, C	167.2, C	162.1, C	169.3, C	166.8, C	162.2, C	175.6, C	167.9, C	168.7, C	131.8, C	161.3, C	163.9, C	159.8, C	157.1, C
11	145.2, C	146.0, C	147.2, C	146.8, C	33.5, CH	33.6, CH	33.6, CH	35.1, CH	34.8, CH	77.3, C	125.1, C	124.2, C	129.2, C	129.7, C	127.7, C
12	22.8, CH_3_	22.9, CH_3_	23.0, CH_3_	23.2, CH_3_	15.9, CH_3_	16.4, CH_3_	16.5, CH_3_	16.2, CH_3_	16.1, CH_3_	27.3, CH_3_	172.3, C	149.5, CH	173.1, C	174.9, C	172.2, C
13	22.3, CH_3_	22.5, CH_3_	22.7, CH_3_	22.8, CH_3_	17.0, CH_3_	16.8, CH_3_	16.9, CH_3_	17.8, CH_3_	18.1, CH_3_	26.6, CH_3_	8.5, CH_3_	7.5, CH_3_	8.5, CH_3_	8.2, CH_3_	8.1, CH_3_
14	20.2, CH_3_	18.6, CH_3_	21.5, CH_3_	22.1, CH_3_	19.8, CH_3_	20.6, CH_3_	23.8, CH_3_	25.8, CH_3_	23.1, CH_3_	22.4, CH_3_	29.1, CH_3_	25.9, CH_3_	23.3, CH_3_	24.6, CH_3_	23.9, CH_3_
15	12.3, CH_3_	15.4, CH_3_	12.3, CH_3_	19.8, CH_3_	15.1, CH_3_	7.5, CH_3_	19.8, CH_3_	12.5, CH_3_	8.0, CH_3_	21.1, CH_3_	11.3, CH_3_	11.2, CH_3_	10.3, CH_3_	22.3, CH_3_	21.9, CH_3_
-OAc													169.9, C,		
													20.8, CH_3_		

Recorded in

a *CD_3_OD*;

b *DMSO-d_6_*;

c *CDCl_3_*.

d *assignments within a column might be interchanged*.

**Table 2:** ^1^H NMR (400 MHz) Data of **1**~**7** in CD_3_OD [δ_H_, mult. (*J* in Hz)].

**Position**	**1**	**2**	**3**	**4**	**5**	**6**	**7**
1	4.63, ddd (12.6, 5.2, 1.4)	4.18, d (3.1)	4.31 dd (3.5, 1.5)	6.27, d (9.8)	4.29, br.s	2.81, td (13.7, 6.7) 2.70, ov.*^a^*	6.30, d (9.9)
2	2.28, ddd (12.7, 5.2, 3.3) 1.65, td (12.7, 2.8)	3.62, dt (12.0, 3.8)	3.60, dd (3.5, 3.2)	6.17, dd (9.8, 5.0)	1.96, dd (14.0, 3.2) 1.67, ddd (14.0, 4.2, 3.4)	2.60, td (13.6, 7.2) 2.50, dd (13.9, 6.7)	6.10, dd (9.9, 4.7)
3	3.95, ddd (3.3, 2.9, 2.8)	1.87, ov.*^a^* 1.56, m	3.87 ddd (3.2, 2.6, 1.5)	3.93, d (5.0)	1.85, td (12.9, 3.5) 1.35, m		3.86, d (4.7)
4	1.54, qd (7.1, 2.9)	1.54, m	1.59, qd (7.1, 2.6)		1.43, m	2.71, ov.*^a^*	
6	2.96, d (13.6) 2.14, br, d (13.7)	2.98, d (13.9) 2.09, br. d/ov.*^a^*	3.02, d (13.8) 2.12, br. d/ov.*^a^*	2.80, s	2.33, d (14.1) 1.64, d (14.2)	2.23, d (14.5) 1.88, d (14.5)	2.36, d (14.1), 2.16, d (14.1)
9	6.13, d (1.8)	5.86, s	5.88, s	5.78, s	5.82, s	5.98, s	5.86, s
11					2.20, hept. (7.2)	2.07, hept (6.8)	2.12, m
12	2.07, d (1.8)	2.08, s	2.11, s	2.12, s	1.02, d (6.7)	0.96, d (6.8)	1.04, d (6.7)
13	1.88, br, s	1.90, s	1.91, s	1.92, s	0.75, d (6.7)	0.79, d (6.8)	0.81, d (6.8)
14	1.18, s	1.14, s	1.30, s	1.20, s	1.44, s	1.16, s	1.43, s
15	1.13, d (7.1)	1.02, d (6.7)	1.22, d (7.1)	1.39, s	0.94, d (6.7)	1.03, d (6.7)	1.34, s

a *Ov. Signals partially overlapped*.

#### Rhizoperemophilane B (2)

Colorless oil; ${[\alpha ]}_{\text{D}}^{25}$ +36.7 (*c* 0.12, MeOH); UV (MeOH) λ_max_ (log ε) 208 (3.66), 245 (3.80), 280 (3.44) nm; ECD (*c* = 8.0 × 10^−4^ M, MeOH) λ (Δε) 221 (+3.09), 241 (+4.16), 284 (−1.74) nm; IR (KBr) ν_max_ 3418, 2962, 2922, 1659, 1612, 1454, 1373, 1260, 1038, 897, 859, 802 cm^−1^; ^1^H NMR, and ^13^C NMR, see Tables 1, 2; HRESIMS *m*/*z* 251.1651 [M+H]^+^ (Calcd. for C_15_H_23_O_3_, 251.1642).

#### Rhizoperemophilane C (3)

Colorless amorphous solid; ${[\alpha ]}_{\text{D}}^{25}$ +38.4 (*c* 0.125, MeOH); UV (MeOH) λ_max_ (log ε) 203 (4.10), 242 (3.95), 284 (3.72) nm; ECD (*c* = 7.52 × 10^−4^ M, MeOH) λ (Δε) 243 (+6.10), 287 (-2.70) nm; IR (KBr) ν_max_ 3385, 2918, 2850, 1661, 1384, 1181, 1058 cm^−1^; ^1^H NMR, and ^13^C NMR, see Tables 1, 2; HRESIMS *m*/*z* 289.1419 [M+Na]^+^ (Calcd. for C_15_H_22_O_4_Na, 289.1416).

#### Rhizoperemophilane D (4)

Colorless oil; ${[\alpha ]}_{\text{D}}^{25}$ +28.0 (*c* 0.1, MeOH); UV (MeOH) λ_max_ (log ε) 209 (3.64), 279 (3.62) nm; ECD (*c* = 8.06 × 10^−4^ M, MeOH) λ (Δε) 219 (+2.74), 244 (+0.57), 269 (+1.51), 310 (−0.67) nm; IR (KBr) ν_max_ 3420, 2922, 1714, 1680, 1648, 1610, 1453, 1376, 1296, 1204, 1141, 1026, 895, 800, 578, 427 cm^−1^; ^1^H NMR, and ^13^C NMR, see Tables 1, 2; HRESIMS *m*/*z* 249.1482 [M+H]^+^ (Calcd. for C_15_H_21_O_3_, 249.1485), 271.1302 [M+Na]^+^ (Calcd. for C_15_H_20_O_3_Na, 271.1305).

#### Rhizoperemophilane E (5)

Colorless oil; ${[\alpha ]}_{\text{D}}^{25}$ +45.3 (*c* 0.15, MeOH); UV (MeOH) λ_max_ (log ε) 237 (3.95) nm; ECD (*c* = 7.94 × 10^−4^ M, MeOH) λ (Δε) 231 (-3.77), 263 (+0.35), 331 (-1.10) nm; IR (KBr) ν_max_ 3444, 2966, 2929, 2876, 1680, 1621, 1468, 1385, 1273, 1145, 1126, 1038, 1015, 900, 871, 419 cm^−1^; ^1^H NMR, and ^13^C NMR, see Tables 1, 2; HRESIMS *m*/*z* 253.1798 [M+H]^+^ (Calcd. for C_15_H_25_O_3_, 253.1798), 275.1616 [M+Na]^+^ (Calcd. for C_15_H_24_O_3_Na, 275.1618).

#### Rhizoperemophilane F (6)

Colorless oil; ${[\alpha ]}_{\text{D}}^{25}$ +51.2 (*c* 0.25, MeOH); UV (MeOH) λ_max_ (log ε) 234 (4.43), 288 (3.43) nm; ECD (*c* = 8.0 × 10^−4^ M, MeOH) λ (Δε) 227 (-1.30), 247 (+3.49), 325 (-1.39) nm; IR (KBr) ν_max_ 3482, 2971, 2876, 1715, 1679, 1625, 1451, 1386, 1266, 1129, 1093, 1019, 872, 797, 587 cm^−1^; ^1^H NMR, and ^13^C NMR, see Tables 1, 2; HRESIMS *m*/*z* 251.1642 [M+H]^+^ (Calcd. for C_15_H_23_O_3_, 251.1642), 273.1461 [M+Na]^+^ (Calcd. for C_15_H_22_NaO_3_, 273.1461).

#### Rhizoperemophilane G (7)

Colorless oil; ${[\alpha ]}_{\text{D}}^{25}$ +204.8 (*c* 0.125, MeOH); UV (MeOH) λ_max_ (log ε) 203 (3.92), 274 (4.15) nm; ECD (*c* = 7.52 × 10^−4^ M, MeOH) λ (Δε) 209 (+5.99), 274 (+7.92), 356 (+0.71) nm; IR (KBr) ν_max_ 3386, 2926, 2850, 1716, 1662, 1631, 1457, 1385, 1273, 1241, 1202, 1041, 891, 686 cm^−1^; ^1^H NMR, and ^13^C NMR, see Tables 1, 2; HRESIMS *m*/*z* 265.1435 [M-H]^−^ (Calcd. for C_15_H_21_O_4_, 265.1445).

#### Rhizoperemophilane H (8)

Colorless oil; ${[\alpha ]}_{\text{D}}^{25}$ +8.0 (*c* 0.2, MeOH); UV (MeOH) λ_max_ (log ε) 242 (3.87) nm; ECD (*c* = 7.46 × 10^−4^ M, MeOH) λ (Δε) 225 (−0.11), 252 (+0.87), 305 (+0.05), 316 (+0.11), 348 (−0.10) nm; IR (KBr) ν_max_ 3421, 2967, 1668, 1466, 1373, 1204, 1139, 1114, 1071, 1017, 976, 909, 587 cm^−1^; ^1^H NMR, and ^13^C NMR, see Tables 1, 3; HRESIMS *m*/*z* 269.1746 [M+H]^+^ (Calcd. for C_15_H_25_O_4_, 269.1747).

**Table 3:** ^1^H (400 MHz) NMR data of **8**~**14** [δ_H_, mult. (*J* in Hz)].

**Position**	**8^a^**	**9^a^**	**10^a^**	**11^a^**	**12^a^**	**13^a^**	**14^a^**	**14^c^**
1	4.74, dd (12.6, 5.2)	2.88, m 2.79, m	3.94, d (4.2)		6.96, s	6.04, s	5.88, s	5.90, s
2	2.31, dt (12.7, 4.5) 1.66, m	2.64, ddd (14.3, 12.3, 7.1) 2.47, ddd (14.3, 6.2, 3.6)	3.35, d (4.2)					
3	3.95, q (3.2)		3.90, br. s			5.46, d (4.4)		
4	1.61, m	2.73, q (6.8)				2.39, qd (7.0, 4.4)		
6	1.92, d (14.7) 1.77, d (14.7)	2.02, d (14.6) 1.88, d (14.6)	2.38, d (14.0) 1.70, d (14.0)	3.25, d (16.4) 2.42, d (17.4)	3.26, d (16.8) 2.74, d (16.8)	2.90, d (16.3) 2.33, br. d (16.3)	2.98, br. d (15.8) 2.80, d (16.7)	2.78, br. s
9	6.18, s	5.95, d (1.7)	6.39, s	6.68, s		6.01, s	6.05, s	6.00, s
11	2.18, hept (6.9)	2.29, hept (6.8)						
12	0.88, d (6.9)	0.93, d (6.8)	0.94, s		7.77, s			
13	0.82, d (6.9)	0.84, d (6.8)	1.34, s	1.97, s	2.09, s	1.95, d (2.1)	1.90, d (1.8)	1.82, s
14	1.41, s	1.15, s	1.21, s	1.32, s	1.39, s	1.29, s	1.24, s	1.11, s
15	1.15, d (7.0)	1.07, d (6.7)	1.55, s	2.07, s	2.11, s	1.14, d (7.0)	1.62, s	1.52, s
-NH						8.05, s		10.30, s
4-OH								7.26, s
3-OAc						2.12, s		

Recorded in

a *CD_3_OD*;

b *CDCl_3_*;

c *DMSO-d_6_*.

#### Rhizoperemophilane I (9)

Colorless oil; ${[\alpha ]}_{\text{D}}^{25}-$8.0 (*c* 0.05, MeOH); UV (MeOH) λ_max_ (log ε) 236 (3.76) nm; ECD (*c* = 8.0 × 10^−4^ M, MeOH) λ (Δε) 223 (+0.34), 247 (+0.34), 325 (−0.47) nm; IR (KBr) ν_max_ 3411, 2960, 2923, 2852, 1723, 1660, 1462, 1409, 1384, 1273, 1147, 1014, 908, 595 cm^−1^; ^1^H NMR, and ^13^C NMR, see Tables 1, 3; HRESIMS *m*/*z* 251.1635 [M+H]^+^ (Calcd. for C_15_H_23_O_3_, 251.1642), 273.1462 [M+Na]^+^ (Calcd. for C_15_H_22_O_3_Na, 273.1461).

#### Rhizoperemophilane J (10)

Colorless amorphous solid; ${[\alpha ]}_{\text{D}}^{25}$ −186.67 (*c* 0.12, MeOH); UV (MeOH) λ_max_ (log ε) 249 (3.92) nm; ECD (*c* = 7.14 × 10^−4^ M, MeOH) λ (Δε) 209 (−7.72), 233 (−2.68), 261 (−10.03), 329 (+0.75) nm; IR (KBr) ν_max_ 3525, 3565, 3022, 2973, 2934, 1678, 1611, 1472, 1414, 1386, 1372, 1271, 1239, 1162, 1152, 1117, 1048, 1002, 973, 880, 816, 761, 664, 570, 515 cm^−1^; ^1^H NMR, and ^13^C NMR, see Tables 1, 3; HRESIMS *m*/*z* 279.1240 [M–H]^−^ (Calcd. for C_15_H_19_O_5_, 279.1238).

#### Rhizoperemophilane K (11)

Brown amorphous solid; ${[\alpha ]}_{\text{D}}^{25}-$232.73 (*c* 0.11, MeOH); UV (MeOH) λ_max_ (log ε) 208 (3.91), 276 (3.89), 318 (3.74), 397 (3.83) nm; ECD (*c* = 7.30 × 10^−4^ M, MeOH) λ (Δε) 224 (−3.60), 248 (+1.28), 282 (−3.72), 338 (−0.58) nm; IR (KBr) ν_max_ 3389, 2923, 1771, 1680, 1610, 1453, 1430, 1373, 1337, 1197, 1134, 1095, 1052, 1017, 846, 800, 564, 523 cm^−1^; ^1^H NMR, and ^13^C NMR, see Tables 1, 3; HRESIMS *m*/*z* 273.0770 [M-H]^−^ (Calcd. for C_15_H_13_O_5_, 273.0768).

#### Rhizoperemophilane L (12)

Yellowish oil; ${[\alpha ]}_{\text{D}}^{25}$ +244.0 (*c* 0.1, MeOH); UV (MeOH) λ_max_ (log ε) 208 (3.93), 248 (3.95), 318 (3.89) nm; ECD (*c* = 7.75 × 10^−4^ M, MeOH) λ (Δε) 206 (−1.83), 231 (+7.30), 255 (−5.95), 301 (−1.89), 363 (+4.38) nm; IR (KBr) ν_max_ 3408, 2920, 2850, 1671, 1643, 1461, 1417, 1203, 1111, 1028, 579 cm^−1^; ^1^H NMR, and ^13^C NMR, see Tables 1, 3; HRESIMS *m*/*z* 259.0962 [M+H]^+^ (Calcd. for C_15_H_15_O_4_, 259.0965), 281.0788 [M+Na]^+^ (Calcd. for C_15_H_14_O_4_Na, 281.0784).

#### Rhizoperemophilane M (13)

Greenish-yellow amorphous solid; ${[\alpha ]}_{\text{D}}^{25}-$167.27 (*c* 0.11, MeOH); UV (MeOH) λ_max_ (log ε) 207 (3.86), 358 (4.16) nm; ECD (*c* = 9.97 × 10^−4^ M, MeOH) λ (Δε) 223 (−0.57), 248 (+1.60), 314 (+0.47), 343 (+1.58), 392 (−3.41) nm; IR (KBr) ν_max_ 3421, 3282, 2967, 2919, 2851, 1746, 1724, 1664, 1624, 1566, 1374, 1241, 1023, 910, 846, 800, 578, 420 cm^−1^; ^1^H NMR, and ^13^C NMR, see Tables 1, 3; HRESIMS *m*/*z* 300.1237 [M-H]^−^ (Calcd. for C_17_H_18_NO_4_, 300.1241).

#### Rhizoperemophilane N (14)

Light-yellowish amorphous solid; ${[\alpha ]}_{\text{D}}^{25}$ −244.0 (*c* 0.1, MeOH); UV (MeOH) λ_max_ (log ε) 208 (3.88), 342 (4.40) nm; ECD (*c* = 7.66 × 10^−4^ M, MeOH) λ (Δε) 233 (−3.26), 276 (+3.57), 332 (−9.11) nm; IR (KBr) ν_max_ 3233, 2919, 2851, 1714, 1681, 1631, 1454, 1390, 1350, 1300, 1198, 1174, 1125, 1067, 958, 885, 837, 672, 559 cm^−1^; ^1^H NMR, and ^13^C NMR, see Tables 1, 3; HRESIMS *m*/*z* 284.0889 [M+Na]^+^ (Calcd. for C_14_H_15_NO_4_Na, 284.0893).

### ECD Calculation

The Merck Molecular Force Field (MMFF) conformational searches, geometry optimization, and frequency calculations of the MMFF conformers using the DFT method at the B3LYP/6-31 G(d) level *in vacuo* were performed as described previously (Meng et al., 2019). TDDFT ECD calculations of the low-energized conformers (≥1%) without imaginary frequencies were carried out at the B3LYP/6-31+G(d) level with the polarizable continuum model (PCM) for MeOH. In addition, two further levels (PBE0/TZVP, BH&HLYP/TZVP) were used if required. The ECD spectrum of each conformer was simulated by the program SpecDis (Bruhn et al., 2013) using a Gaussian band shape with an exponential half-width σ of 0.25–0.5 eV, using the dipole-length computed rotational strengths. The Boltzmann-averaged ECD spectrum was generated according to the equilibrium population of each conformer at 298.15 K, which was calculated from its relative Gibbs free energies. The generated spectra were then compared with the experimental data to determine the absolute configuration. The calculated ECD spectra were UV-shifted and scaled for a better comparison with the measured spectrum.

### Optical Rotation Calculation

The B3LYP/6-31 G(d)-optimized conformer of (5*R*)-**11** was used to calculate the optical rotation. The calculation was carried out using the time-dependent DFT method at the B3LYP/6-31+G(d,p) level (PCM = MeOH), as described previously (Lai et al., 2020).

### Antibacterial Assay

The antibacterial activities of the isolate compounds (except **3** and **19**) were tested against six pathogenic bacteria including *Agrobacterium tumefaciens, Bacillus subtilis, Pseudomonas lachrymans, Ralstonia solanacearum, Staphylococcus haemolyticus*, and *Xanthomonas vesicatoria* using the modified broth micro-dilution-MTT assay as described previously (Shan et al., 2014; Lai et al., 2016). The bacteria were grown in liquid LB medium overnight at 28°C, and the diluted bacterial suspension (10^6^ cfu/mL) was used for the assay. Streptomycin sulfate was used as the positive control.

### Cytotoxic Assay

Cytotoxicity of **1**, **2**, **4**~**6**, and **8**~**14** was tested against the human carcinoma cells using the microculture tetrazolium (MTT) assay as described previously (Sun et al., 2017). The tested cell lines included gastric cancer cells (BGC-823), desmoplastic cerebellar medulloblastoma cells (Daoy), colon cancer cells (HCT-116), liver hepatocellular carcinoma cells (HepG2), and non-small-cell lung carcinoma cells (NCI-H1650). Taxol was used as the positive control, which showed cytotoxicity against these cells with IC_50_ (μM) values of 1.2 × 10^−4^, 0.74 × 10^−3^, 0.9 × 10^−3^, 0.75 × 10^−2^, and 0.2 × 10^−3^, respectively.

### Phytotoxic Assay

Compounds **1**, **2**, **4**~**6**, **8**, **10**~**14**, **16**, and **19**~**22** were evaluated for their inhibitory activities on the radicle elongation of rice (*Oryza sativa*) seedlings as described previously (Sun et al., 2017). The seeds of the rice variety Dannuo 2 were used. Compounds were dissolved in water containing 2.5% DMSO. The solvent was used as the negative control, and glyphosate [*N*-(phosphonomethyl) glycine] was used as the positive control. The length of the radicle and germ of each germinated seed was measured after 48 h. The inhibition rate was calculated as follows: inhibition (%) = [(Lc – Lt)/Lc] ×100, where Lc/Lt is the length of the control/treated group.

## Results and Discussion

**Figure 1:**
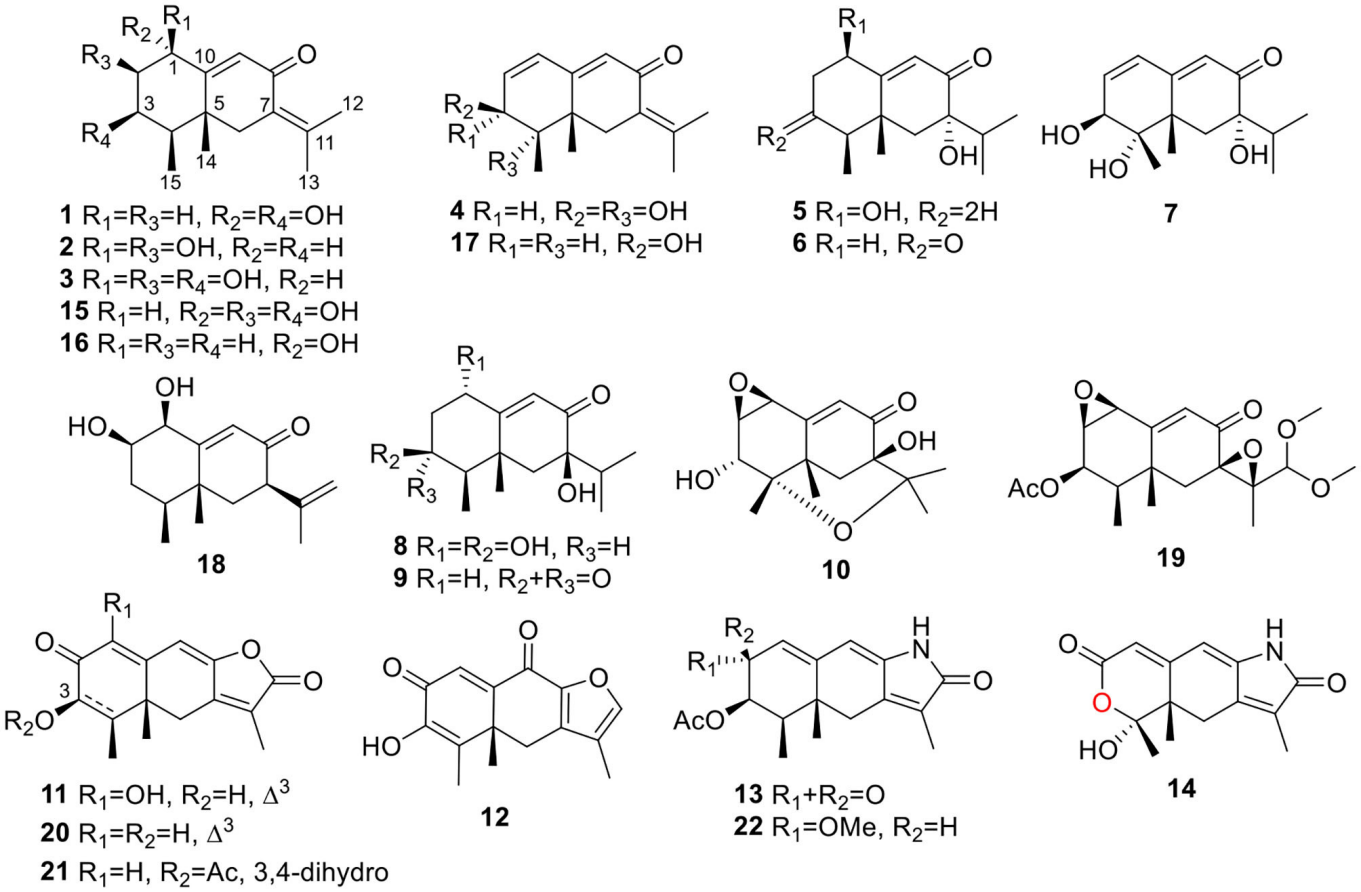
Structures of the isolated compounds (**1**~**22**).

The fungal extract was subjected to column chromatography over silica gel, Sephadex LH-20, and ODS, and purified by semi-preparative HPLC to afford compounds **1**~**22** (Figure 1).

Rhizoperemophilane A (**1**) was isolated as a colorless oil. Its molecular formula was determined as C_15_H_22_O_3_ by HRESIMS, indicating five degrees of unsaturation. Its IR spectrum showed absorptions corresponding to the hydroxyl group (3403 cm^−1^), conjugated keto group (1656 cm^−1^), and olefinic group (1624 cm^−1^). This conjugated system was also inferred from the UV absorption spectrum (λ_max_ 250, 280 nm). The ^13^C NMR spectrum (Table 1) displayed fifteen carbon resonances that could be assigned to one keto group (δ_C_ 194.4), two C=C double bonds (δ_C_ 174.0, 145.2, 128.9, 122.6), two oxygenated sp^3^ carbons (δ_C_ 72.3, 65.2), and eight non-oxygenated sp^3^ carbons including four methyl groups in the high-field region (δ_C_ 22.8, 22.3, 20.2, 12.3). These functionalities account for three degrees of unsaturation, thus hinting the bicyclic nature of **1**. Analysis of the ^1^H NMR spectrum (Table 2) revealed the presence of four methyl groups, including one singlet (δ_H_ 1.18) and one doublet (δ_H_ 1.13, d, *J*=7.1 Hz) in the high-field region and two olefinic methyl groups (δ_H_ 1.88, 2.07). These data were characteristic of an eremophilane-type of sesquiterpenes, i.e., fifteen carbons in a bicyclic structure containing four methyl groups, among which one connected to a methine group (CH-4), one connected to the quaternary carbon (C-5), resulting in one doublet (Me-15) and one singlet (Me-14) signal, and two other methyl groups (Me-12 and 13) derived from the isopropyl group (Figure 1). In addition, the signals for one olefinic proton (δ_H_ 6.13), two oxymethine protons (δ_H_ 4.63, 3.95), and six aliphatic protons could be seen from the ^1^H NMR spectrum, which in combination of the ^13^C NMR spectrum suggested the occurrence of one trisubstituted and one tetrasubstituted double bond, and two oxygenated methine groups.

**Figure 2:**
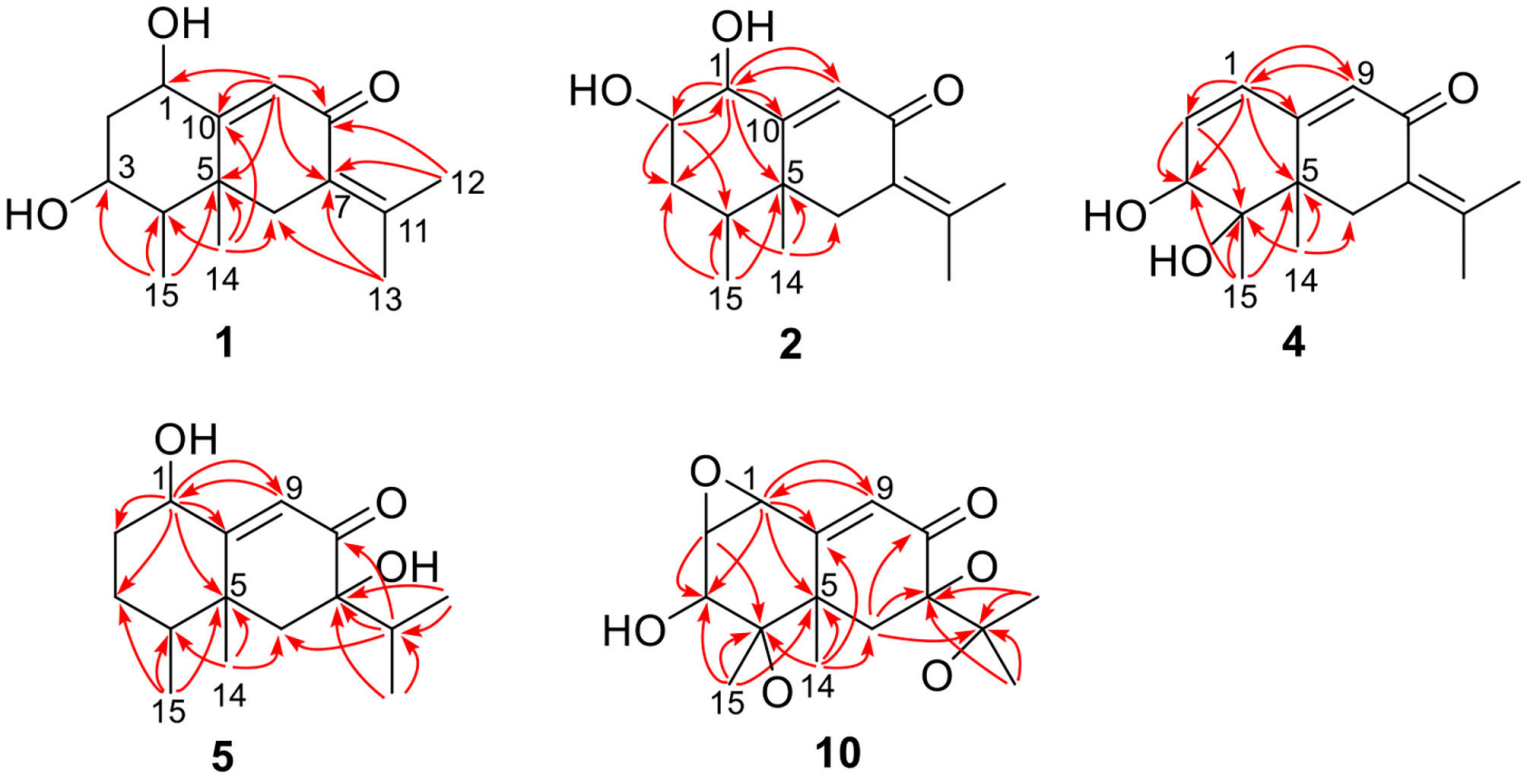
Selected HMBC correlations of **1**, **2**, **4**, **5**, and **10**.

The positions of these functional groups in the eremophilane skeleton were clarified by analysis of the HMBC spectrum (Figure 2). The olefinic methyl groups (Me-12/13, δ_H_ 2.07, 1.88) showed correlations to the tetrasubstituted C=C (δ_C_ 145.2, 128.9), allowing the assignment of this double bond to Δ^7(11)^. The long-range correlations from these methyl groups to the keto group (δ_C_ 194.4, C-8), and the methylene group (C-6, δ_C_ 43.7), the correlations from Me-14 (δ_H_ 1.18, s) to C-4 (δ_C_ 47.1), C-5 (δ_C_ 43.5), C-6, and C-10 (δ_C_ 174.0), and the correlations from the olefinic proton (H-9, δ_H_ 6.13) to C-5, C-7 (δ_C_ 128.9), C-8, and C-10, suggested the α,β-unsaturated keto group locating at C-8~C-10. Further correlation from H-9 to the oxygenated methine (δ_H_ 4.63, δ_C_ 65.2) indicates one hydroxyl group substituting at C-1, while the other hydroxyl group was linked to C-3 as inferred from the correlation between Me-15 (δ_H_ 1.13, d) and the second oxygenated methine (δ_H_ 3.95, δ_C_ 72.3). Thus, compound **1** has a planar structure of 1,3-dihydroxyl-7(11),9-eremophiladien-8-one.

**Figure 3:**
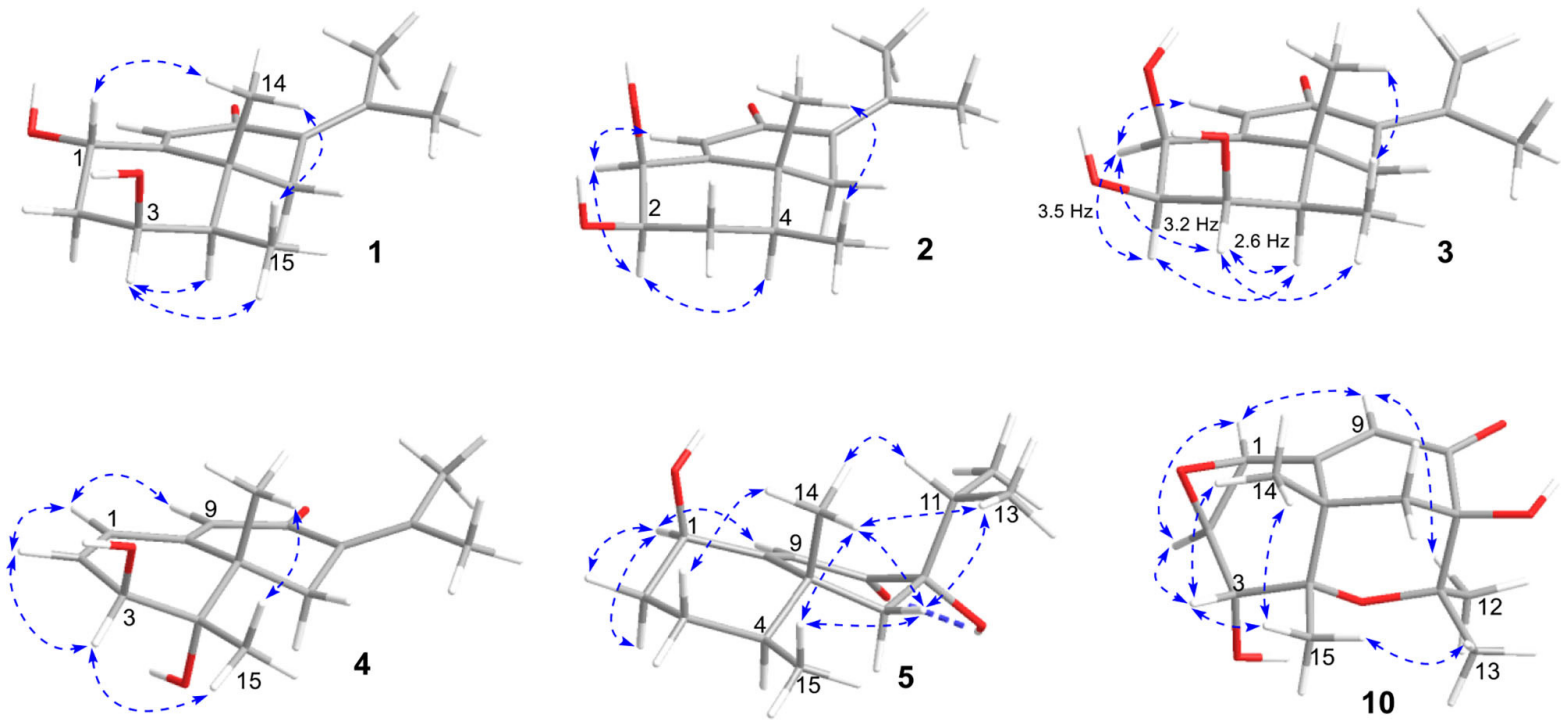
Selected NOESY correlations of **1**~**5**, and **10**.

The eremophilane type of sesquiterpenoids is usually rigid in conformation and adopts chair or pseudo-chair conformation for the six-membered ring (Niu et al., 2018), so the relative configuration of **1** could be determined by analysis of the ^1^H-^1^H coupling constants (^3^*J*) and NOESY spectrum (Figure 3). In the chair conformation of **1**, the large coupling constant of 12.6 Hz between H-1 (δ_H_ 4.63, ddd) and H-2b (δ_H_ 1.65) indicates both protons are axial, whereas only small ^3^*J* values were found between H-3/H_2_-2 (3.3, 2.8 Hz) and H-3/H-4 (2.9 Hz), suggesting the equatorial orientation of H-3 (δ_H_ 3.95, ddd). The NOESY correlation between H-1 and Me-14 (δ_H_ 1.18, s) revealed that this methyl group was axial as well, while the correlation seen between Me-14 and Me-15 (δ_H_ 1.13, d) positioned the latter to the equatorial site. Such arrangement of the 4,5-dimethyl groups (i.e., both β-configurated, with one axial and one equatorial) was conserved in almost all found fungal eremophilanoids, though with a few exceptions (Yuyama et al., 2017). From this aspect, we might deduce the absolute configuration of **1** as shown in Figure 1.

**Figure 4:**
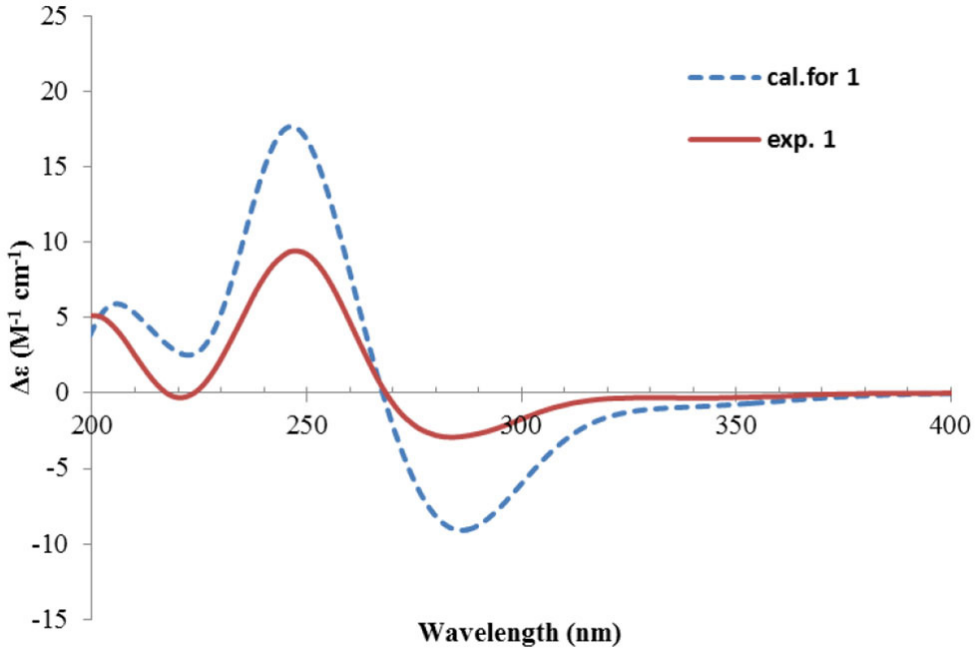
Experimental and calculated ECD spectra of **1**.

Nevertheless, solid evidence to support the absolute configuration assignment was required. As compound **1** displayed Cotton effects at 284 (Δε −2.93, n→π^*^) and 248 (Δε +9.40, π→π^*^) nm in its electronic circular dichroism (ECD) spectrum due to the presence of the α,β-unsaturated keto chromophore, the absolute configuration of **1** was thus determined by quantum chemical ECD calculations. Starting from the input structure (1*S*, 3*S*, 4*R*, 5*R*)-**1**, the MMFF conformation search generated 12 conformers within a 5-kcal/mol energy window. These were then subjected to geometry optimization using the DFT method at the B3LYP/6-31g(d) level in the gas phase, resulting in six conformers with populations ≥1% (Supplementary Figure 1). These lower-energized conformers have the same conformation but with different orientations of the protons of the 1,3-hydroxyl groups, and they displayed similar theoretical ECD spectra above 230 nm as expected. The overall calculated ECD spectrum fitted well with the experimental one (Figure 4), confirming the 1*S*, 3*S*, 4*R*, and 5*R* configuration of **1**.

Rhizoperemophilane B (**2**) was isolated as an isomer of **1**, and both shared the same molecular formula. The ^1^H and ^13^C NMR data (Tables 1, 2) were similar except for the cyclohexane ring, meaning that the substitution patterns were different. This was clarified by analysis of the HMBC spectrum (Figure 2). The correlation seen from the olefinic proton group (H-9, δ_H_ 5.86, s) to the oxymethine group (δ_C_ 76.4, δ_H_ 4.18) allowed us to assign the first hydroxyl group to C-1, while the correlations from H-1 to the second oxymethine group (CH-2, δ_C_ 72.3, δ_H_ 3.62), C-3 (δ_C_ 34.3), and C-5 (δ_C_ 41.7), from Me-15 (δ_H_ 1.02, d) to C-3, C-4 (δ_C_ 41.1), and C-5, and from H-2 to C-1, C-3, and C-4 suggested the second hydroxyl group locating at C-2. Hence, compound **2** has a 1,2-diol structure. The large ^3^*J* value (12.0 Hz) between H-2 and H-3ax was indicative of the axial orientation of H-2, then H-1 had to be equatorial due to the small ^3^*J*_H−1, H−2_ (3.1 Hz), unlike that of axial in **1**. Such change at C-1 could explain the large chemical shift discrepancy in C-9 (+8.1 ppm) and C-10 (-5.9 ppm) between both compounds, as well as the disappearance of the NOESY correlation between H-1 (δ_H_ 4.18) and Me-14 (δ_H_ 1.14). Meanwhile, the observed NOESY correlation of H-2 (δ_H_ 3.62)/H-4 (δ_H_ 1.54) was consistent with their 1,3-diaxial relationship (Figure 3).

**Figure 5:**
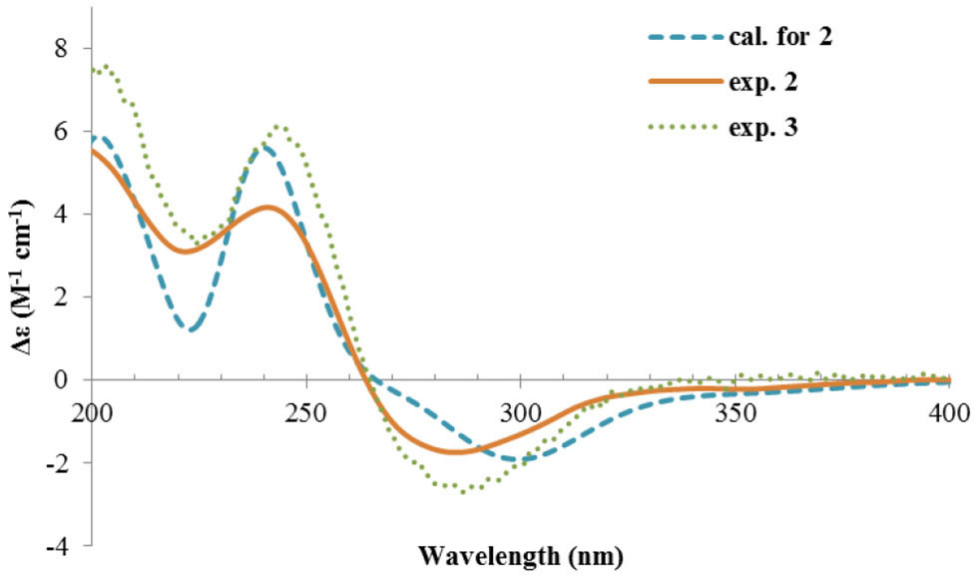
Experimental ECD spectra of **2** and **3**, and the calculated spectrum of **2**.

The ECD spectrum of **2** (Figure 5) displayed a similar Cotton effects at ~241, 284 nm, albeit the absorptions (Δε) were about half compared to those of **1** (Figure 4). Such similarity reflexed the same overall conformation of the bicyclic system; thus, the absolute structure of **2** was proposed as shown in Figure 1. Moreover, a quantum chemical ECD computation of **2** was performed as well. The calculated spectrum reproduced well the experimental data (Figure 5), confirming the 1*S*, 2*R*, 4*S*, and 5*R* absolute configuration of **2**.

Rhizoperemophilane C (**3**) was isolated as a congener of **2**, bearing one more oxygen atom than that of **2** in the molecular formula, as determined by HRESIMS. This suggested one more hydroxyl substitution in **3**. Indeed, the NMR data were quite similar for both; however, one additional oxymethine (δ_C_ 77.6, δ_H_ 3.87) was found at C-3 of **3** instead of a methylene group in **2**, which was confirmed by the HMBC measurement. Hence, compound **3** was a 3-hydoxylated derivative of **2**. The relative configuration of **3** was determined by comprehensive analysis of the coupling constants and NOESY correlations. As inferred from the small ^3^*J*_H−3, H−4ax_ (2.6 Hz), H-3 had to be equatorial, which was consistent with the observed NOESY correlations of H-3/H-4 and H-3/Me-15. The NOESY correlation of H-4/H-2 allowed the assignment of H-2 to the axial position. Meanwhile, H-2 showed a small coupling constant to H-1 (3.5 Hz) hinting the equatorial H-1, which was corroborated by the observed NOESY cross-peak between them. Hence, compound **3** was elucidated to be the 3β-hydroxylated derivative of **2**. As a similar ECD profile was found between both (Figure 5), the absolute configuration of **3** was likewise determined, as shown in Figure 1.

Rhizoperemophilane D (**4**) had a molecular formula of C_15_H_20_O_3_ as deduced from HRESIMS. Inspection of the ^1^H and ^13^C NMR data (Tables 1, 2) revealed that it shared the same substitution pattern for the eastern ring as those of **1**~**3**; however, they differed in the western ring. In **4**, one additional disubstituted C=C bond (δ_H_ 6.27, d/δ_C_ 129.06; δ_H_ 6.17, dd/δ_C_ 136.1), one oxymethine (δ_H_ 3.93, δ_C_ 74.3), and one oxygenated quaternary carbon (δ_C_ 75.0) were present in the western ring. The position of these functional groups was elucidated by analysis of the HMBC (Figure 2) and NOESY (Figure 3) spectra. The HMBC correlation from H-9 (δ_H_ 5.78, s) to the olefinic carbon (C-1), as well as from H-1 (δ_H_ 6.27, d) to C-9 (δ_C_ 129.1), and C-10 (δ_C_ 162.1), allowed the assignment of the C=C bond to Δ^1(2)^, which was consistent with the NOESY correlation between H-9/H-1. A same coupling (*J* = 5.0 Hz) between olefinic H-2 (δ_H_ 6.17, dd) and the oxymethine proton (δ_H_ 3.93, d) inferred the latter group at C-3, which was corroborated by the observed HMBC correlations of H-1/C-3 (δ_C_ 74.3) and H-2/C-3. Then, HMBC correlations from Me-15 (δ_H_ 1.39, s) to C-3, the oxygenated quaternary carbon (C-4, δ_C_ 75.0), and C-5 (δ_C_ 44.6), and from Me-14 (δ_H_ 1.20, s) to C-4, C-5, and C-6 (δ_C_ 35.9), suggested that Me-15 was connected to the oxygen-bearing quaternary carbon. The relative configuration of **4** was determined by analysis of the NOESY spectrum (Figure 3). In the half-chair conformation of the cyclohexene ring, Me-14 and Me-15 oriented to the axial and equatorial position, respectively, commonly seen in eremorphilanes, while the correlation observed between Me-15 and H-3, but not between H-3 and Me-14, defined the equatorial location of H-3. Hence, compound **4** was elucidated to be 3β,4α-dihydroxyl-1,7(11),9-eremophilatrien-8-one, which was the first 4-oxygenated eremophilanoid.

**Figure 6:**
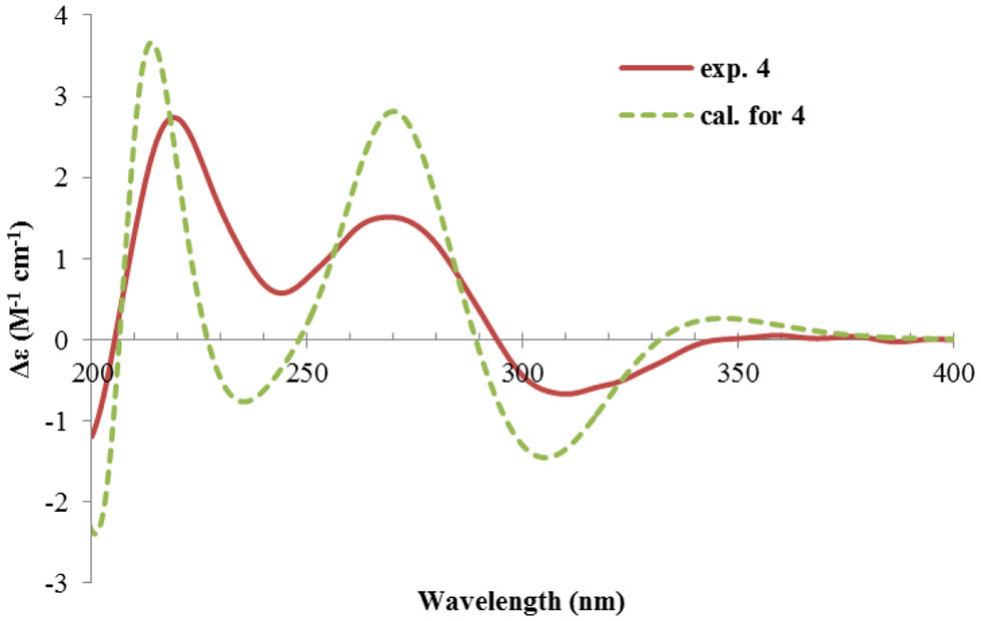
Experimental and calculated ECD spectra of **4**.

The absolute configuration of **4** was determined by quantum chemical ECD computations. The lower-energized conformers differed only in the orientations of the protons of the hydroxyl groups at C-3 and C-4, but the overall conformation was the same, leading to a similar ECD profile for each, albeit with different intensity. The ECD calculations for these conformers were performed using different functions (Supplementary Figure 4). Among them, the BH&HLYP/TZVP-calculated spectrum showed the best match to the experimental data (Figure 6). Therefore, the absolute configuration of **4** was determined as 3*S*, 4*S*, 5*S*.

Rhizoperemophilane E (**5**) was isolated as a colorless oil, with molecular formula of C_15_H_24_O_3_. It displayed UV maximum absorption only at 237 nm, indicating a shorter conjugated system than those of **1**~**4**. As expected, only the signals for an αβ-unsaturated keto group (δ_C_ 205.2, 123.5, 169.3, for C-8~C-10), but no additional C=C resonances were seen in the ^13^C NMR data (Table 1). In addition, an isolated isopropyl group was found (δ_H_ 2.20, 1H, hept; δ_H_ 0.75, 3H, d; δ_H_ 1.02, 3H, d), together with the HMBC correlations from both methyl groups to the oxygenated quaternary carbon (δ_C_ 78.9, C-7), suggesting that C_7_=C_11_ was replaced by a sigma bond and C-7 was hydroxylated in **5**. Moreover, one oxymethine group was found (δ_H_ 4.29, br. s; δ_C_ 74.3, CH), which was located at C-1, as inferred from the HMBC correlations from H-9 (δ_H_ 5.82, s) to this group, as well as from H-1 (δ_H_ 4.29, br. s) to C-9 (δ_C_ 123.5) and C-10 (δ_C_ 169.3) (Figure 2). The relative configuration was established by analysis of the NOESY spectrum (Figure 3). The correlations between H-11 (δ_H_ 2.20, hept.), Me-13 (δ_H_ 0.75, d), and Me-14 (δ_H_ 1.44, s), as well as between Me-14 and Me-15 (δ_H_ 0.94, d), suggested that these groups were oriented to a similar face (β), while the lack of correlation between H-1 and Me-14, together with the fact that H-1 displayed small ^3^*J* values to the vicinal methylene protons, revealed the equatorial orientation of H-1. So compound **5** was deduced to be 1β,7α-dihydroxyl-eremophila-9-en-8-one.

**Figure 7:**
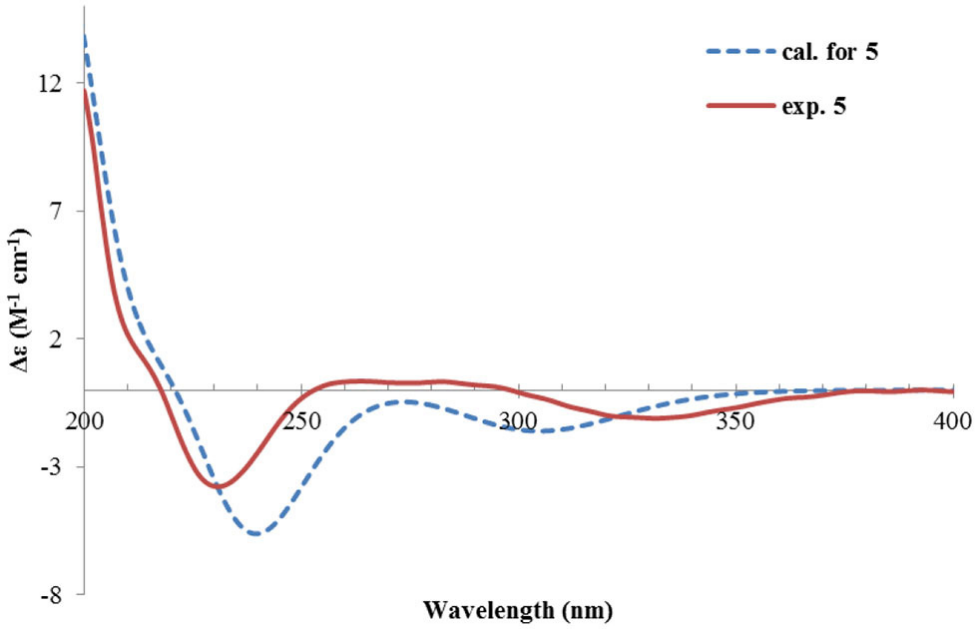
Experimental and calculated ECD spectra of **5**.

**Figure 8:**
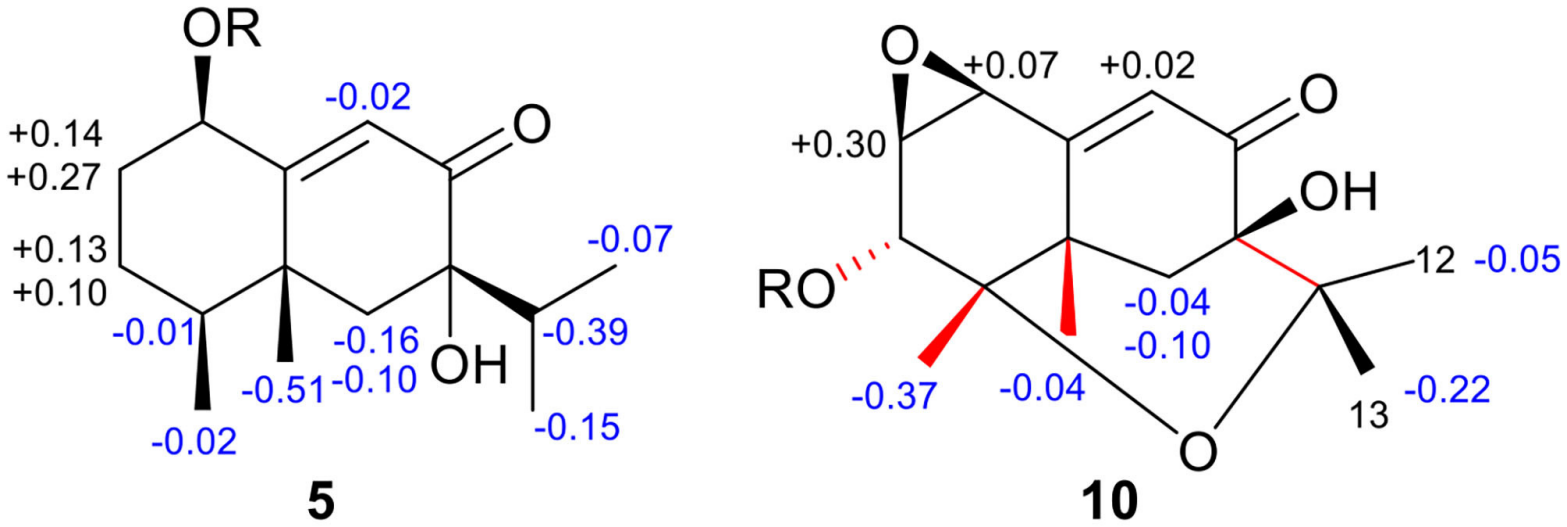
Δδ^RS^ (= δ_R_-δ_S_) values for the (*R*/*S*)-MPA esters of **5** and **10**.

The absolute configuration was determined by comparison of the calculated ECD spectrum with the experimental one. As shown in Figure 7, the calculated spectra for (1*R*, 4*S*, 5*R*, 7*S*)-**5** matched well the measured spectrum; thus, the absolute structure of **5** was established (Figure 1). Meanwhile, the absolute configuration of C-1 was independently determined by using the modified Mosher's method (Ohtani et al., 1991; Seco et al., 2004). By reacting with (*R*)- or (*S*)-α-methoxy-α-phenylacetic acid (MPA), **5** was converted to the corresponding (*R*)/(*S)*-MPA esters (**5R**/**5S**) (Supplementary Figure 6). Analysis of the discrepancy in the ^1^H NMR data (Δδ^RS^ = δ**5R**-δ**5S**) indicated the 1*R* configuration (Figure 8), such that the 4*S*, 5*R*, 7*S* configuration for the other stereocenters was confirmed by the established relative configuration.

**Figure 9:**
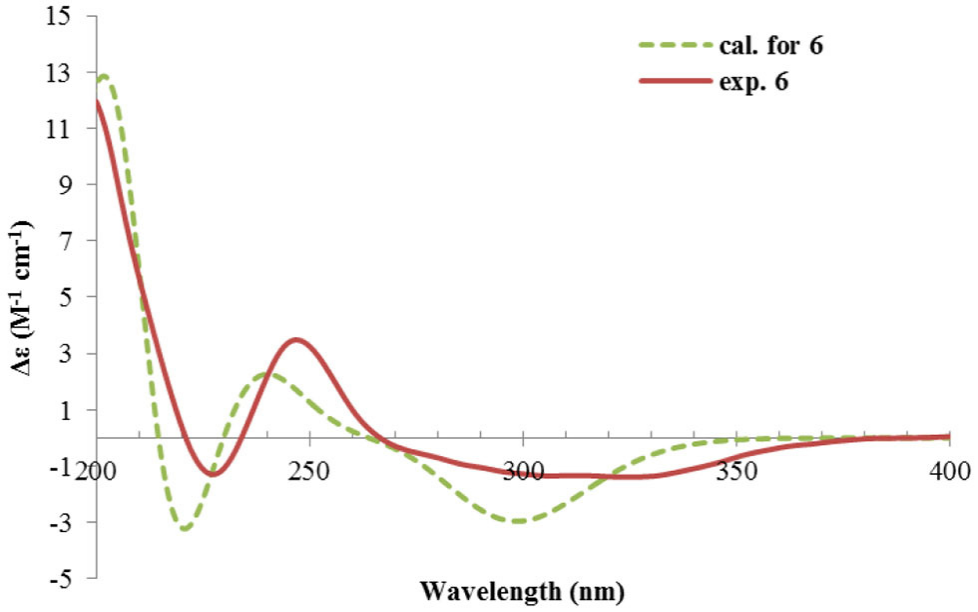
Experimental and calculated ECD spectra of **6**.

Rhizoperemophilane F (**6**) had a molecular formula of C_15_H_22_O_3_, being two protons less than that of **5**. Comparison of the NMR data (Tables 1, 2) revealed their great similarity in the eastern part; however, large differences were found in the western ring. Notably, no oxymethine signals were seen, while a keto group (δ_C_ 211.4) was present in **6**. This group was found to position at C-4, by the key HMBC correlation from Me-15 (δ_H_ 1.03, d) to it. The similarity of the NMR data and NOESY correlations suggested that they shared a similar relative configuration. However, the presence of one additional keto group at C-4 made assignment of the absolute configuration of **6** via direct comparison of its ECD spectrum with that of **5** unfruitful. So the TDDFT ECD calculations for (4*R*, 5*R*, 7*S*)-**6** were performed. Three different functions and basis set (B3LYP/6-31+g(d), BH&HLYP/TZVP, PBE0/TZVP) were used, with the solvent model PCM=MeOH. The calculated spectra well reproduced the ECD absorptions of **6** (Supplementary Figure 8), among which the BH&HLYP/TZVP-calculated one gave the best match (Figure 9). Hence, compound **6** was elucidated to be the 1-deoxy-4-oxo derivative of **5**.

**Figure 10:**
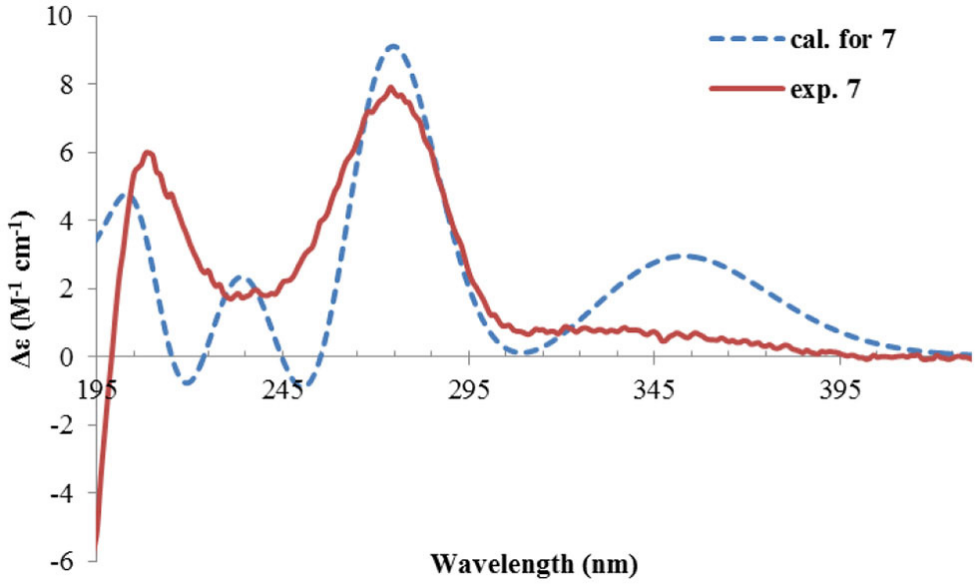
Experimental and calculated ECD spectra of **7**.

The NMR data (Tables 1, 2) of rhizoperemophilane G (**7**) were quite similar to those of compound **4**, except that an isopropyl and one oxygenated quaternary carbon in **7** replaced the dimethylethylene group of **4**. Meanwhile, the eastern part of **7** was deduced to be identical to that of either **5** or **6**, which was consistent with the almost superimposed NMR data for this part. The HMBC correlations from methine proton (H-11, δ_H_ 2.11) of the isopropyl group to C-6 (δ_C_ 39.5), C-7 (δ_C_ 78.6), and the keto (C-8, δ_C_ 203.6), as well as from both methyls (Me-12/13, δ_H_ 1.04, 0.81, each d) to C-7 confirmed this deduction. Similar NOESY correlations and ^1^H-^1^H coupling constants between **7** and **4** suggested the same relative configuration for C-3~C-5, while the NOESY correlations between Me-14 (δ_H_ 1.43, s), H-11, and Me-12 indicated that the isopropyl group co-faced with Me-14 (β-configurated), like that in **5** and **6**. The absolute configuration of **7** was determined by comparison the calculated ECD spectrum with the measured one (Figure 10). The result revealed the *S* absolute configuration for each chiral center in **7**.

**Figure 11:**
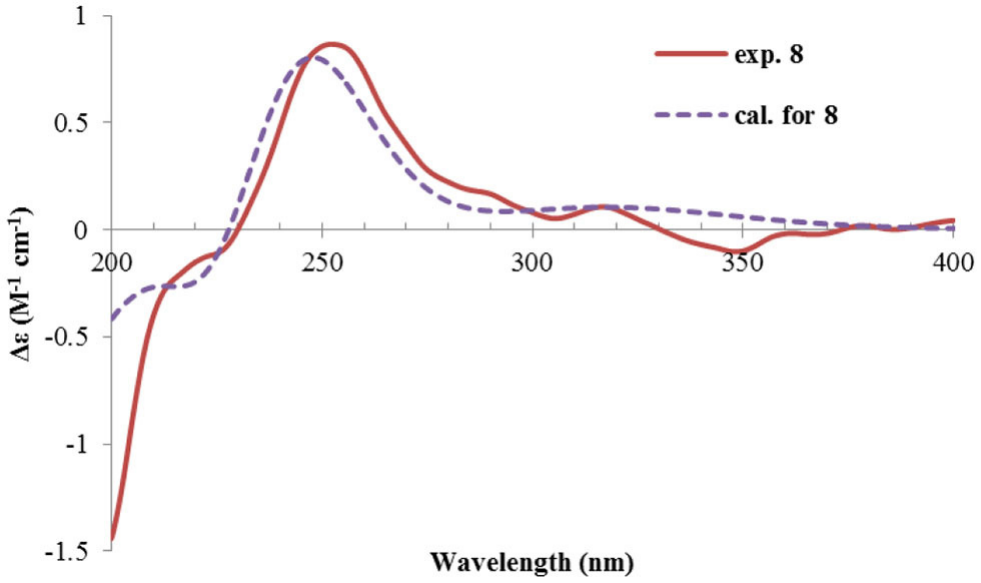
Experimental and calculated ECD spectra of **8**.

Rhizoperemophilane H (**8**) was isolated as a colorless oil, having a molecular formula as C_15_H_24_O_4_, with one oxygen being more than that of **5**. Inspection of the NMR data revealed that both contained an αβ-unsaturated keto group (C-8~C-10), an isopropyl group (C-11~C-13), and one oxygenated quaternary carbon (C-7); however, two oxymethine groups in **8** instead of one in **5** were found. The HMBC correlations from H-9 (δ_H_ 6.18, s) to one oxythine group (δ_C_ 65.5/δ_H_ 4.74, dd), compared to those from Me-15 (δ_H_ 1.15, d) to the other oxymethine (δ_C_ 72.0/δ_H_ 3.95, q), C-4 (δ_C_ 46.7), and C-5 (δ_C_ 41.4), allowed assignment of the oxymethines to C-1 and C-3, respectively. Hence, compound **8** has one more hydroxyl substitution at C-3 than **5**. The large coupling of 12.6 Hz between H-1 and one of the vicinal methylene protons (CH_2_-2) reflected the axial orientation of H-1, unlike that of equatorial in **5**. This was consistent with the observed NOESY correlation between H-1 and Me-14. By the same token, the small ^3^*J* (3.2 Hz) of H-3 with the neighboring protons suggested H-3 to be equatorial. Meanwhile, the ^13^C chemical shifts of the carbons around C-7 in **8** displayed large differences compared to those of **5**; for example, C-6 (**8** vs **5**, Δδ = −6.0 ppm), C-7 (−1.6), and C-8 (−2.4) were all upfield-shifted, indicating that the stereochemistry of C-7 might be changed. The NOESY correlation between H-11 and H-4 allowed to unambiguously assign the 7β-hydroxyl in **8**, as opposite to that of **5**. Thus, it has the structure of 1α, 3β, 7β-trihydroxyl-eremophila-9-en-8-one. The absolute configuration was determined by ECD calculations, and among the three methods used, the BH&HLYP-calculated spectrum gives the best match to the experimental data (Figure 11 and Supplementary Figure 11).

**Figure 12:**
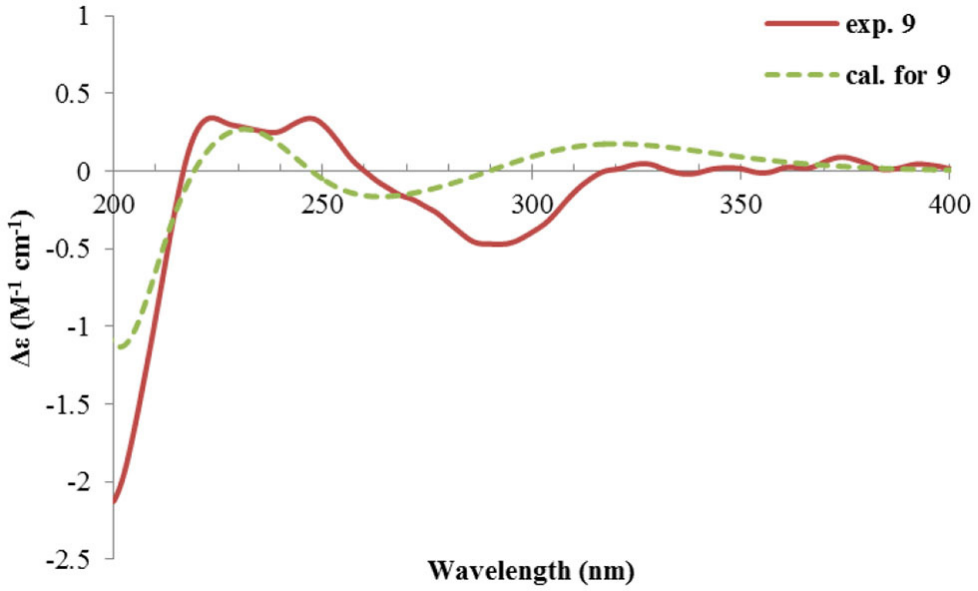
Experimental and calculated ECD spectra of **9**.

Rhizoperemophilane I (**9**) was isolated as the 7-epimer of **6**, both having a same molecular formula. Their NMR were almost superimposable to each other (Table 1), except for Me-14 and those around C-7. The Δδ values (δ**9**-δ**6**, in ppm) for those carbons were−3.5 (C-6),−1.5 (C-7),−1.8 (C-8), +1.2 (C-11), +1.3 (Me-13), and +2.5 (Me-14), respectively, which could be well explained by the reverse of the stereochemistry at C-7, like in the case of **8** vs **5**. This deduction was in agreement with the lack of NOESY correlation between H-11/Me-14, which, on the contrary, was clearly seen in those 7β-isopropyl containing congeners (**5**~**7**). The absolute configuration of **9** was determined also by ECD calculations (Figure 12), and the 4*R*, 5*R*, 7*R* configuration was confirmed.

Rhizoperemophilane J (**10**) was isolated as a colorless amorphous solid, and its molecular formula was determined as C_15_H_20_O_5_, with six degrees of unsaturation. Inspection of the NMR data (Tables 1, 3) revealed the presence of an αβ-unsaturated keto group (C-8~C-10, δ_C_ 200.0, 126.5, 168.7), one 1,2-epoxy group (δ_C_ 58.1, 61.3), three oxygenated quaternary carbons (δ_C_ 82.0, 77.3, 77.3), and one oxymethine group (δ_C_ 73.7; δ_H_ 3.90, br. s). The connection of these functional groups was established by detailed analysis of the 2D NMR spectra (Figure 2). The HMBC correlations from H-9 (δ_H_ 6.39, s) to C-1 (δ_C_ 58.1), and from H-1 (δ_H_ 3.94, d) to C-9 (δ_C_ 126.5), C-10 (δ_C_ 168.7), and C-5 (δ_C_ 44.5), were used to assign the 1,2-epoxy ring. The correlations from Me-15 (δ_H_ 1.55, s) to C-3 (δ_C_ 73.7), C-4 (δ_C_ 82.0), and C-5, and from Me-14 (δ_H_ 1.21, s) to C-4, C-5, C-10, and C-6 (δ_C_ 39.1), indicated that the oxymethine group was at C-3, while C-4 was an oxygenated quaternary carbon. In addition, Me-12/13 (δ_H_ 0.94/1.34, each s) and CH_2_-6 (δ_H_ 2.38/1.70, each d) were found to correlate with the two unassigned oxygenated quaternary carbons (δ_C_ 77.3 for both), suggesting that both C-7 and C-11 were oxygenated. These moieties together only accounted for five degrees of unsaturation, meaning one additional epoxy ring had to be formed to fulfill the molecular formula. Indeed, the uncommon NOESY correlation from Me-15 (δ_H_ 1.55, s) to Me-13 (δ_H_ 1.34, s) hinted the presence of an C4-O-C11 epoxy bridge (Figure 3). The relative configuration was determined by analysis of the NOESY correlations (Figure 3). The correlations between H-3 (δ_H_ 3.90, br. s), Me-15, and Me-14 suggested they directed to a same face (β), while the correlation from Me-15 to Me-13 defined their proximity in space, then 7-OH had to be β-oriented. In addition, the correlations of Me-12 (δ_H_ 0.94, s)/H-9, H-9/H-1, and H-1/H-2 (δ_H_ 3.35, d) indicated they directed to the opposite face. Thus, compound **10** featured an unusual cyclic ether (C4-O-C11), representing a new 6/6/6 tricyclic system of the eremophilanes.

**Figure 13:**
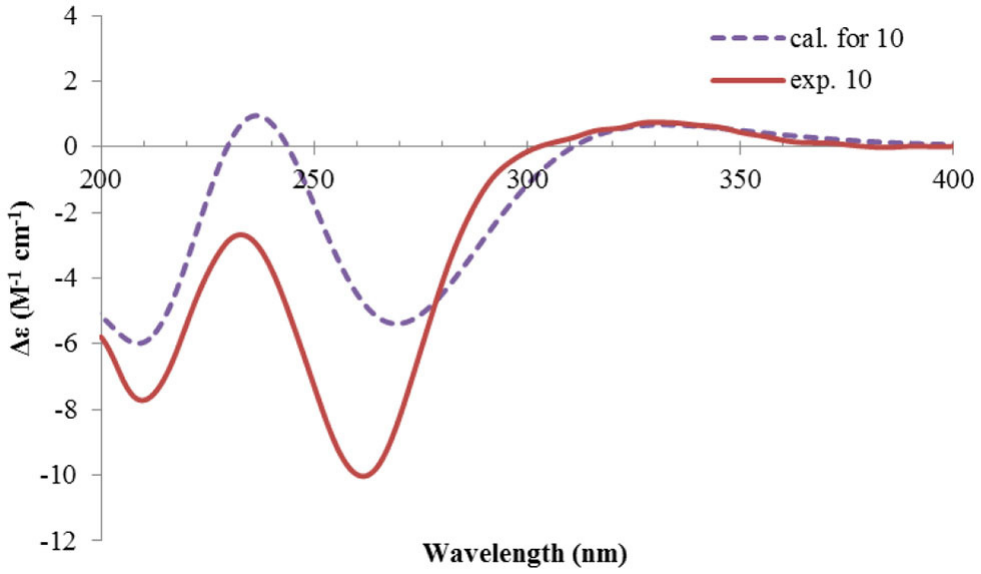
Experimental and calculated ECD spectra of **10**.

The absolute configuration of **10** was determined by ECD computations likewise. Only one major conformer (Figure 3) was found after geometry optimization, which was subjected to TDDFT ECD calculations using three different functions and basis set. All these calculations could reproduce the experimental spectrum (Supplementary Figure 14), among which the PBE0/TZVP-calculated spectrum gave the best fit (Figure 13). This allowed the elucidation of the 1*S*, 2*R*, 3*R*, 4*S*, 5*S*, 7*R* configuration. Meanwhile, compound **10** was converted to the 3-*O*-MPA esters (Supplementary Figure 15), and the absolute configuration of the secondary alcohol was successfully determined by the modified Mosher's method. By analysis of the Δδ (δ_R_-δ_S_) values around C-3 (Figure 8), the absolute configuration of C-3 was deduced as *R*. Therefore, its absolute configuration was confirmed independently.

**Figure 14:**
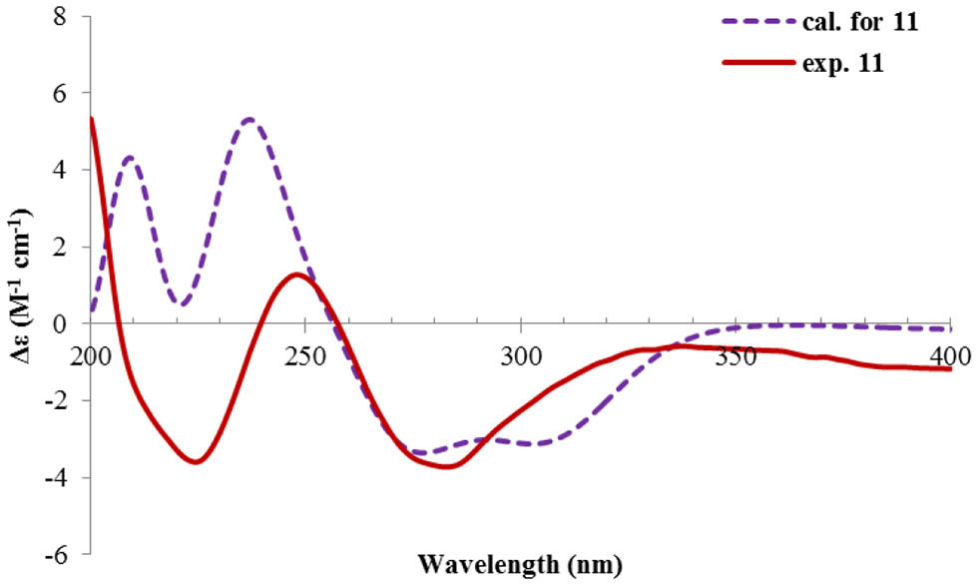
Experimental and calculated ECD spectra of **11**.

Rhizoperemophilane K (**11**) was isolated as a brown amorphous solid. Its molecular formula was deduced as C_15_H_14_O_5_ by HRESIMS. Unlike in **1**~**10**, this compound only has three methyl groups (δ_C_ 8.5, 29.1, 11.3; δ_H_ 1.97, 1.32, 2.07, each s); the other one was oxidized (δ_C_ 172.3, C) and incorporated into a lactone ring, as inferred from the NMR data (Tables 1, 3), hinting a lactone type of eremophilane. Its structure was closely related to 2-oxo-3-hydroxy-eremophila-1(10),3,7(11),8-tetraen-8,12-olide (**20**) (Qin et al., 2015); however, only one olefinic proton observed in **11**, and bearing one more oxygen atom in the molecular formula, indicated that one of the two olefinic protons in **20** was substituted by a hydroxyl group in **11**. Indeed, this substitution was at C-1, as HMBC correlations from H-9 (δ_H_ 6.68, s) to C-10 (δ_C_ 131.8, C), and C-1 (δ_C_ 146.4, C), were observed. Since neither ECD nor optical rotation data of the known structure (**20**) was available in literature, it was impossible to deduce the absolute configuration of **11** by comparison with **20**. So ECD calculation for **11** was performed to determine the absolute configuration. The calculated spectra fitted with the experimental data (Supplementary Figure 17), among which the BH&HLYP/TZVP-calculated one displayed the best match (Figure 14). Meanwhile, the optical rotation was calculated at the level of b3lyp/6-31+g(d,p) with PCM=MeOH, resulting in a theoretic [α]_D_ value of −496.01, which was comparable to the experimental data (${[\alpha ]}_{\text{D}}^{25}-$232.73 (*c* 0.11, MeOH)). Therefore, the absolute configuration of **11** was elucidated to be 5*R*, the same as in **20**.

**Figure 15:**
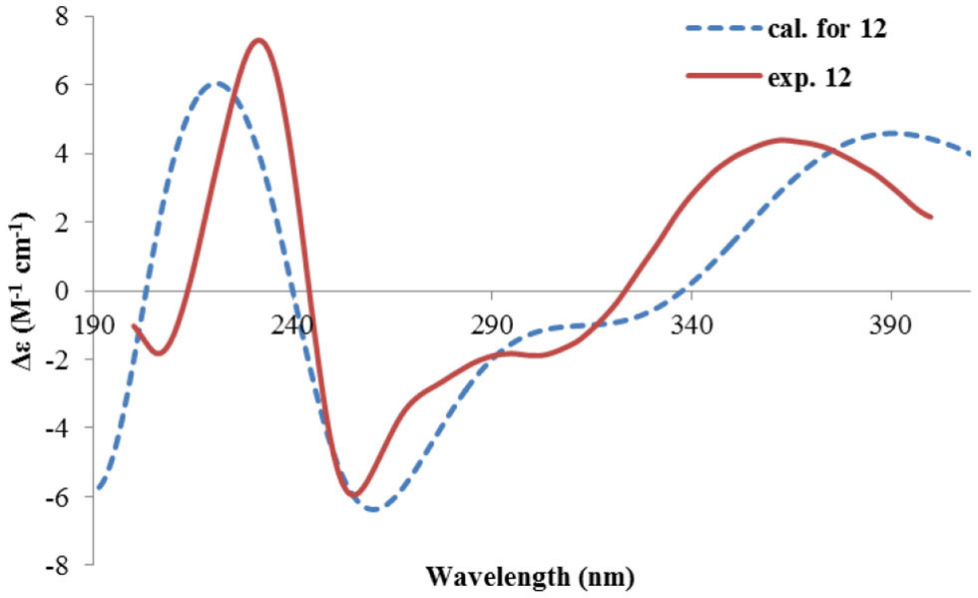
Experimental and calculated ECD spectra of **12**.

Rhizoperemophilane L (**12**) was isolated as a yellowish oil, with molecular formula as C_15_H_14_O_4_. This compound represented a furan type of eremophilane by the characteristic NMR data [CH-12: δ_H_ 7.77 (s)/δ_C_ 149.5; δ_C_ 124.2 (C-11), 142.4 (C-7), 148.0 (C-8); CH_3_-13: δ_H_ 2.09 (s)/δ_C_ 7.5]. Its NMR data were similar to 2,9-dioxoeuryopsin (Mei et al., 2001); however, compound **12** has two more quaternary sp^2^ carbons (δ_C_ 146.7, 136.0) but with one less methylene and methine group, compared to the latter compound. The HMBC correlations from the olefinic methyl group (δ_H_ 2.11, s, Me-15) to these sp^2^ carbons and C-5 suggested that one C=C bond existed between C-3/C-4 and C-3 (δ_C_ 146.7) was hydroxylated by taking into consideration the chemical shifts and the molecular mass. The absolute configuration was determined via ECD calculations (Figure 15). Moreover, the result indicated it possessed the 5*R* configuration (i.e., β-configurated 5-methyl group) as usual.

**Figure 16:**
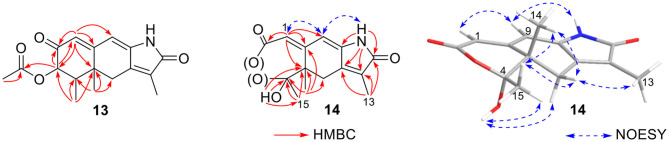
Selected HMBC correlations of **13** and **14**, and NOESY correlations of **14**.

**Figure 17:**
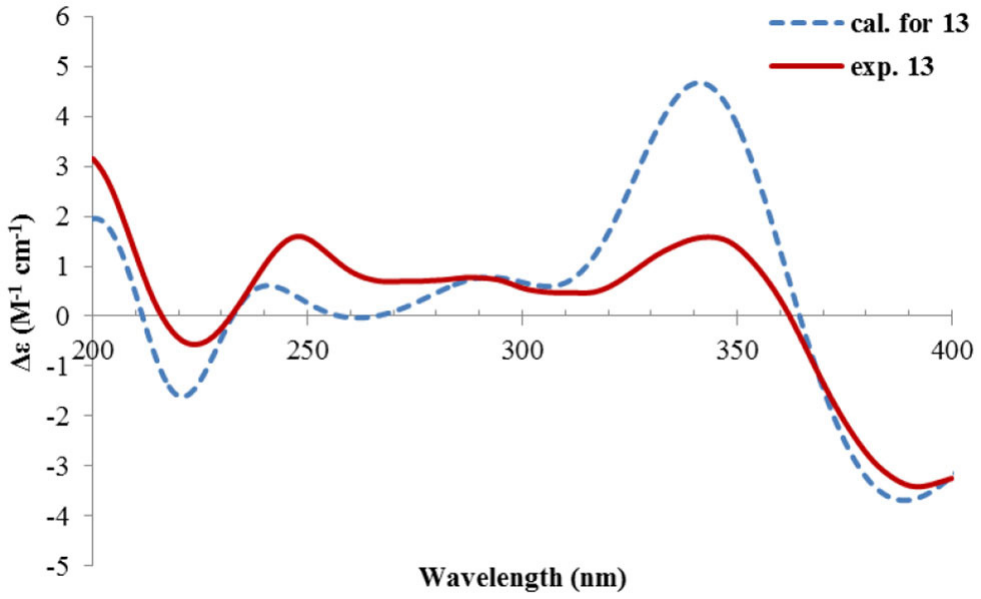
Experimental and calculated ECD spectra of **13**.

Rhizoperemophilane M (**13**) was isolated as a nitrogen-containing compound with a molecular formula of C_17_H_19_NO_4_ as determined by HRESIMS. This was reminiscent of a lactam-type of eremorphilane (Lin et al., 2014), and its NMR data were closely related to the co-isolated compound (**22**) (Lin et al., 2014), and the differences were ascribed to the western ring, in which a keto group (δ_C_ 192.6) of **13** replaced the methoxymethine group of **22**. This was confirmed by the HMBC correlations seen from H-1 (δ_H_ 6.04, s) and H-3 (δ_H_ 5.46, d) to the keto group (C-2) (Figure 16) and can also explain the obvious downfield shift of C-10 (δ_C_ 163.9) compared to that of **22**. The relative configuration of the chiral centers in **13** was determined to be the same as those of **22**, due to the similar ^3^*J* values and the NOESY correlations. ECD calculations (Figure 17 and Supplementary Figure 20) have confirmed its absolute configuration to be 3*R*, 4*R*, 5*R*, as expected.

Rhizoperemophilane N (**14**) was isolated as a light-yellowish amorphous solid, whose molecular formula was determined as C_14_H_15_NO_4_. It displayed a similar UV absorption profile to that of **13**, suggesting it to be a congener of the latter. The NMR data of **14** were similar to those of **13** for the cyclohexene (C5~C10) and the lactam part, indicating the same construction for these rings, which were confirmed by analysis of the HMBC and NOESY correlations (Figure 16). However, they differed in the western ring, in which the oxymethine (C-3) and acetyl groups of **13** were missing in **14**, while one dioxygenated quaternary carbon (δ_C_ 104.9) in **14** replaced that of a methine (C-4) in **13**. This quaternary carbon was located at C-4, by the characteristic HMBC correlations from both Me-14 (δ_H_ 1.11, s) and Me-15 (δ_H_ 1.52, s) to it. When measured in DMSO-*d*_6_, two D_2_O-exchangeable protons were seen, one resonating at δ_H_ 10.30 that showed HMBC correlations to the carbons of the lactam ring and the other one at δ_H_ 7.26 that showed HMBC correlations to C-4 (δ_C_ 104.9), Me-15 (δ_C_ 21.9), and C-5 (δ_C_ 41.6) (Figure 16). Obviously, the first one was the amide proton; the second one is the proton of the hydroxyl group attached to C-4. By considering the chemical shift of C-2 (δ_C_ 163.5), this carbon should belong to a carboxyl group and had to be connected to the dioxygenated carbon (C-4) *via* an ester bond to complete the structure of **1**, as required by the molecular formula. Thus, compound **14** has an unprecedented nor-eremorphilane skeleton, in which C-3 was missing.

**Figure 18:**
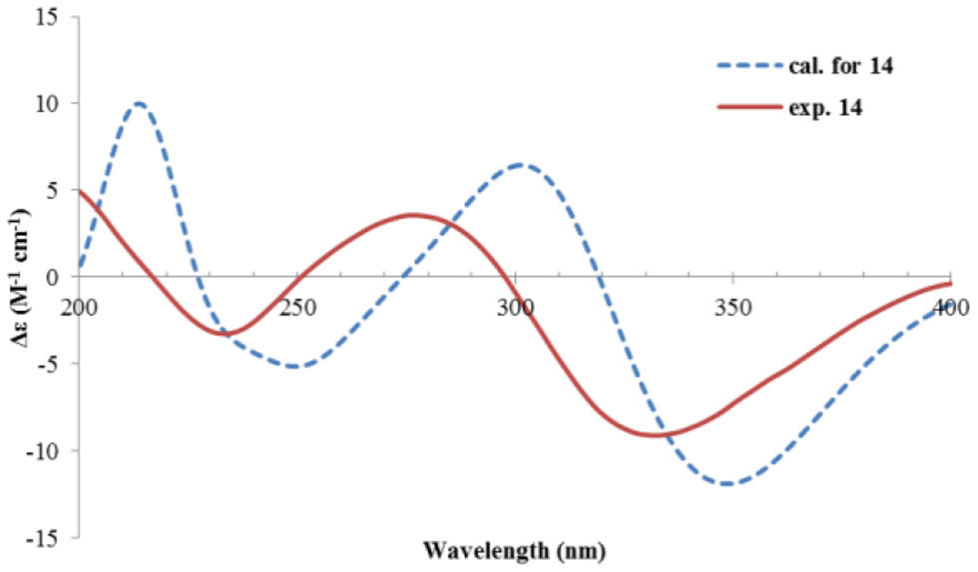
Experimental and calculated ECD spectra of **14**.

The relative configuration was determined by analysis of the NOESY spectrum (Figure 16). The correlation between Me-15 and Me-14 suggested they were co-faced (β), while 4-OH was oriented to the opposite. The absolute configuration of this compound was deduced by ECD calculations. As shown in Figure 18, the calculated spectrum of (4*R*, 5*S*)-**14** matched well with the experimental data.

The other isolated compounds were identified by comparing the spectroscopic data with the literature and included guignarderemophilane D (**15**) (Liu et al., 2015), 1α-hydroxyhydroisofukinon (**16**) (Bohlmann and Knoll, 1979), 2β-hydroxyl-1,7(11),9-eremorphilatrien-8-one (**17**) (Lin et al., 2014), guignarderemophilane B (**18**) (Liu et al., 2015), PR-toxin dimethyl acetal (**19**) (Darsih et al., 2015), 2-oxo-3-hydroxy-eremophila-1(10),3,7(11),8-tetraen-8,12-olide (**20**) (Qin et al., 2015), acremeremophilane N (**21**) (Cheng et al., 2016), and 2α-methoxyl-3β-acetoxyl-eremophila-1(10),7(11),8-trien-8,12-olactam (**22**) (Lin et al., 2014).

**Figure 19:**
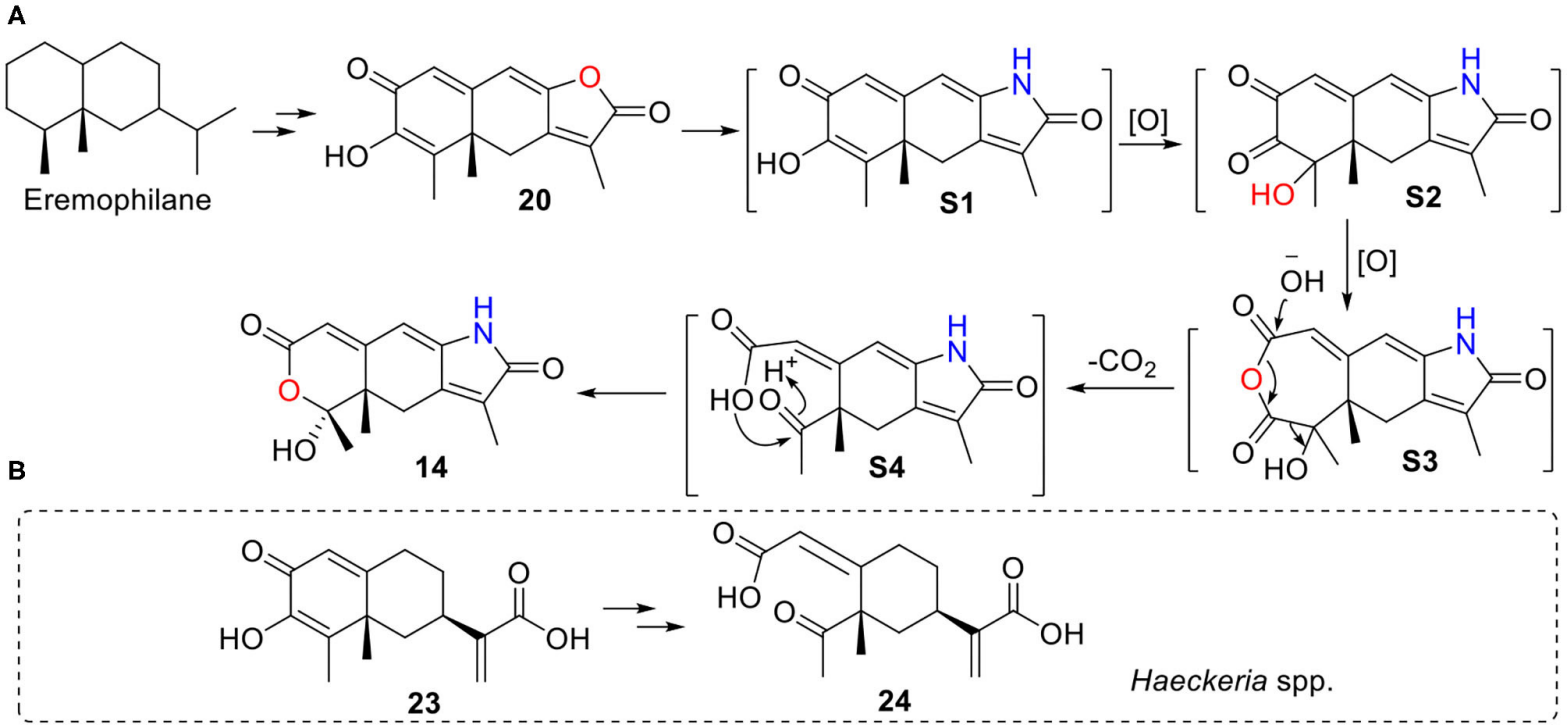
A plausible biosynthetic pathway for **14**. **(A)** Hypothetic pathway from **20** to **14**. **(B)** Similar pathway might occur from **23** to **24** in *Haeckeria* spp.

Rhizoperemophilane N (**14**) has an unusual 2,3-seco-3-nor-eremorphila-lactone skeleton. The plausible biosynthetic pathway of **14** was proposed based on the structure relationships between the isolated congeners as depicted in Figure 19. **S1** should be a key intermediate that could be derived from **20** by transamination, which was followed by two oxidative reactions, first at C-4, then Baeyer–Villiger oxidation to insert an oxygen atom between C-2/C-3, to form the anhydride intermediate **S3**. It is worth noting that **S1** has been reported from *Penicillium citreonigrum*, with the trivial name citreopenin (Yuan et al., 2015), though not being isolated in the present study. A decarboxylation could happen by the attack of water to **S3** resulting in a 2,3-seco-3-nor product (**S4**), which could be converted to **14** by ketalization. Interesting, a 2,3-seco-3-noreremorphilane derivative (**24**) and its hypothetic precursor (**23**) have been reported from the plants *Haeckeria* spp. (Zdero et al., 1991). Likely, a similar biosynthetic pathway was shared though in a different kingdom. It was not surprising that if those metabolites in the plants were actually the products of the endophytic fungi, though less than a dozen of compounds has been reported from both plants and fungi (Yuyama et al., 2017).

The isolated compounds were tested for their antibacterial activities against six bacterial pathogens *A. tumefaciens, B. subtilis, P. lachrymans, R. solanacearum, S. haemolyticus*, and *X. vesicatoria*, among which compounds **11**, **16**, and **20** displayed inhibitions with MIC values of 32~128 μg/mL, while the other tested compounds were inactive (MIC >128 μg/mL) (Table 4). Moreover, compound **16** showed the strongest inhibition to all the tested bacteria, while **11** and **20** only inhibited the growth of four and three bacteria, respectively, with the same MIC value of 128 μg/mL. The structure–activity relationship was unclear.

**Table 4:** Antibacterial activity.

**Compound**	**MIC/IC_**50**_ (μg/mL)^a^**	***tumefaciens***	***B. subtilis***	***P. lachrymans***	***R. solanacearum***	***S. haemolyticus***	***X. vesicatoria***
**11**	MIC	128	>128	128	128	>128	128
	IC_50_	60.55	nd^b^	53.68	55.26	nd	66.62
**16**	MIC	64	128	32	128	64	64
	IC_50_	20.91	37.80	14.06	36.98	27.31	24.04
**20**	MIC	128	>128	>128	128	>128	128
	IC_50_	46.81	Nd	nd	48.22	nd	53.10
Streptomycin sulfate^c^	MIC	16	16	16	16	16	16
	IC_50_	3.82	6.11	4.16	3.29	8.97	6.10

a *The other tested compounds were inactive (MIC >128 μg/mL)*.

b *nd: not determined*.

c *Positive control*.

The isolated compounds were also tested for their cytotoxic activities against five human cancer carcinomas (BGC823, Daoy, HCT116, HepG2, and NCI-H1650). Among them, nor-eremophilane **14** showed selective inhibition against the non-small-cell lung carcinoma cells (NCI-H1650) and gastric carcinoma cells (BGC823), with IC_50_ values of 15.8 and 48.2 μM, respectively. The other tested compounds were inactive with IC_50_ >50 μM. This was not unexpected, as the known compounds **15** (Liu et al., 2015), **17** (Lin et al., 2014), **18** (Liu et al., 2015), **20** (Qin et al., 2015), and **22** (Lin et al., 2014) were reported to be non-cytotoxic, while **19** displayed only weak cytotoxicity against the leukemia cells (Darsih et al., 2015).

As some eremophilanes were reported to be phytotoxic (Capasso et al., 1984; Bunkers et al., 1990; Del Valle et al., 2015), the isolated compounds were screened for their phytotoxicities against rice seedlings as reported previously in our lab (Lu et al., 2015; Sun et al., 2017). Compounds **5**, **6**, **12**, **13**, **16**, and **19** were found to exhibit strong inhibition against the radicle elongation of rice seedlings, while the other tested compounds did not at the tested concentrations (Table 5). All the active compounds showed more than 50% inhibition at 400 μg/mL, and compounds **6**, **16**, and **19** displayed such effect at a lower concentration of 200 μg/mL, while for **13**, an even lower concentration of 100 μg/mL was recorded. As for the structure–activity relationship, it seems that the polarity of the molecule might play a role in the phytotoxicity. Among the tested substances with a 2,2-dimethylethylenyl group as in **1**, **2**, **4**, and **16**, only **16** that with one hydroxyl group in the western ring was active, while the dihydroxylated product **1**, **2**, or **4** (more polar for having one additional double bond at C-1/2) was inactive. This generality could expand to include those 7-hydroxylated compounds, if one considered the 7-hydroxyl group exerting a similar effect to the polarity as that of the 7,11-double bond. Indeed, compounds **5** and **6** that only have one oxygenation in the western ring were phytotoxic, while the dihydroxylated analog **8**, or the more polar compound **10** did not display any inhibition. A similar relationship was observed using the leaf puncture wound assay by Bunkers and coworkers (Bunkers et al., 1990), albeit different eremorphilanes were tested. However, when it comes to the 12-oxygenated compounds, the structure–activity relationship seems elusive. For instance, the lactam-type metabolite **13** exhibited strong activity, while its lactone counterpart **21** did not. Compounds **12** and **20** were constitutional isomers, but only the former one was active. PR toxin was a notorious mycotoxin and also a phytotoxic substance that strongly inhibited the growth of the tomato seedlings (Capasso et al., 1984). In this study, we found that its dimethyl acetal (**19**) was also a potent inhibitor, implying that the aldehyde group was not necessary for the toxicity. This was consistent with the finding on the phomenone derivatives (Capasso et al., 1984; Bunkers et al., 1990).

**Table 5:** Inhibitory activities against the radicle elongation of rice seeds.

**Compound^a^**	**Inhibitory rate (%)^b^ at tested concentration (μg/mL) of**			
	**50**	**100**	**200**	**400**
**5**	6.3 ± 7.4 vw	11.9 ± 5.8 tu	32.9 ± 4.8 no	50.0 ± 4.1 ij
**6**	10.3 ± 4.1 uv	42.5 ± 9.4 klm	75.9 ± 5.7 cde	86.2 ± 4.9 a
**12**	4.6 ± 7.8 vw	18.1 ± 5.7 rst	36.8 ± 8.4 mn	65.5 ± 9.4 fg
**13**	22.7 ± 7.7 pqrs	52.6 ± 7.0 hi	79.4 ± 6.7 bcd	80.7 ± 2.9 bc
**16**	nd *^c^* w	29.9 ± 7.2 op	62.9 ± 7.8 g	72.2 ± 4.4 de
**19**	22.7 ± 10.0 qrs	44.0 ± 8.7 jkl	57.2 ± 6.2 h	78.7 ± 8.5 bcd
Glyphosate^d^	70.8 ± 8.3 ef	84.2 ± 8.3 ab	84.8 ± 6.3 ab	90.1 ± 4.8 a

a *The other tested compounds did not show any inhibitory activity at the test concentrations*.

b *Each value represents the means of triplicate ± standard deviations. Different letters indicated significant differences among treatments including different compounds and their concentrations at p ≤ 0.05*.

c *nd: not detected*.

d *Positive control*.

## Conclusion

In summary, twenty-two eremorphilane-type sesquiterpenoids (**1**~**22**), including fourteen new structures (**1**~**14**), were isolated from the endophytic fungus *Rhizopycnis vagum* Nitaf22. Rhizoperemophilane J (**10**) has an unusual C-4/C-11 epoxy structure, and rhizoperemophilane N (**14**) features an unprecedented 2,3-seco-3-nor-eremophilane-2,4-olide skeleton, for which a plausible biosynthetic pathway was proposed. The structures of the new compounds were elucidated mainly by HRMS, NMR, and ECD, with the absolute configuration assignments by quantum chemical ECD calculations, the modified Mosher's method, and optical rotation calculations. These metabolites were evaluated for the antibacterial, cytotoxic, and phytotoxic activities. The results revealed that compounds **11**, **16**, and **20** were antibacterial, while **14** was selectively cytotoxic to the NCI-H1650 and BGC823 cell lines. Moreover, these eremophilanes were phytotoxic against the radicle growth of rice seedlings. And a possible structure–activity–relationship was discussed.

## Data Availability Statement

The raw data supporting the conclusions of this article will be made available by the authors, without undue reservation.

## Author Contributions

LZ, DL, and AW conceived and designed the experiments. AW was responsible for the isolation of compounds. DL and AW elucidated the structures. JD tested the cytotoxicity of the compounds. AW, RY, ZZ, and GG performed the experiments of antimicrobial and phytotoxic activities. DL, LZ, and AW interpreted the data and wrote the paper. JD revised the manuscript. All authors read and approved the final manuscript.

## Conflict of Interest

The authors declare that the research was conducted in the absence of any commercial or financial relationships that could be construed as a potential conflict of interest.
