# Supplementary material for: Eremophilane-Type Sesquiterpenoids From the Endophytic Fungus Rhizopycnis vagum and Their Antibacterial, Cytotoxic, and Phytotoxic Activities
Source: Front Chem. 2020 Oct 26;8:596889. doi: 10.3389/fchem.2020.596889 (PMC7649393; doi:10.3389/fchem.2020.596889)
Supplement: Supplementary file 1 [file Data_Sheet_1.PDF]

Supporting Information

Eremophilane-type Sesquiterpenoids from the Endophytic Fungus *Rhizopycnis vagum* and Their Antibacterial, Cytotoxic, and Phytotoxic Activities

Ali Wang[1], Ruya Yin[1], Zhiyao Zhou[1], Gan Gu[1], Jungui Dai[2], Daowan Lai[1] and Ligang Zhou[1]**

[1] Department of Plant Pathology, College of Plant Protection, China Agricultural University, Beijing 100193, China, [2] State Key Laboratory of Bioactive Substance and Function of Natural Medicines, Institute of Materia Medica, Chinese Academy of Medical Science & Peking Union Medical College, Beijing 100050, China

[*] Corresponding Authors

Daowan Lai: dwlai@cau.edu.cn; Ligang Zhou: lgzhou@cau.edu.cn

Contents

Figure S1. Optimized geometries of the predominant conformers of **1** at the B3LYP/6-31G(d) level in the gas phase. S3
Figure S2. Optimized geometries of the predominant conformers of **2** at the B3LYP/6-31G(d) level in the gas phase. S3
Figure S3. Optimized geometries of the predominant conformers of **4** at the B3LYP/6-31G(d) level in the gas phase. S3
Figure S4. The experiment ECD and the calculated spectra of **4** using three different methods. .. S4
Figure S5. Optimized geometries of the predominant conformers of **5** at the B3LYP/6-31G(d) level in the gas phase. S4
Figure S6. Comparison of the ^{1}H NMR spectra of the (*R*/*S*)-MPA esters of **5**. S5
Figure S6-1. Comparison of the ^{1}H NMR spectra of the (*R*/*S*)-MPA esters of **5** (Expansion). S5
Figure S7. Optimized geometries of the predominant conformers of **6** at the B3LYP/6-31G(d) level in the gas phase. S6
Figure S8. The experiment ECD and the calculated spectra of **6** using three different methods. .. S6
Figure S9. Optimized geometries of the predominant conformers of **7** at the B3LYP/6-31G(d) level in the gas phase. S7
Figure S10. Optimized geometries of the predominant conformers of **8** at the B3LYP/6-31G(d) level in the gas phase. S8
Figure S11. The experiment ECD and the calculated spectra of **8** using three different methods. S9
Figure S12. Optimized geometries of the predominant conformers of **9** at the B3LYP/6-31G(d) level in the gas phase. S9
Figure S13. Optimized geometries of the predominant conformers of **10** at the B3LYP/6-31G(d) level in the gas phase. S9
Figure S14. The experiment ECD and the calculated spectra of **10** using three different methods. S10
Figure S15. Comparison of the ^{1}H NMR spectra of the (*R*/*S*)-MPA esters of **10**. S10
Figure S15-1. Comparison of the ^{1}H NMR spectra of the (*R*/*S*)-MPA esters of **10** (Expansion). ...S11
Figure S16. Optimized geometries of the predominant conformers of **11** at the B3LYP/6-31G(d) level in the gas phase. S11
Figure S17. The experiment ECD and the calculated spectra of **11** using three different methods. S12
Figure S18. Optimized geometries of the predominant conformers of **12** at the B3LYP/6-31G(d) level in the gas phase. S12
Figure S19. Optimized geometries of the predominant conformers of **13** at the B3LYP/6-31G(d) level in the gas phase. S12
Figure S20. The experiment ECD and the calculated spectra of **13** using three different methods. S13
Figure S21. Optimized geometries of the predominant conformers of **14** at the B3LYP/6-31G(d) level in the gas phase. S13
NMR, HRMS spectra of the compounds S14

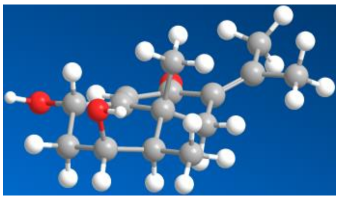

1-a, 5.8%

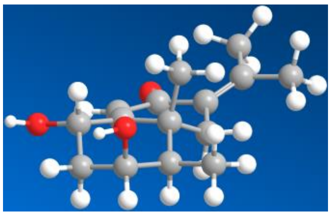

1-b, 4.0%

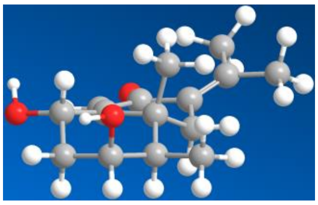

1-c, 29.5%

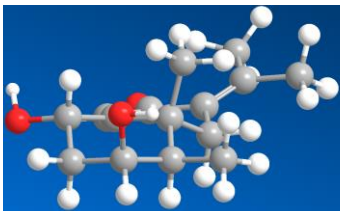

1-d, 18.1%

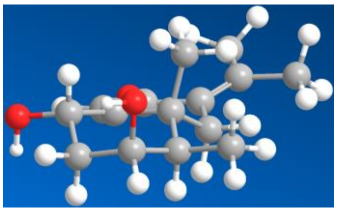

1-e, 26.0%

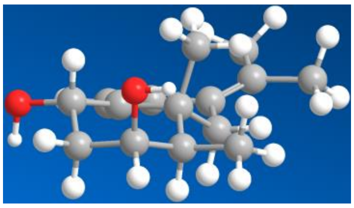

1-f, 16.3%

Figure S1. Optimized geometries of the predominant conformers of **1** at the B3LYP/6-31G(d) level in the gas phase.

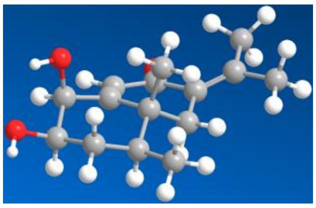

2-a, 12.3%

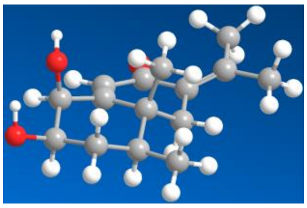

2-b, 76.4%

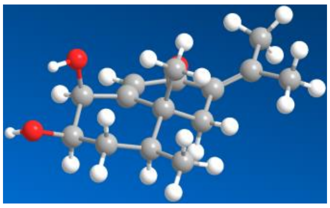

2-c, 11.0%

Figure S2. Optimized geometries of the predominant conformers of **2** at the B3LYP/6-31G(d) level in the gas phase.

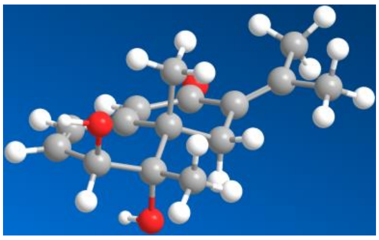

4-a, 36.9%

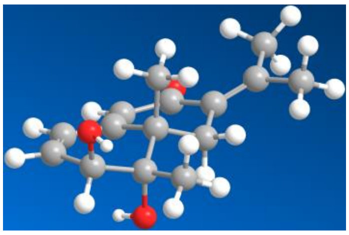

4-b, 49.8%

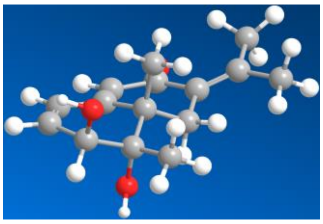

4-c, 5.9%

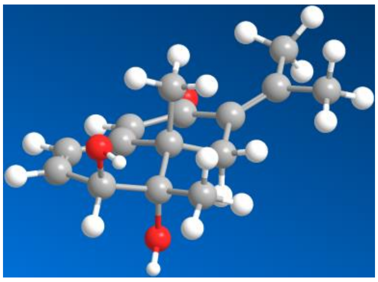

4-d, 2.1%

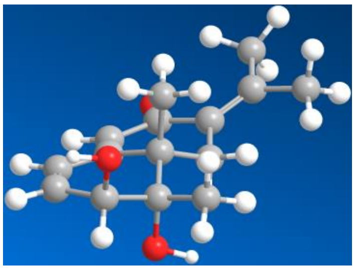

4-e, 3.4%

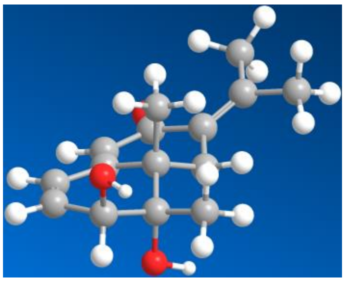

4-f, 1.9%

Figure S3. Optimized geometries of the predominant conformers of **4** at the B3LYP/6-31G(d) level in the gas phase.

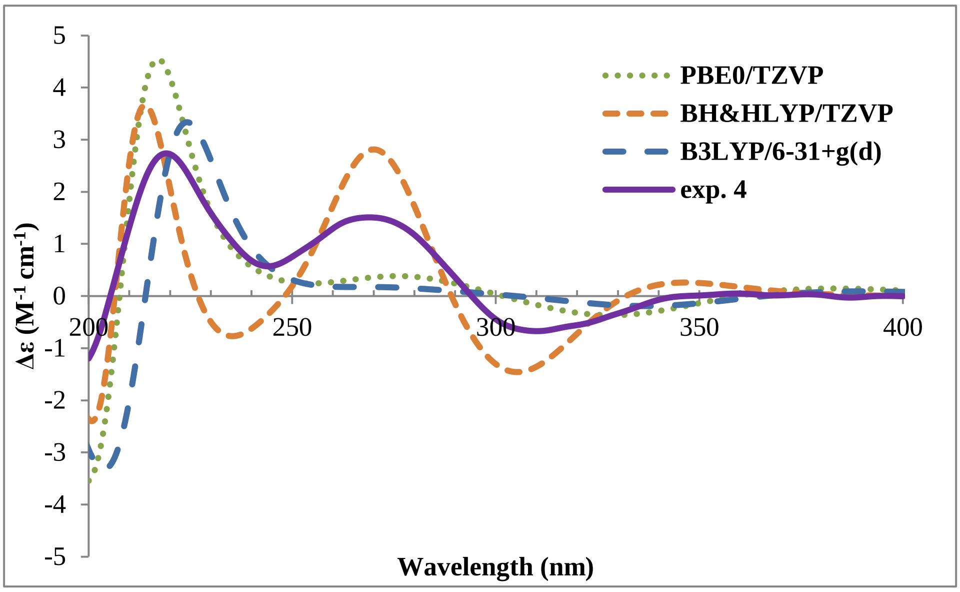

Figure S4. The experiment ECD and the calculated spectra of **4** using three different methods.

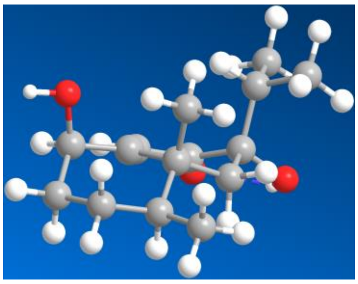

5-a, 9.9%

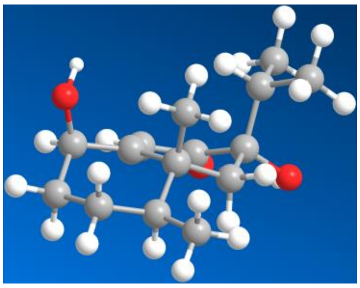

5-b, 90.1%

Figure S5. Optimized geometries of the predominant conformers of **5** at the B3LYP/6-31G(d) level in the gas phase.

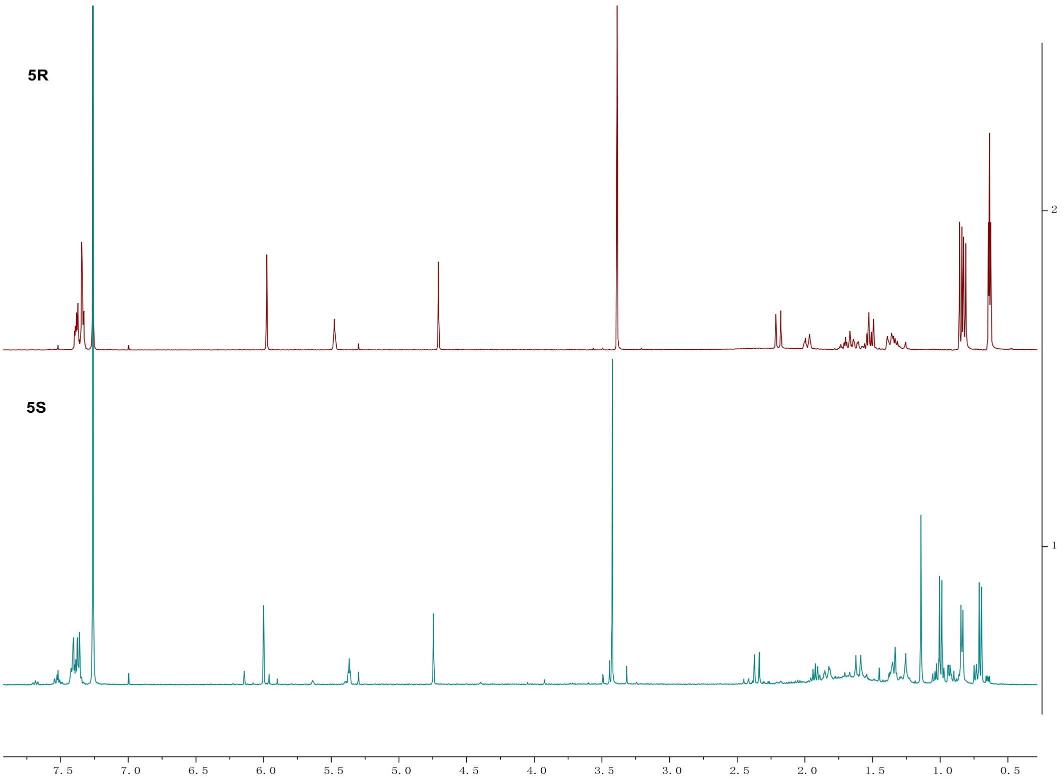

Figure S6. Comparison of the ^{1}H NMR spectra of the (*R*/*S*)-MPA esters of **5**.

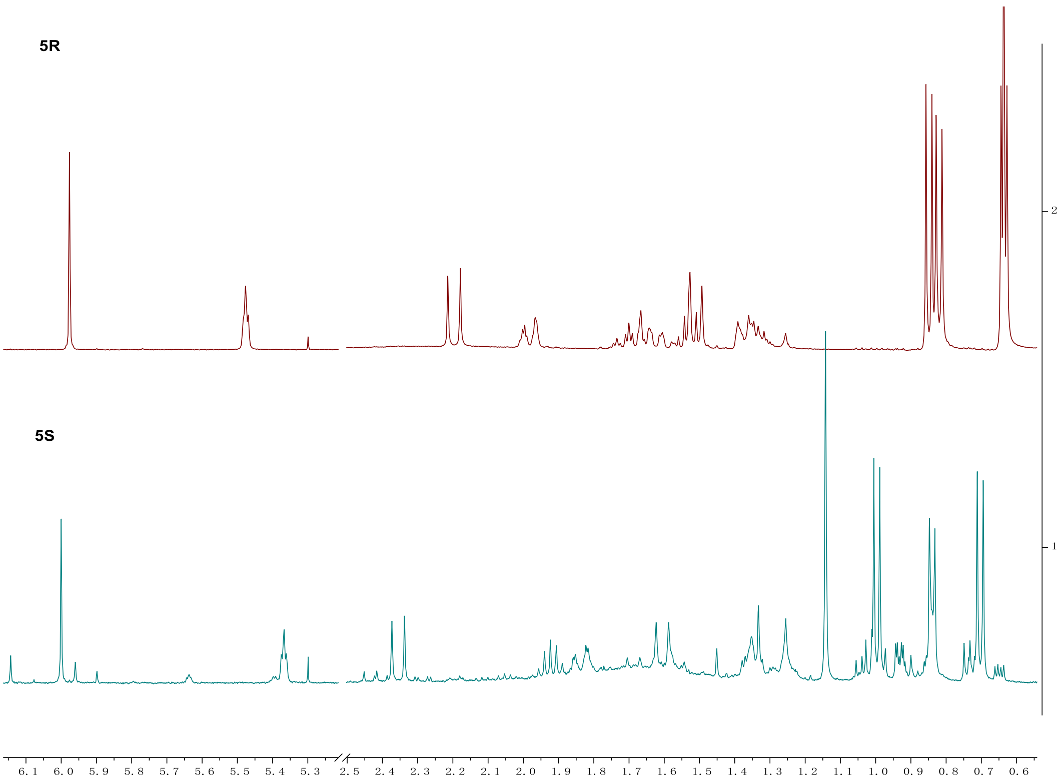

Figure S6-1. Comparison of the ^{1}H NMR spectra of the (*R*/*S*)-MPA esters of **5** (Expansion).

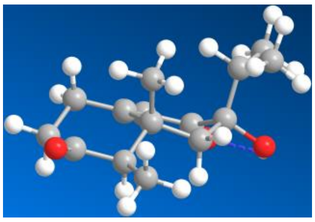

6-a, 75.2%

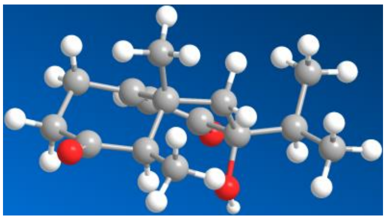

6-b, 23.4%

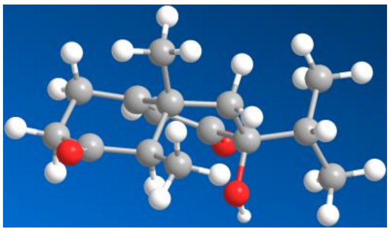

6-c, 1.1%

Figure S7. Optimized geometries of the predominant conformers of **6** at the B3LYP/6-31G(d) level in the gas phase.

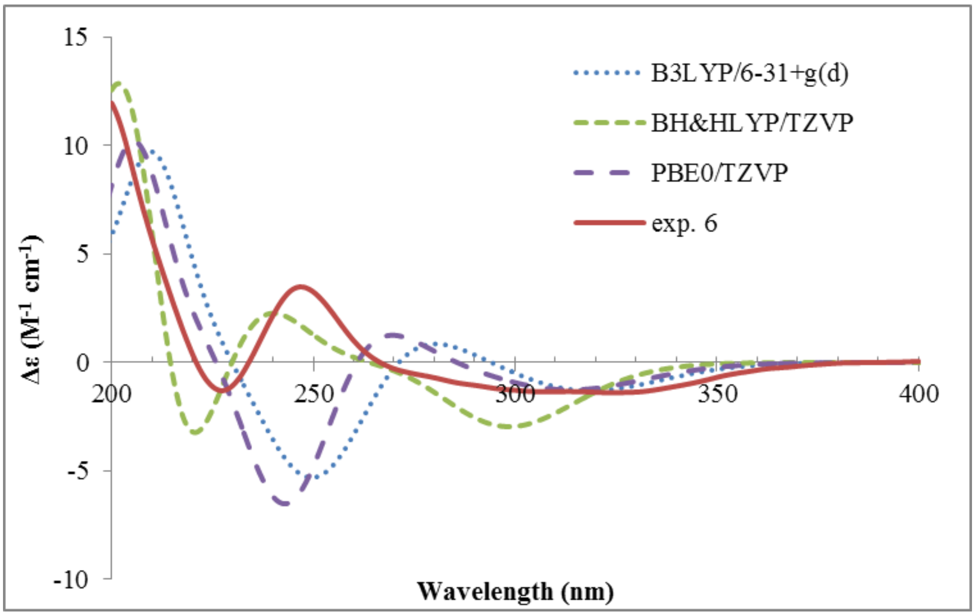

Figure S8. The experiment ECD and the calculated spectra of **6** using three different methods.

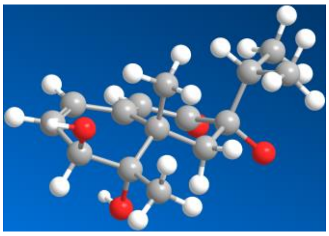

7-a, 12.0%

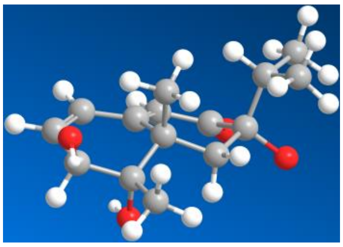

7-b, 32.4%

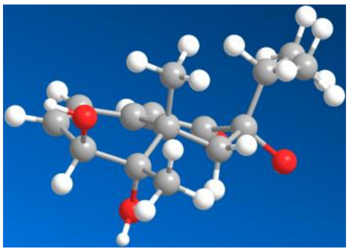

7-c, 2.6%

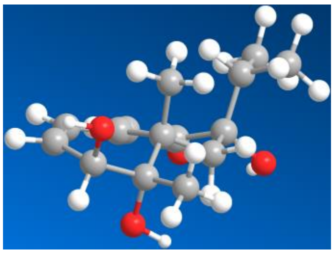

7-d, 2.4%

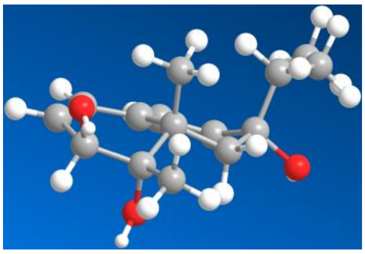

7-e, 2.1%

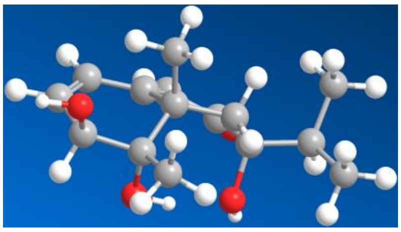

7-f, 11.5%

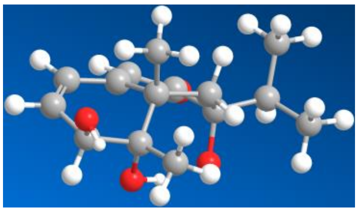

7-g, 32.4%

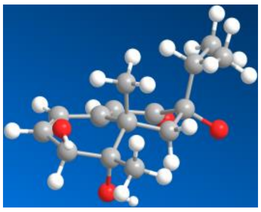

7-h, 4.3%

Figure S9. Optimized geometries of the predominant conformers of **7** at the B3LYP/6-31G(d) level in the gas phase.

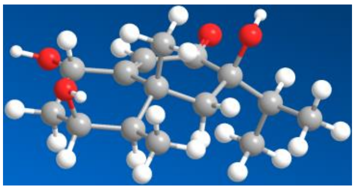

8-a, 7.3%

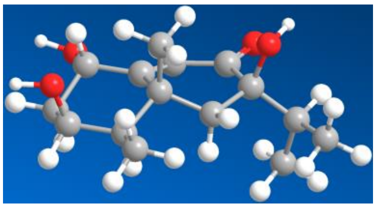

8-b, 3.7%

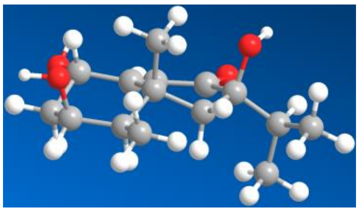

8-c, 19.7%

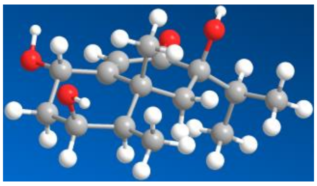

8-d, 20.4%

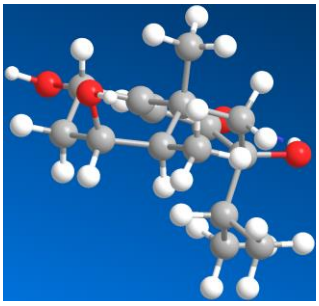

8-e, 2.2%

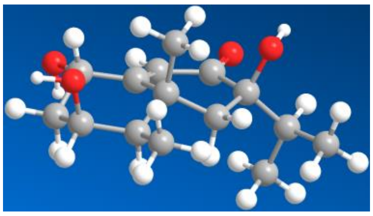

8-g, 17.5%

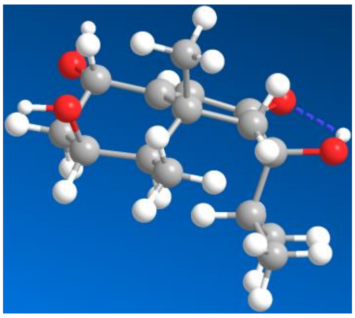

8-h, 4.4%

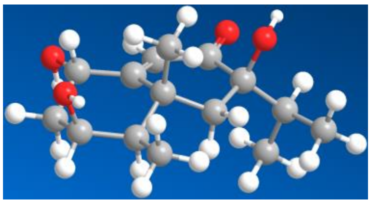

8-j, 15.7%

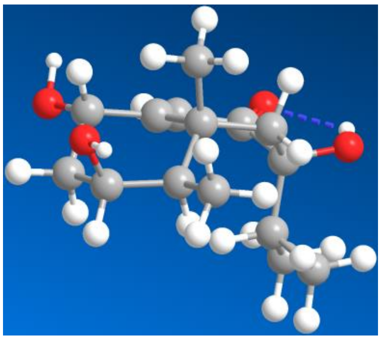

8-k, 4.6%

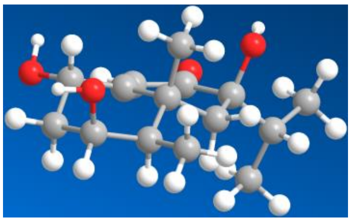

8-n, 1.3%

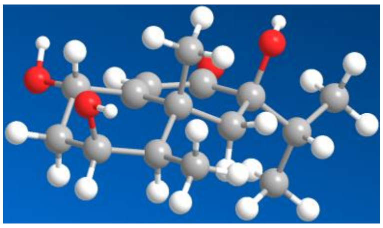

8-p, 1.2%

Figure S10. Optimized geometries of the predominant conformers of **8** at the B3LYP/6-31G(d) level in the gas phase.

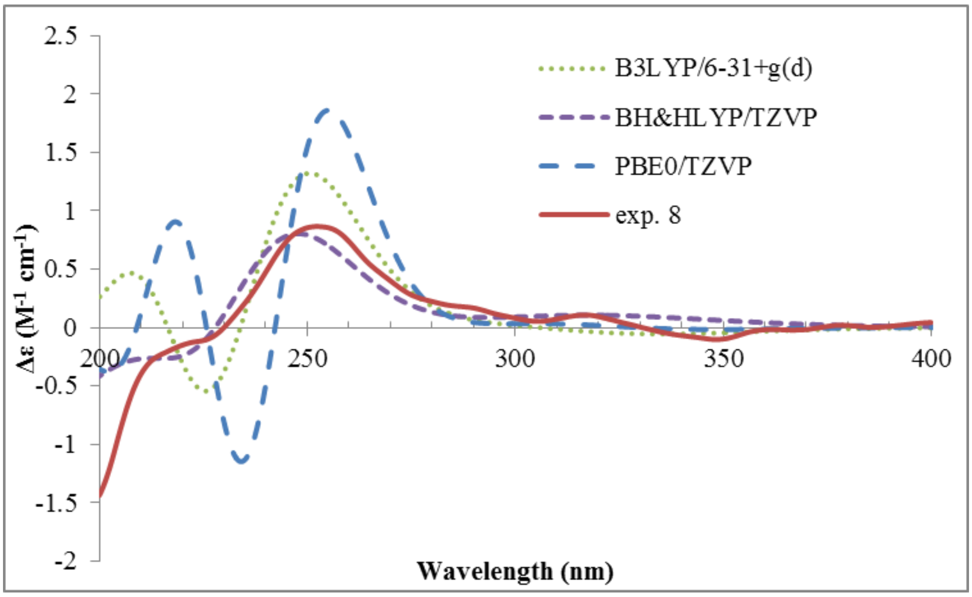

Figure S11. The experiment ECD and the calculated spectra of **8** using three different methods.

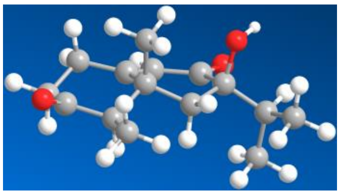

9-a, 58.9%

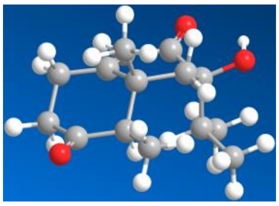

9-b, 20.4%

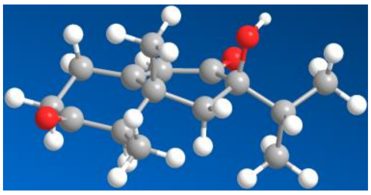

9-c, 3.6%

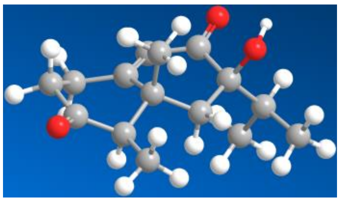

9-e, 13.8%

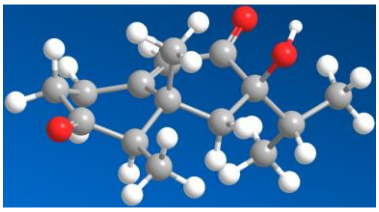

9-f, 1.5%

Figure S12. Optimized geometries of the predominant conformers of **9** at the B3LYP/6-31G(d) level in the gas phase.

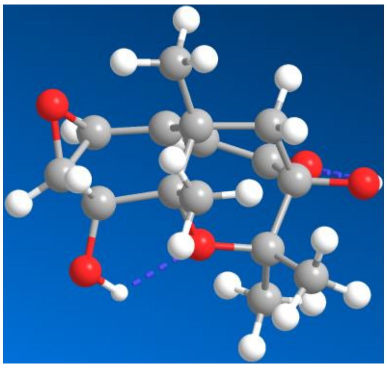

10-a, 100%

Figure S13. Optimized geometries of the predominant conformers of **10** at the B3LYP/6-31G(d) level in the gas phase.

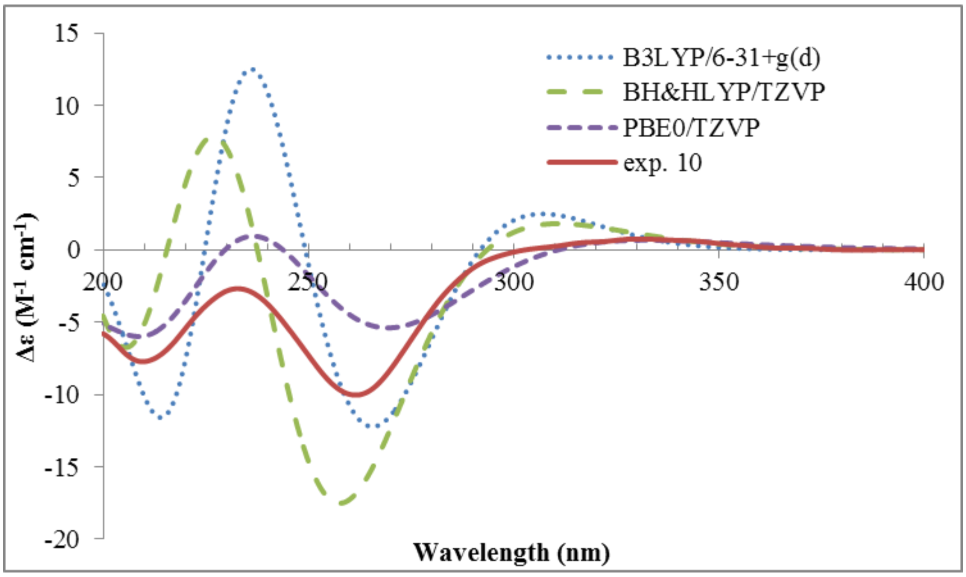

Figure S14. The experiment ECD and the calculated spectra of **10** using three different methods.

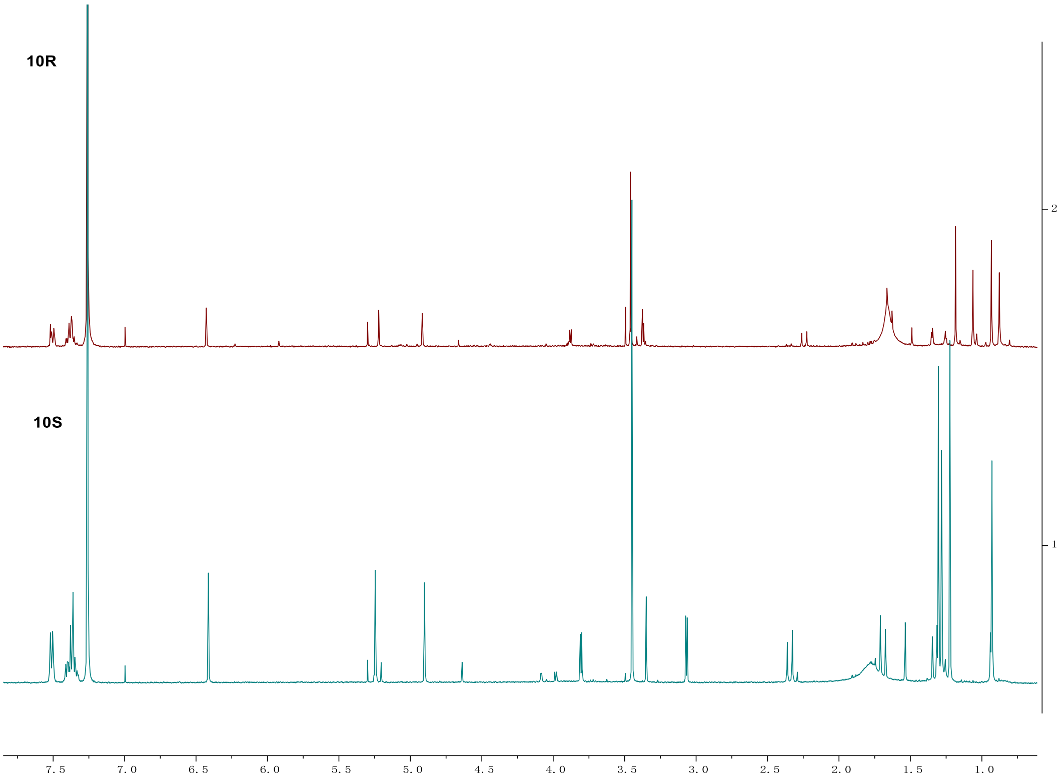

Figure S15. Comparison of the ^{1}H NMR spectra of the (*R*/*S*)-MPA esters of **10**.

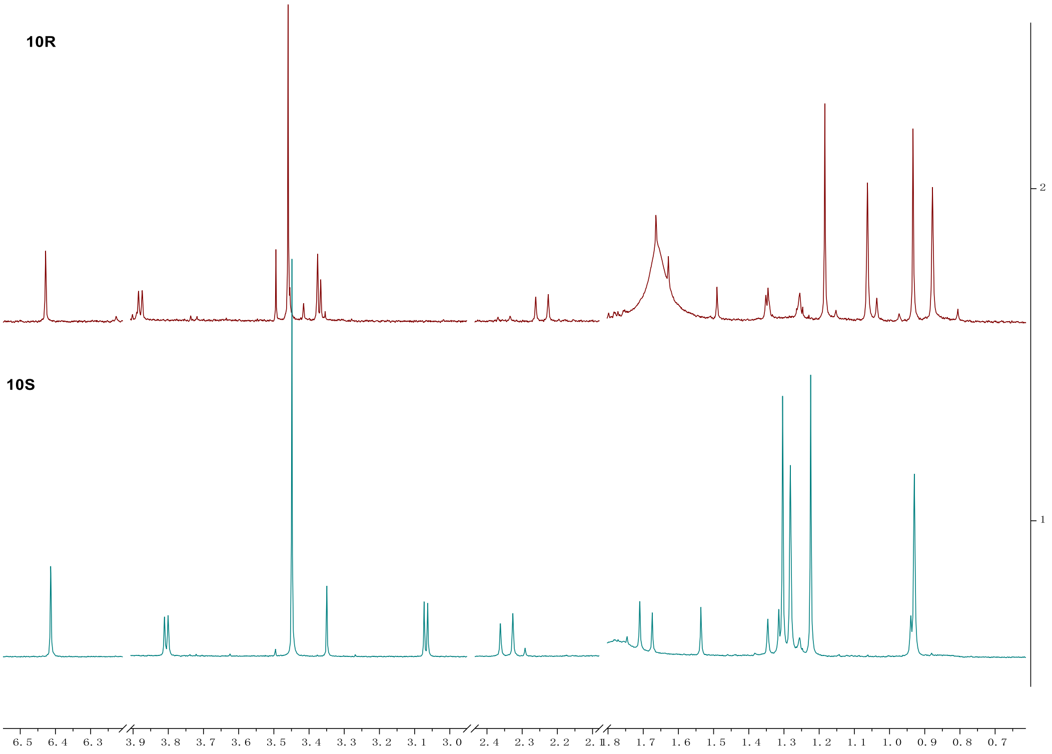

Figure S15-1. Comparison of the ^{1}H NMR spectra of the (*R*/*S*)-MPA esters of **10** (Expansion).

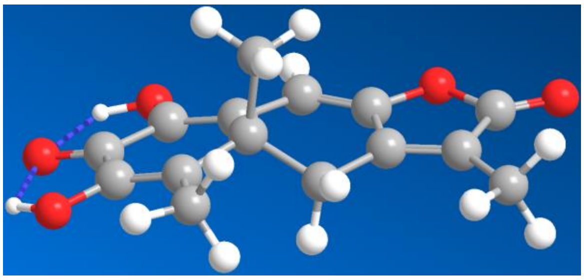

11-a, 100%.

Figure S16. Optimized geometries of the predominant conformers of **11** at the B3LYP/6-31G(d) level in the gas phase.

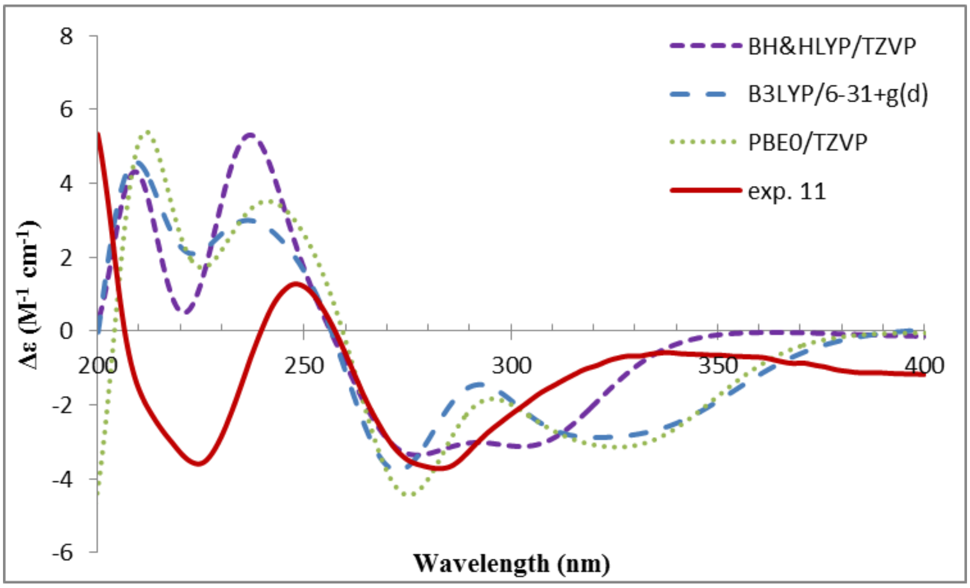

Figure S17. The experiment ECD and the calculated spectra of **11** using three different methods.

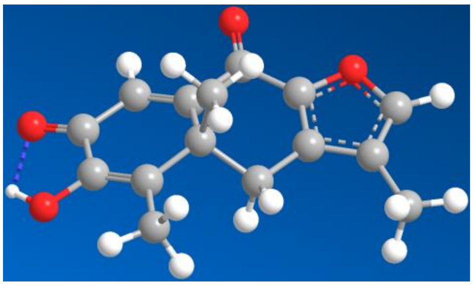

12-a, 100%

Figure S18. Optimized geometries of the predominant conformers of **12** at the B3LYP/6-31G(d) level in the gas phase.

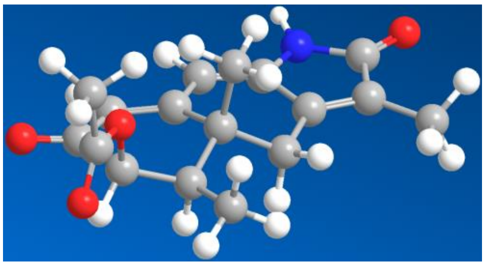

13-a, 100%

Figure S19. Optimized geometries of the predominant conformers of **13** at the B3LYP/6-31G(d) level in the gas phase.

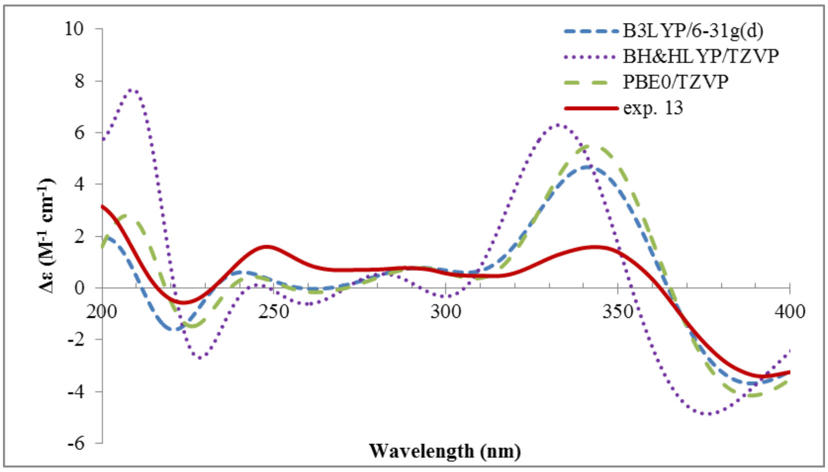

Figure S20. The experiment ECD and the calculated spectra of **13** using three different methods.

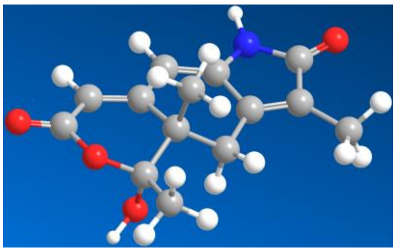

14-a, 85.3%

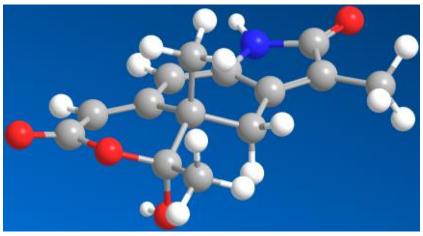

14-b, 14.7%

Figure S21. Optimized geometries of the predominant conformers of **14** at the B3LYP/6-31G(d) level in the gas phase.

NMR, HRMS spectra of the compounds

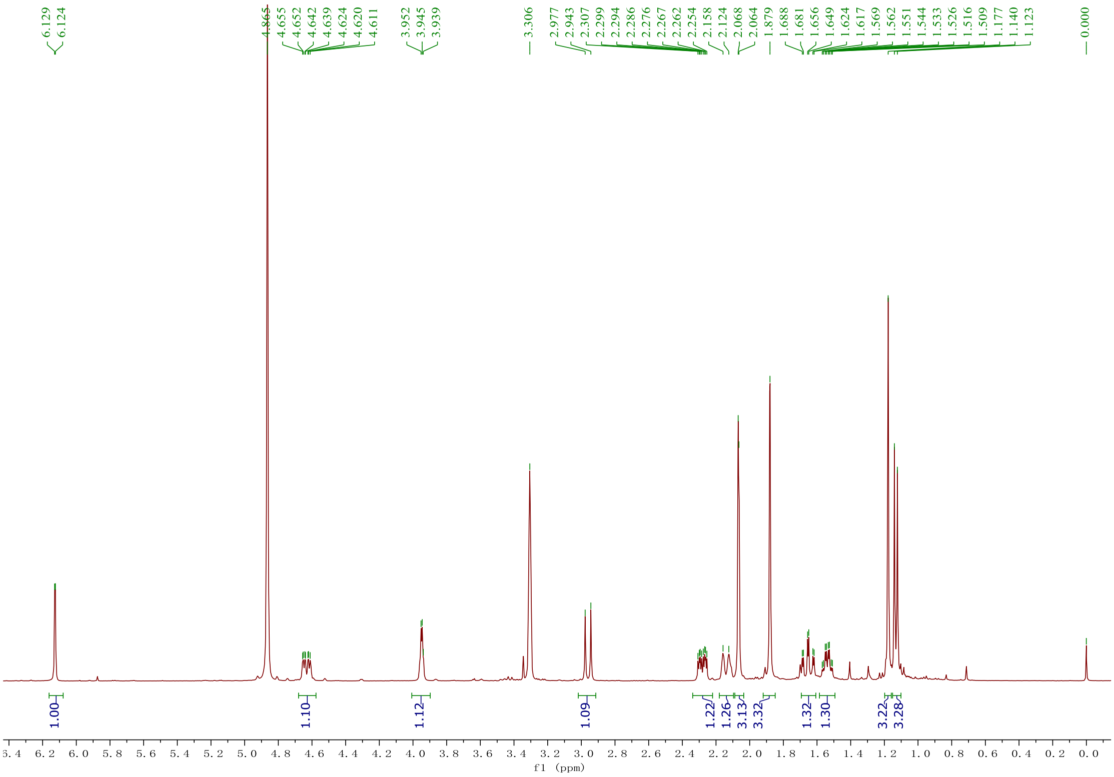

^{1}H NMR spectrum of **1**

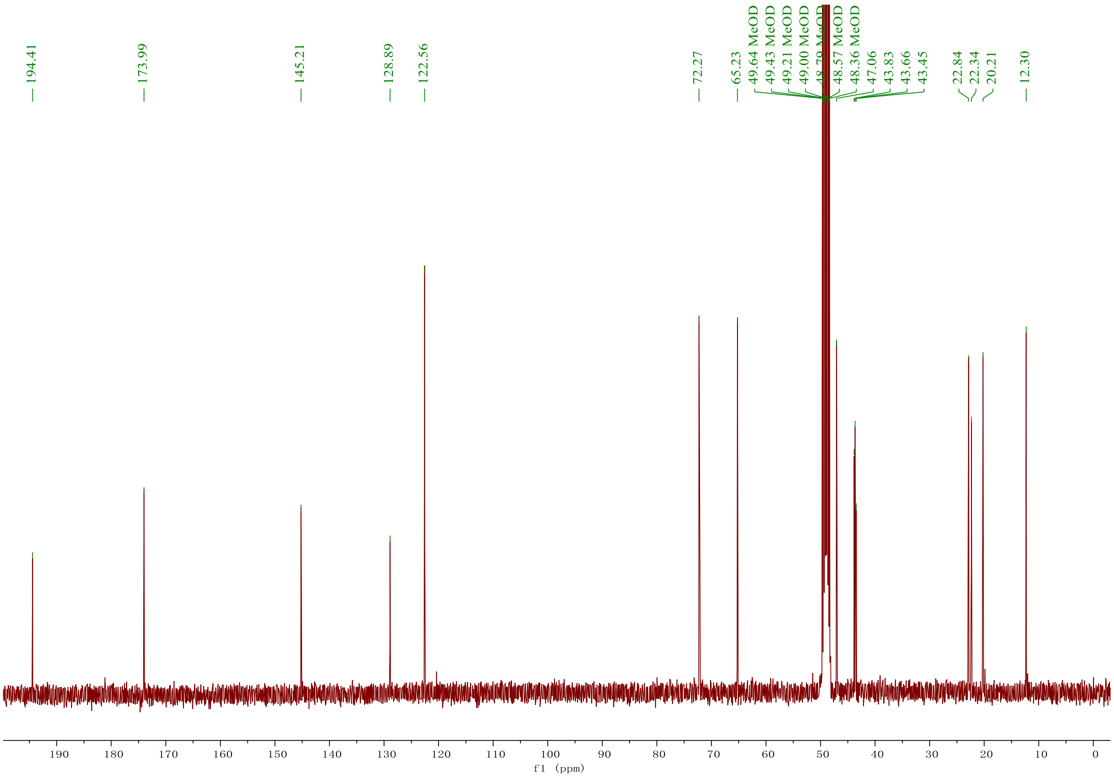

^{13}C NMR spectrum of **1**

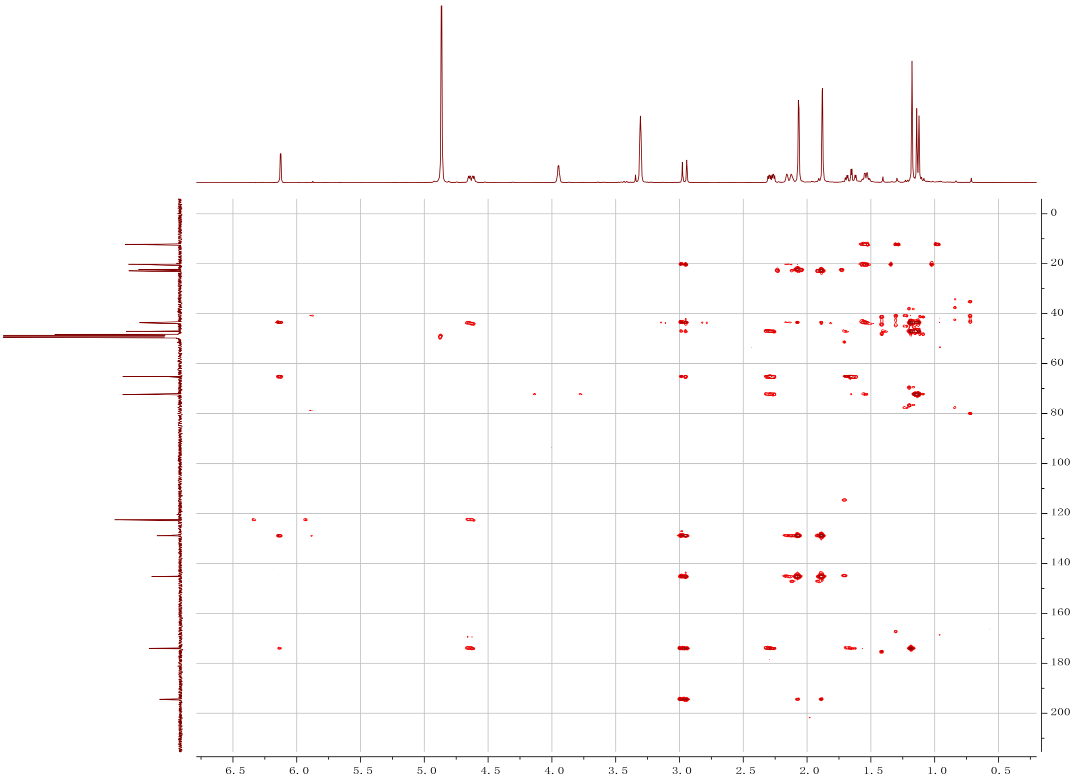

HMBC spectrum of **1**

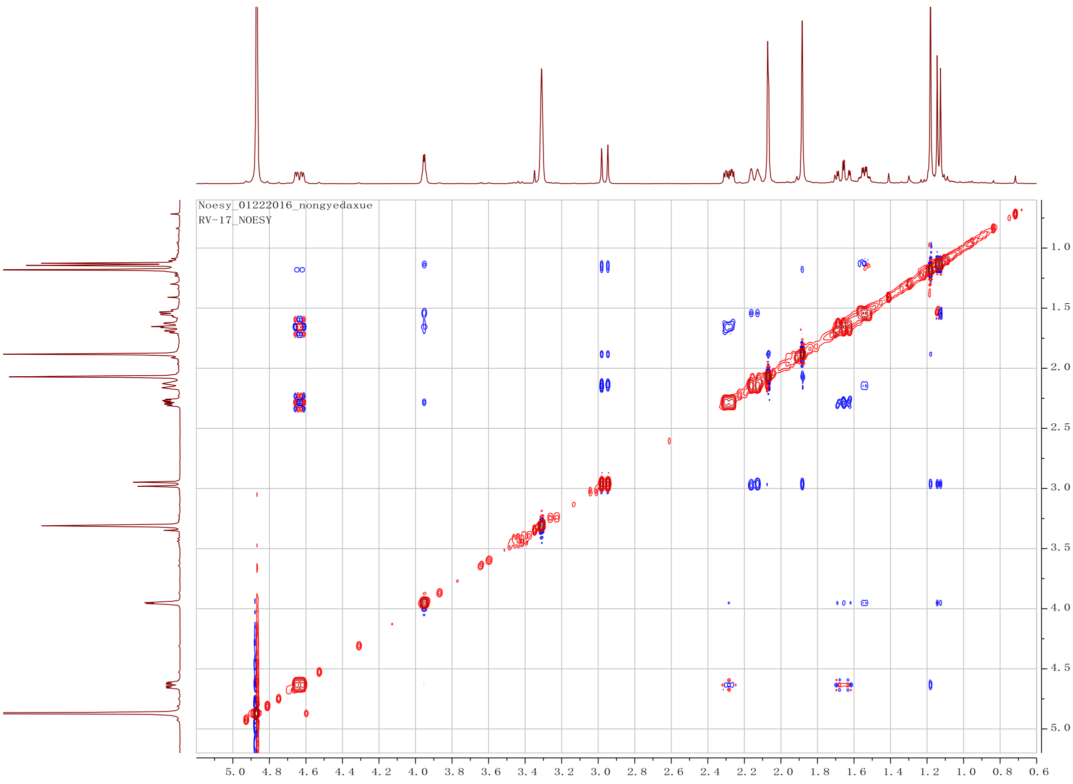

NOESY spectrum of **1**

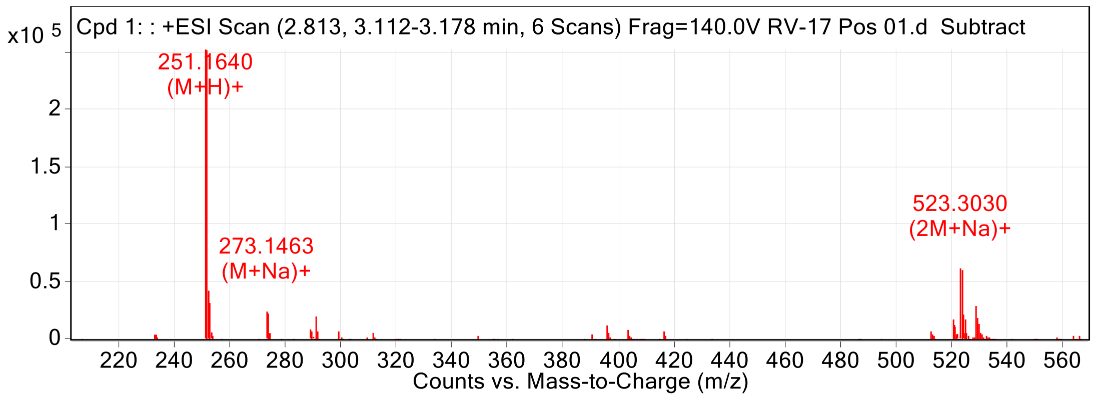

m/z	*Calc m/z*	Diff(ppm)	z	Abund	Formula	Ion
251.1640	251.1642	-0.8	1	252280.2	$C_{15}H_{23}O_3$	$(M+H)^+$
273.1463	273.1461	0.51	1	23876.4	$C_{15}H_{22}NaO_3$	$(M+Na)^+$
523.3030	523.303	0.01	1	61058.6	$C_{30}H_{44}NaO_6$	$(2M+Na)^+$

HRMS spectrum of **1**

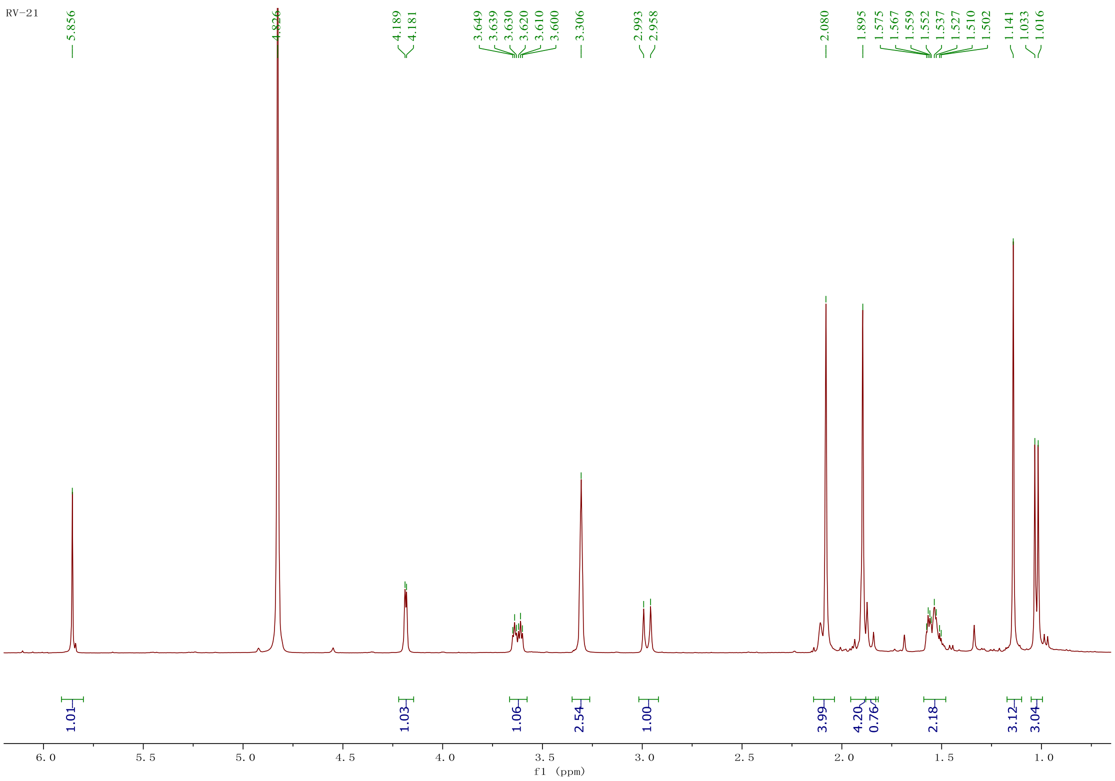

^1H NMR spectrum of **2**

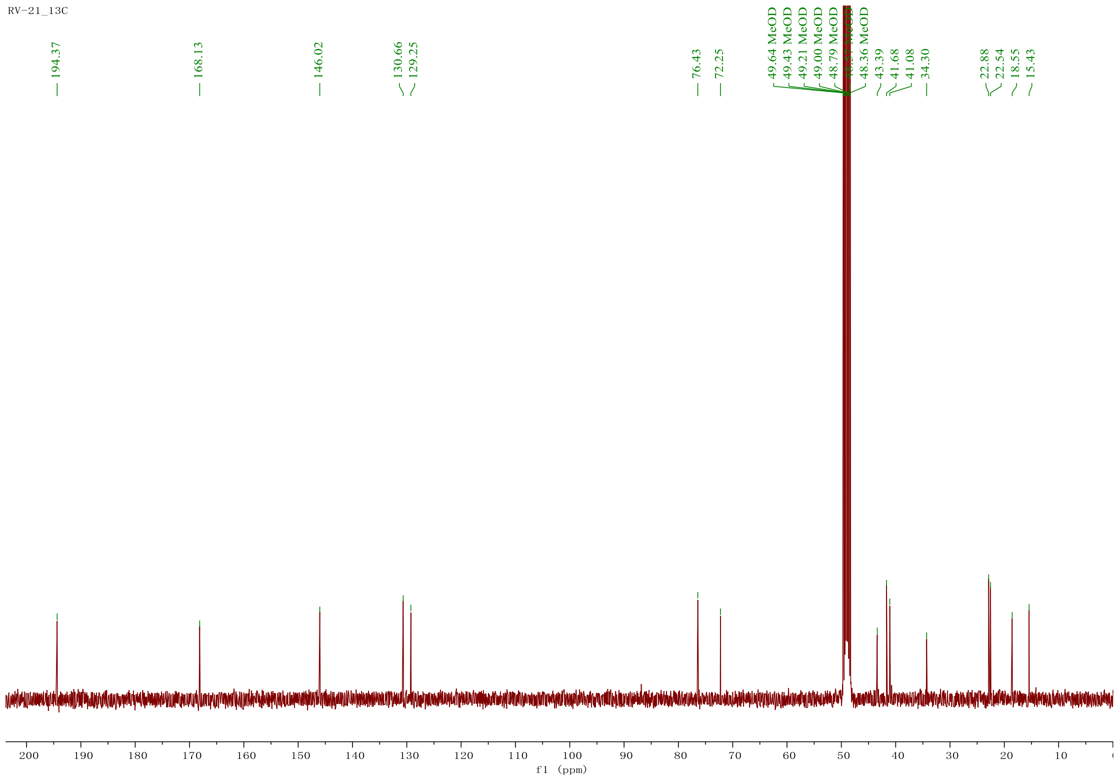

^{13}C NMR spectrum of **2**

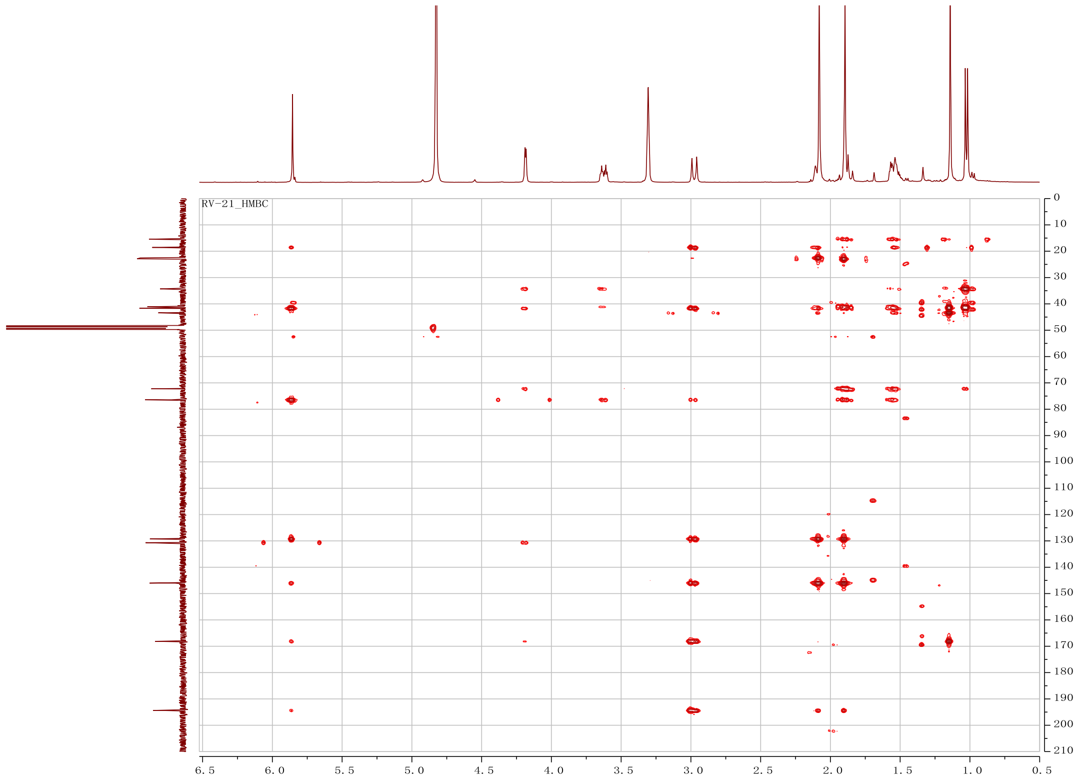

HMBC spectrum of **2**

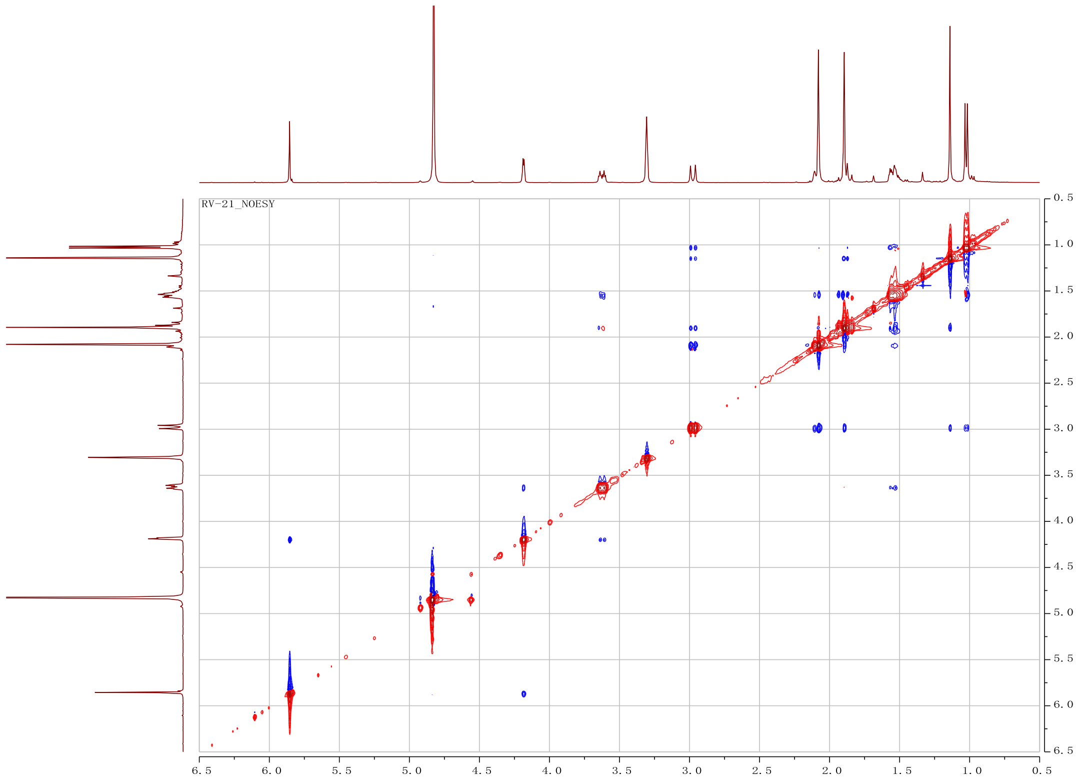

NOESY spectrum of **2**

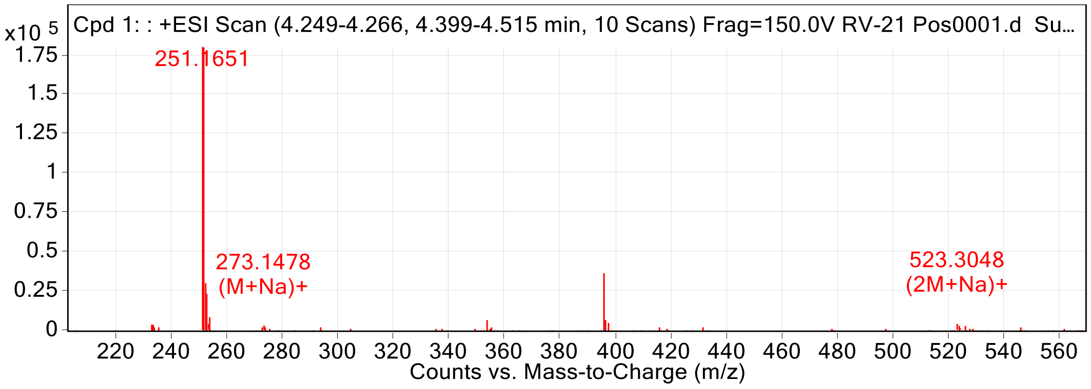

m/z	*Calc m/z*	Diff(ppm)	z	Abund	Formula	Ion
251.1651	251.1642	3.76	1	180122.1	$C_{15}H_{23}O_3$	$(M+H)^+$

HRESIMS spectrum of **2**

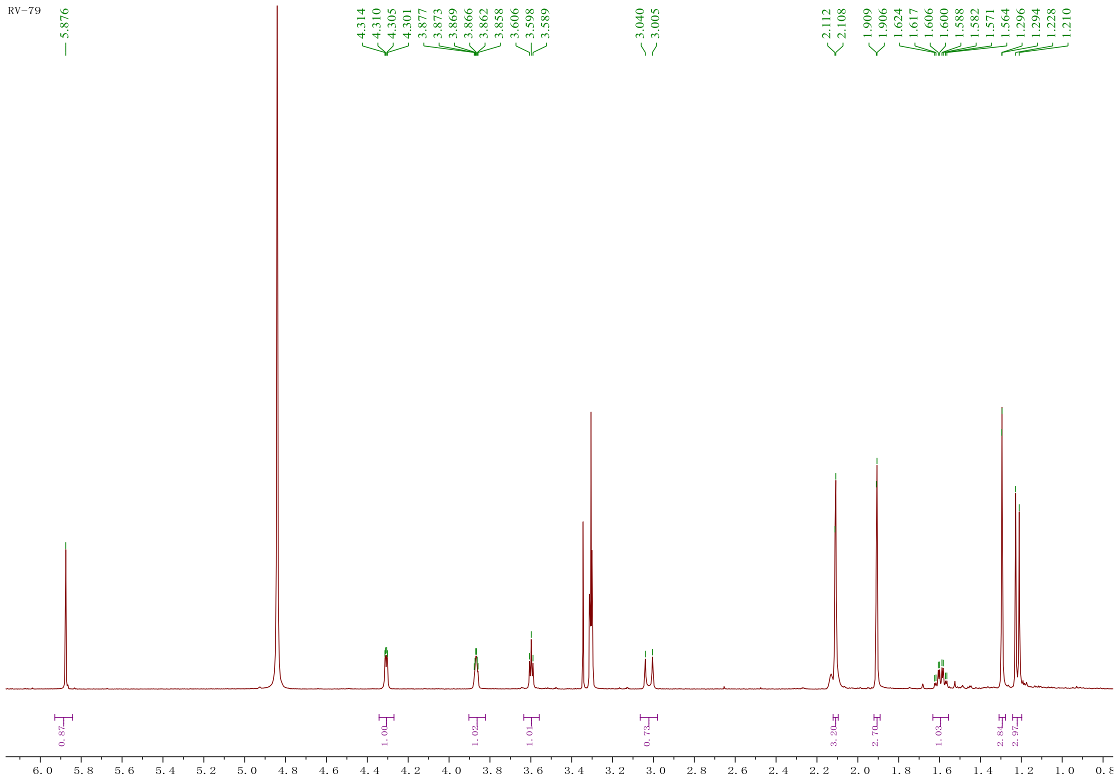

^{1}H NMR spectrum of **3**

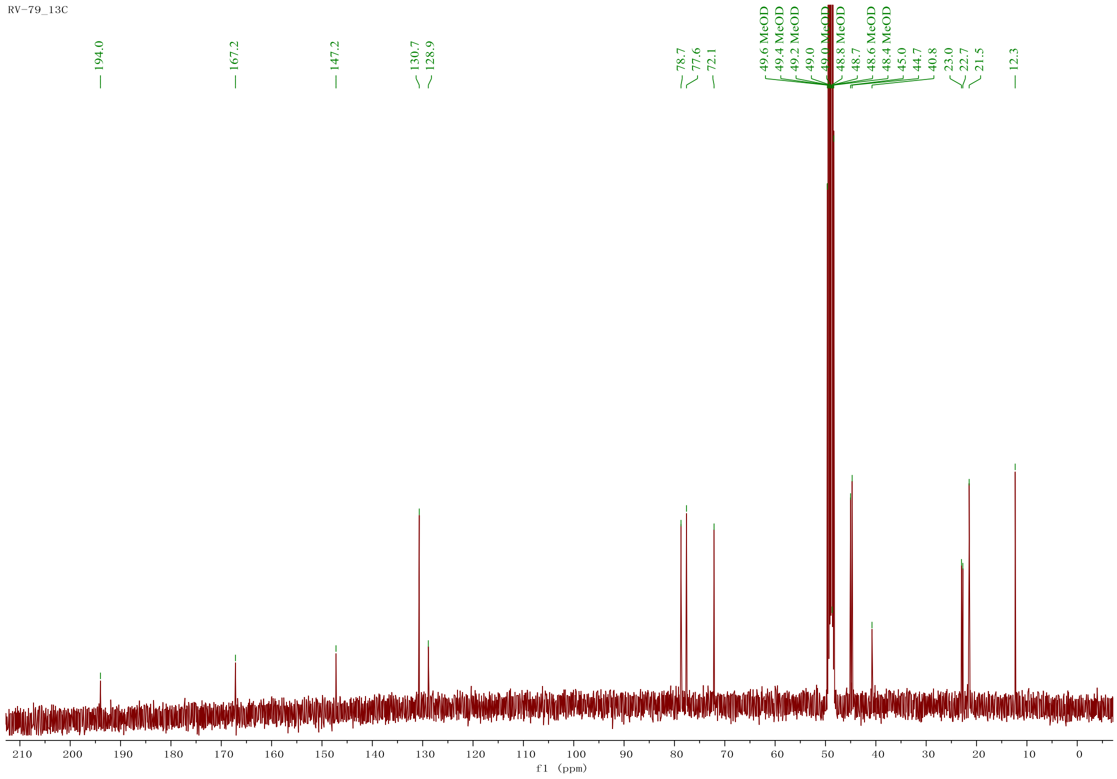

^{13}C NMR spectrum of **3**

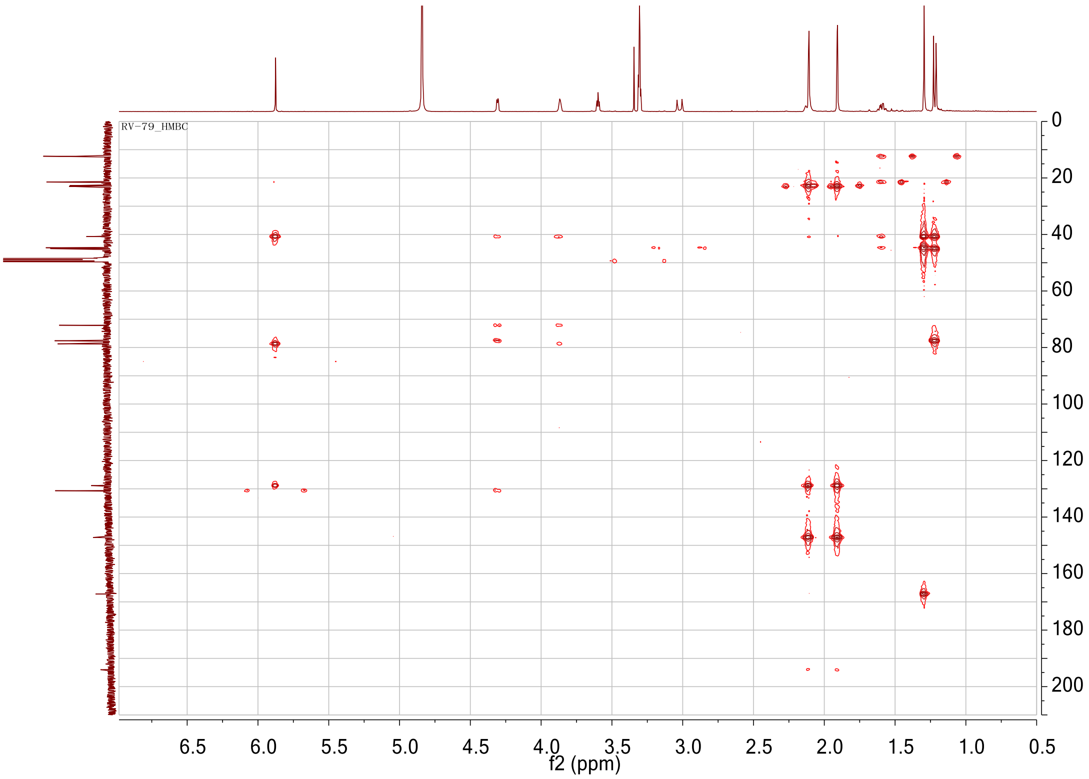

HMBC spectrum of **3**

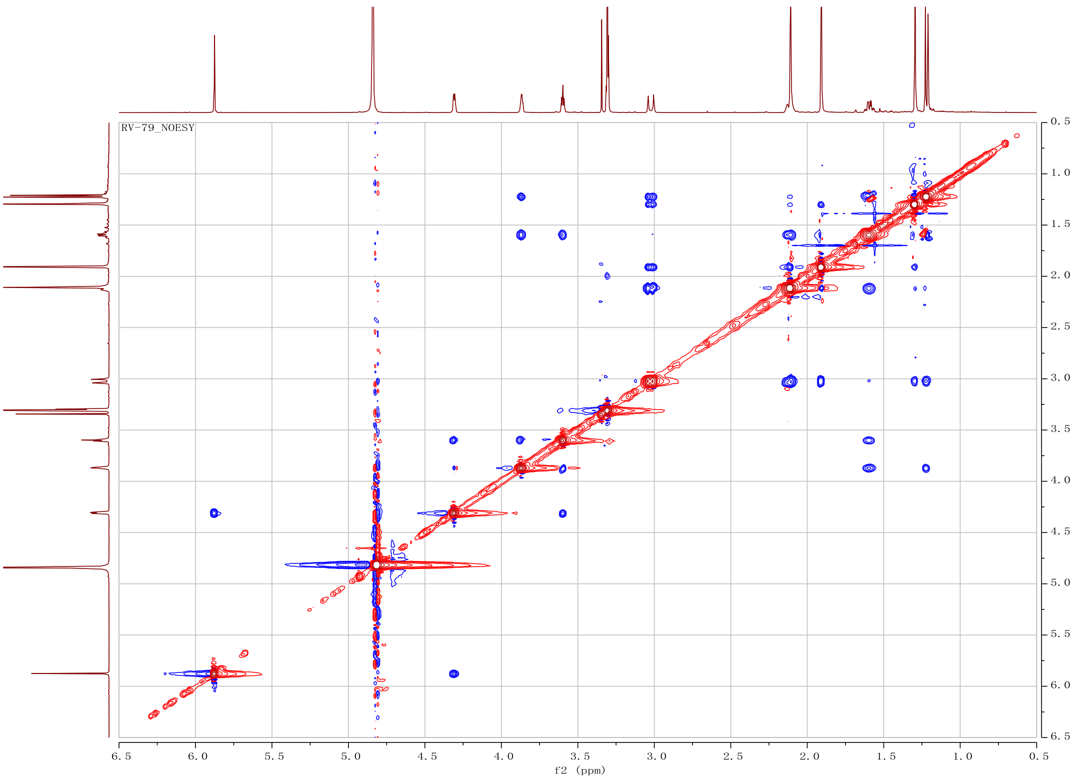

NOESY spectrum of **3**

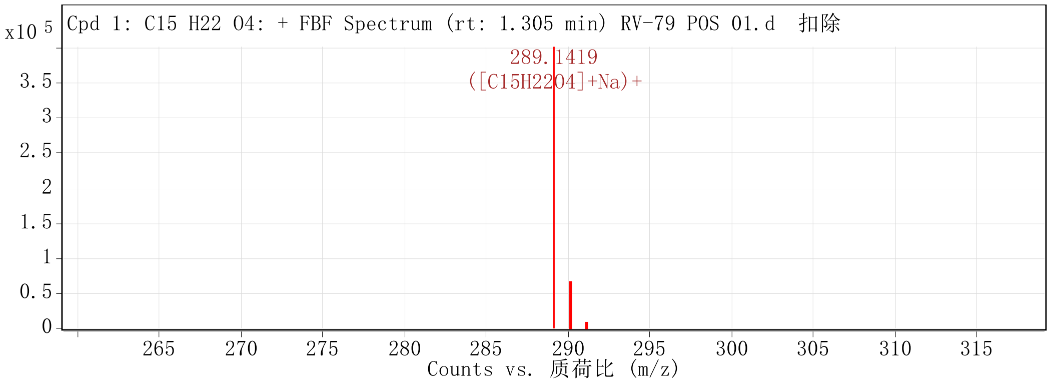

m/z	*Calc m/z*	Diff(ppm)	z	Abund	Formula	Ion
289.1419	289.1410	3.11	1	402304.38	$C_{15}H_{22}O_4Na$	$(M+ Na)^+$

HRESIMS spectrum of **3**

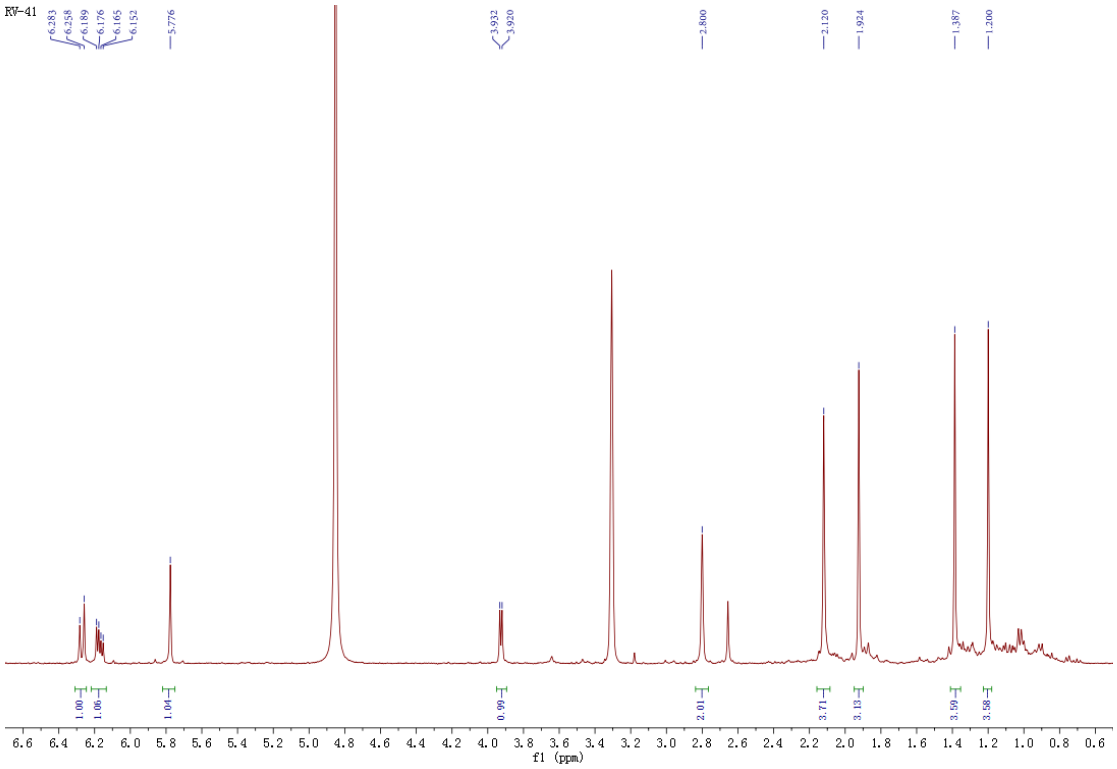

^{1}H NMR spectrum of **4**

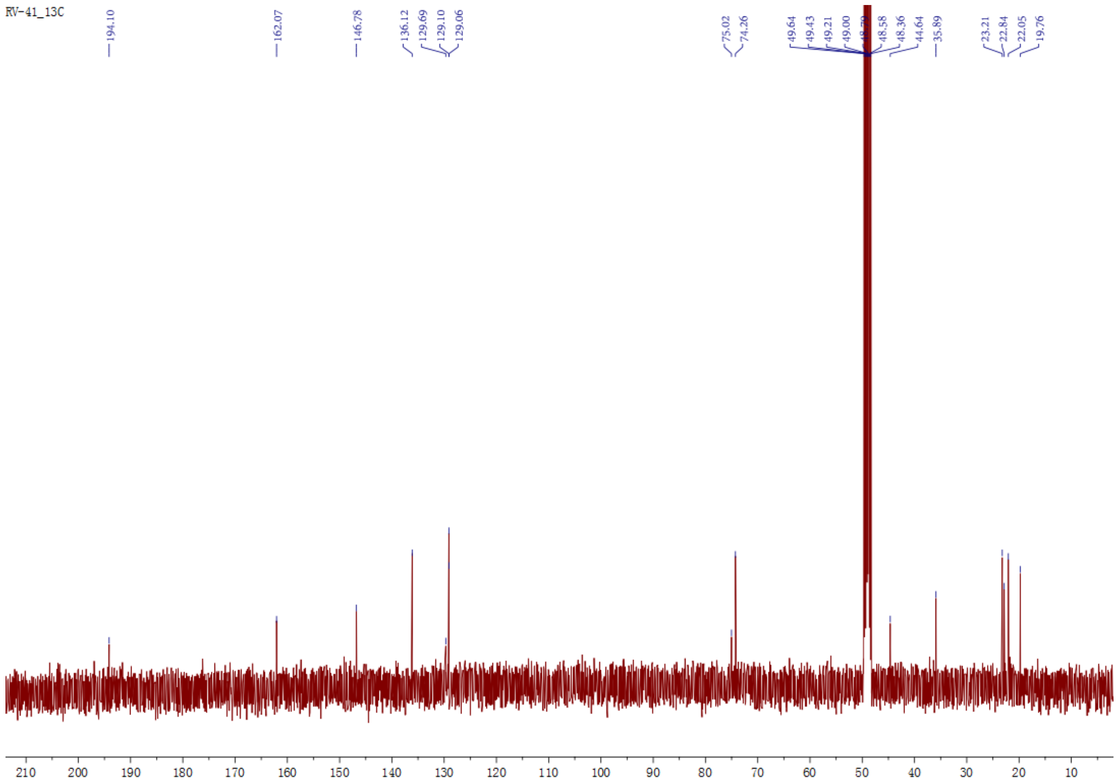

^{13}C NMR spectrum of **4**

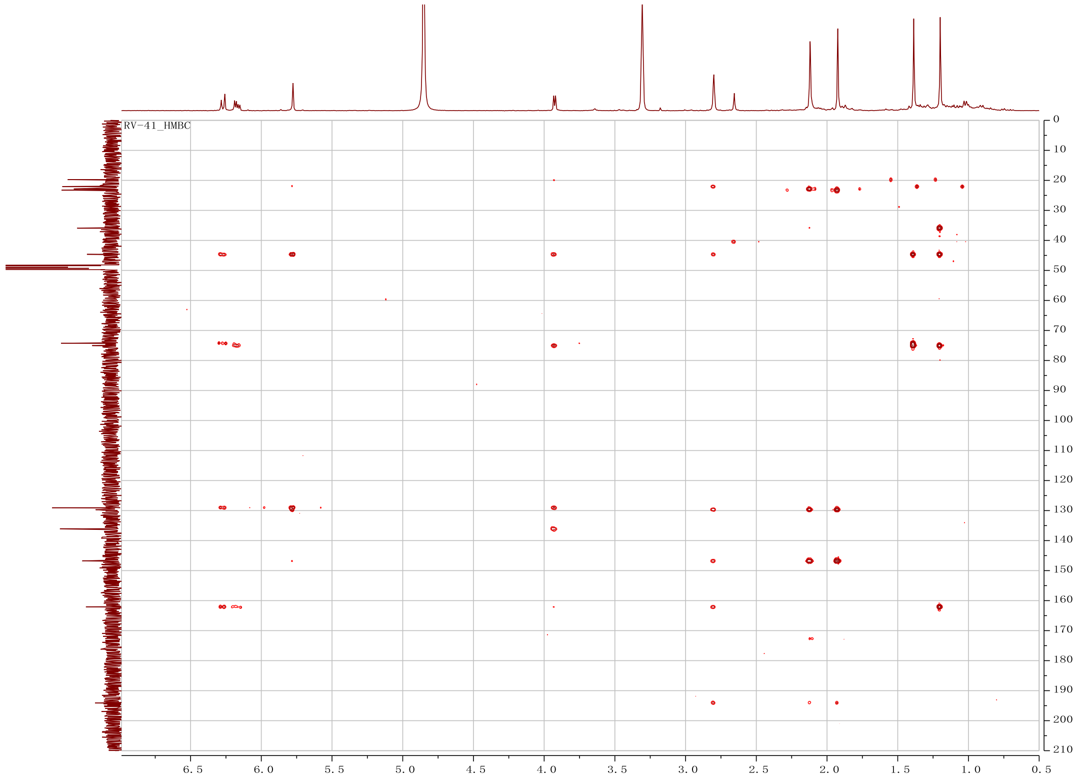

HMBC spectrum of **4**

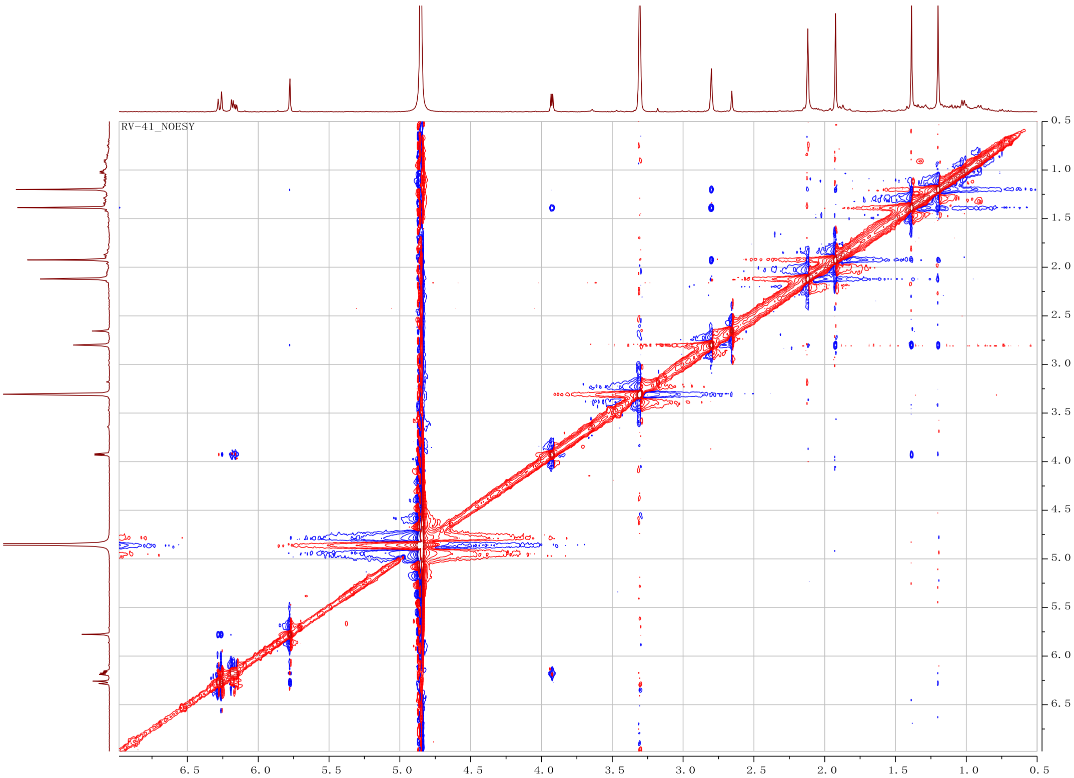

NOESY spectrum of **4**

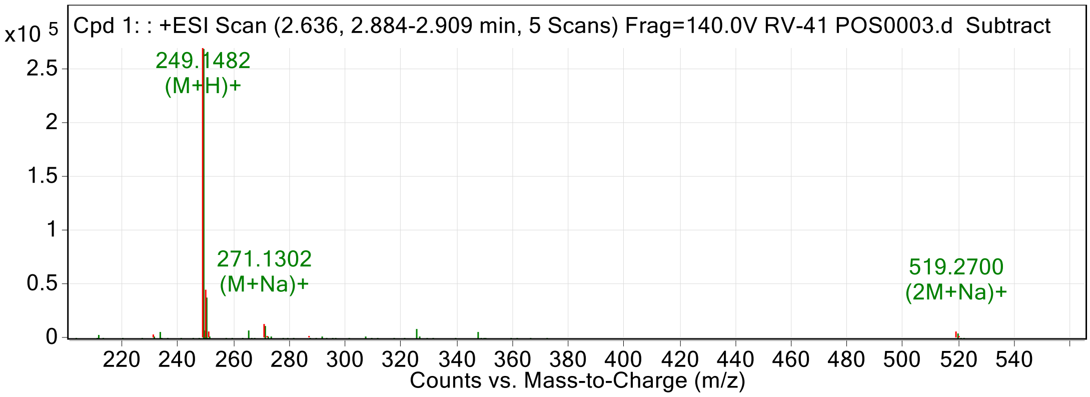

m/z	*Calc m/z*	Diff(ppm)	z	Abund	Formula	Ion
249.1482	249.1485	-1.33		270074.8	$C_{15}H_{21}O_3$	$(M+H)^+$
271.1302	271.1305	-0.95	1	12518.7	$C_{15}H_{20}NaO_3$	$(M+Na)^+$

HRESIMS spectrum of **4**

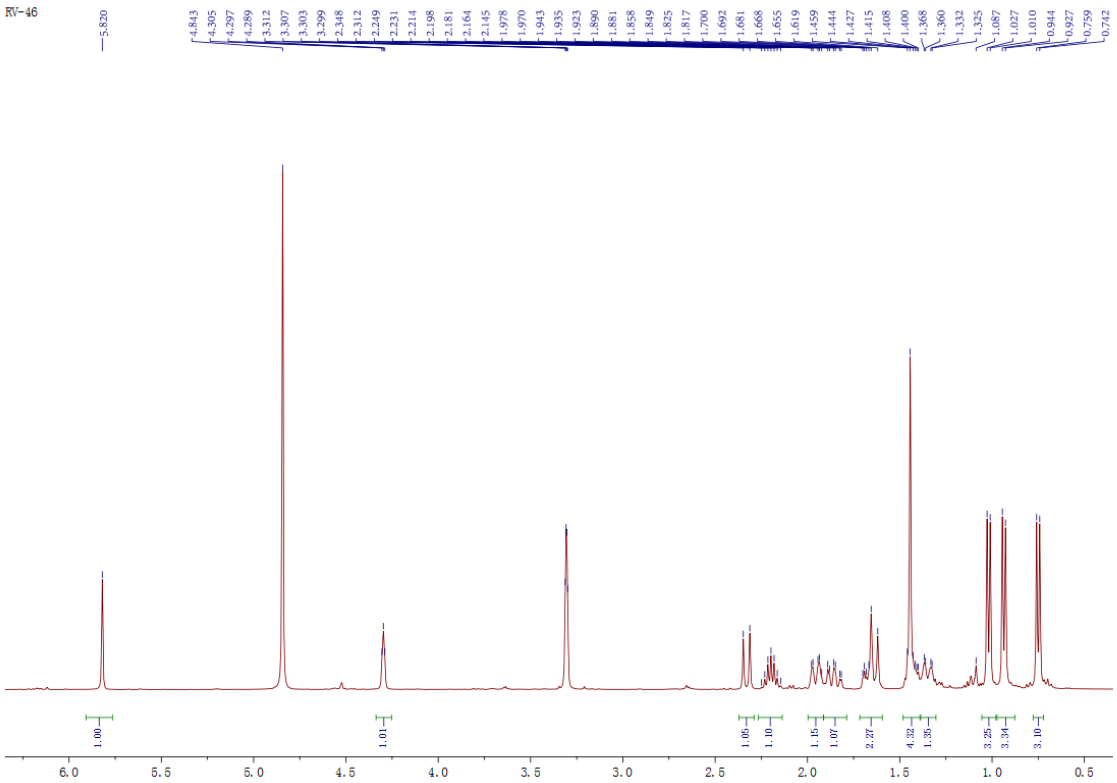

^{1}H NMR spectrum of **5**

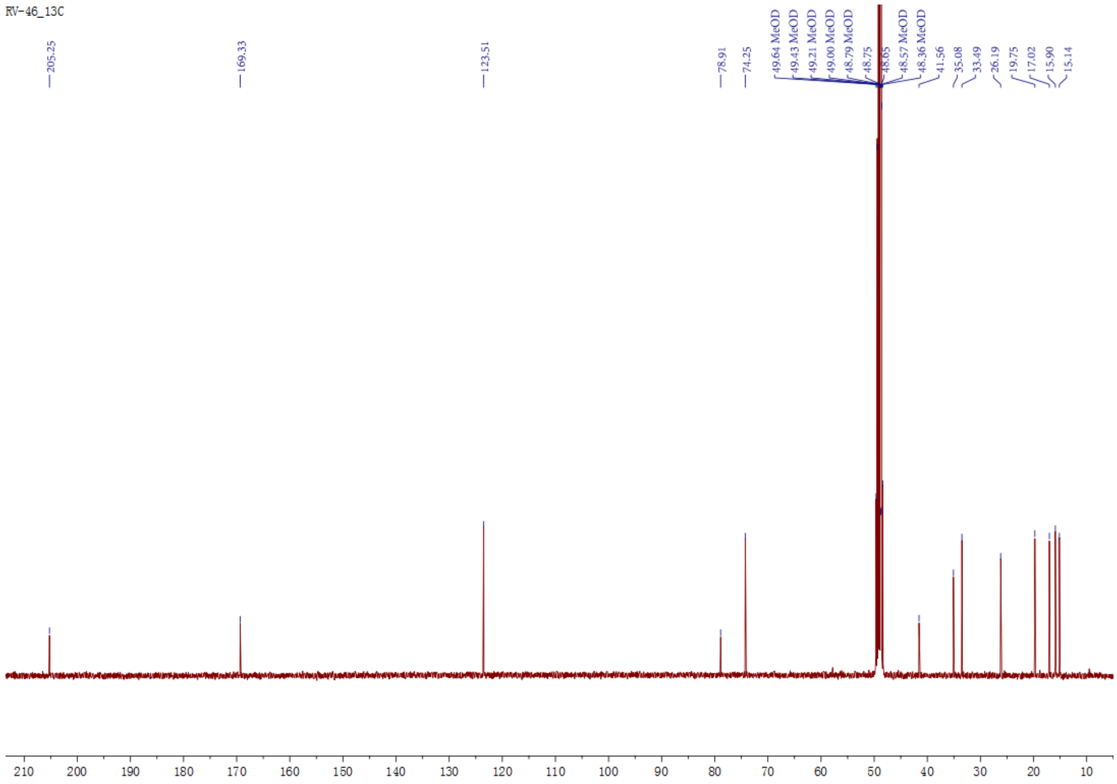

^{13}C NMR spectrum of **5**

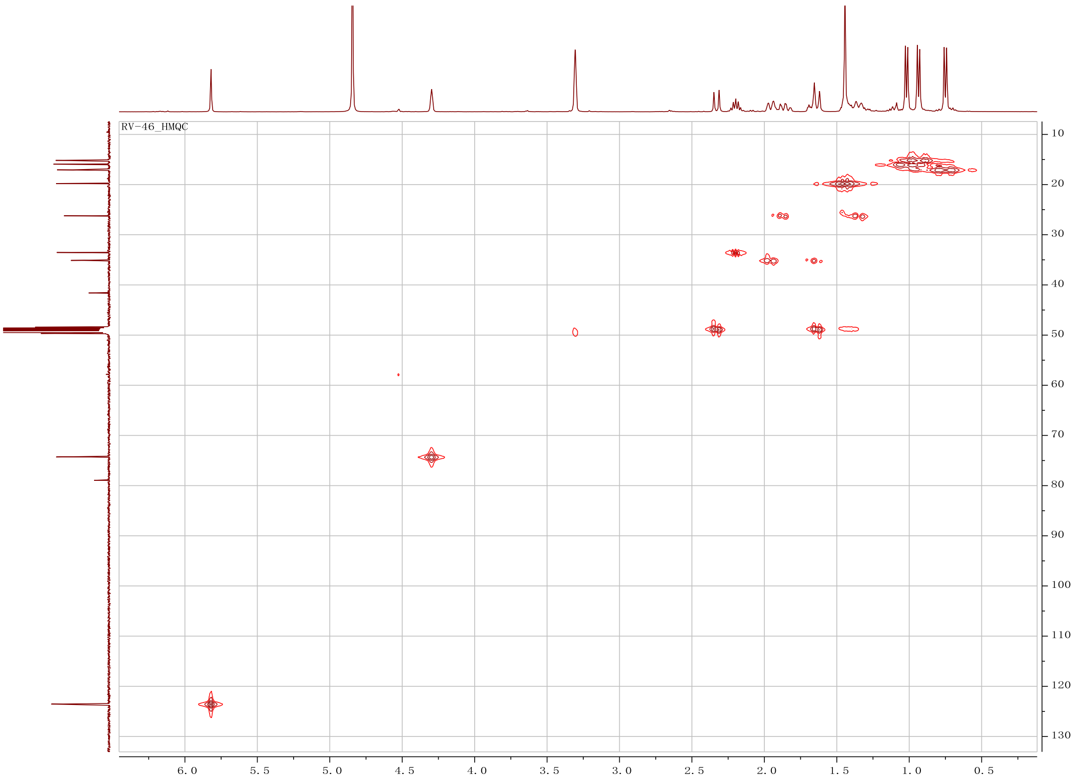

HMQC spectrum of **5**

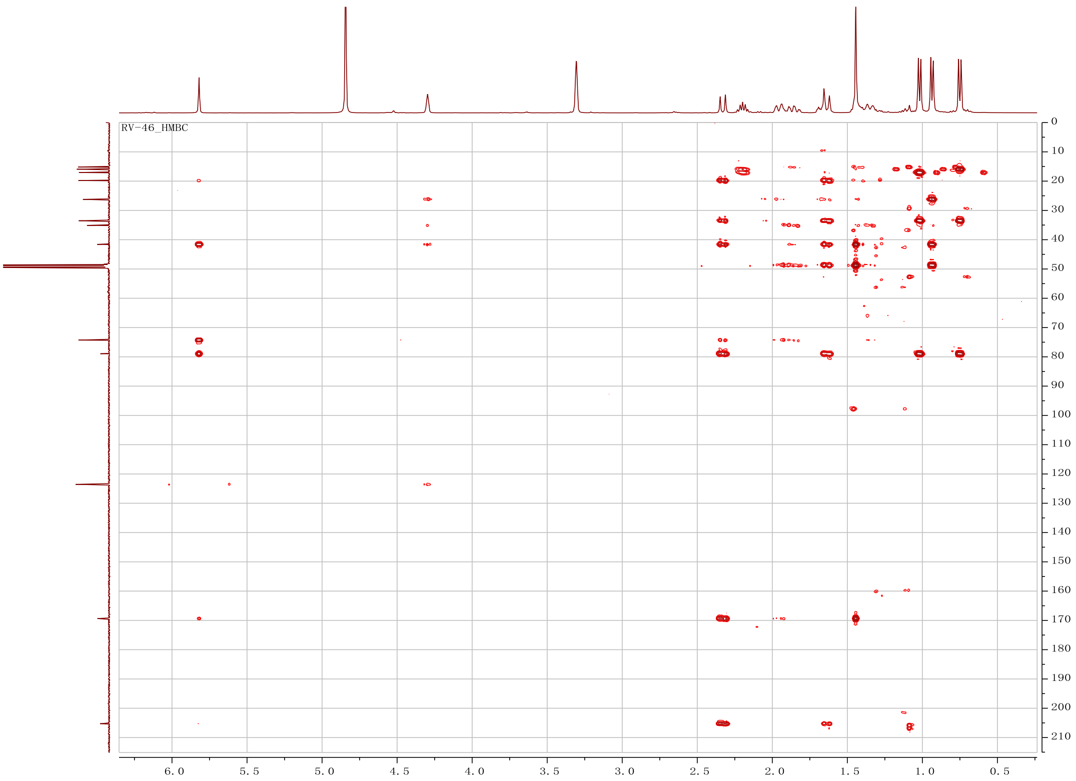

HMBC spectrum of **5**

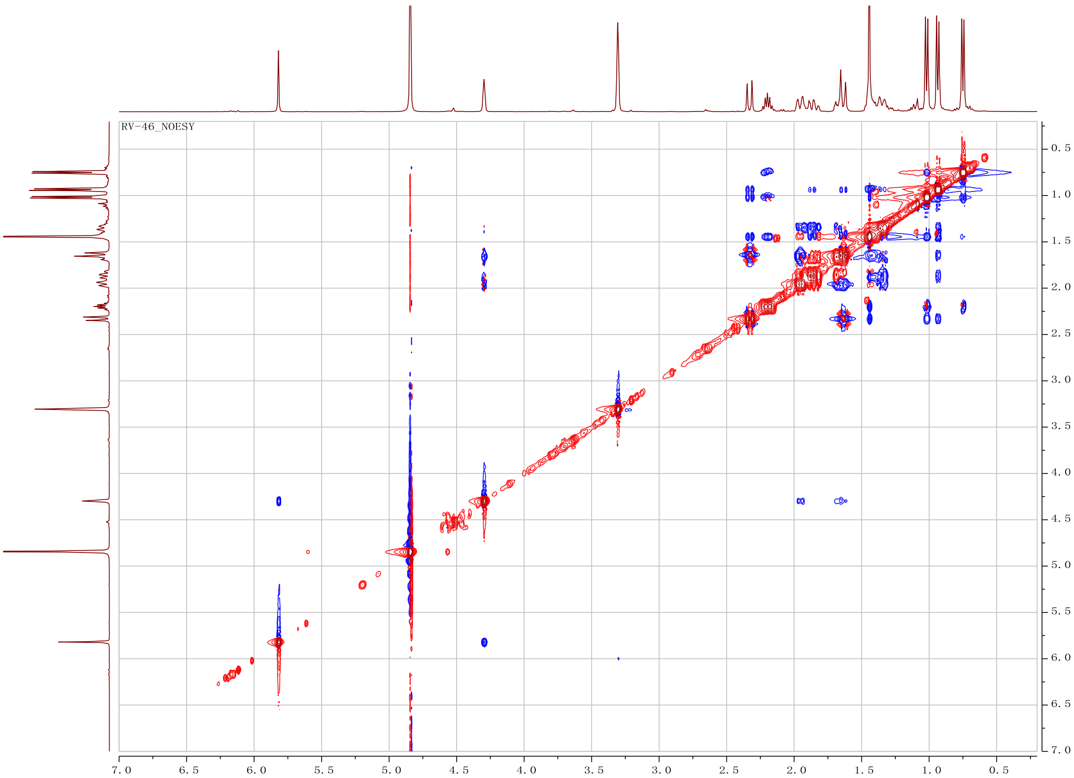

NOESY spectrum of **5**

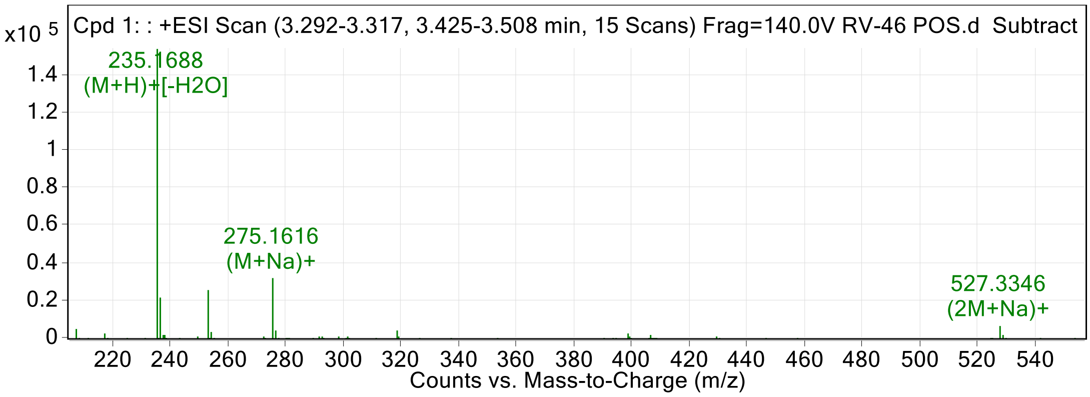

m/z	***Calc m/z***	**Diff(ppm)**	**z**	**Abund**	**Formula**	**Ion**
235.1688	235.1693	-2.02	1	153907.2	$C_{15}H_{23}O_2$	$(M+H)^+[-H_2O]$
253.1798	253.1798	-0.08	1	25897.4	$C_{15}H_{25}O_3$	$(M+H)^+$
275.1616	275.1618	-0.76	1	32605.2	$C_{15}H_{24}NaO_3$	$(M+Na)^+$
527.3346	527.3343	0.47	1	7395.4	$C_{30}H_{48}NaO_6$	$(2M+Na)^+$

HRESIMS spectrum of **5**

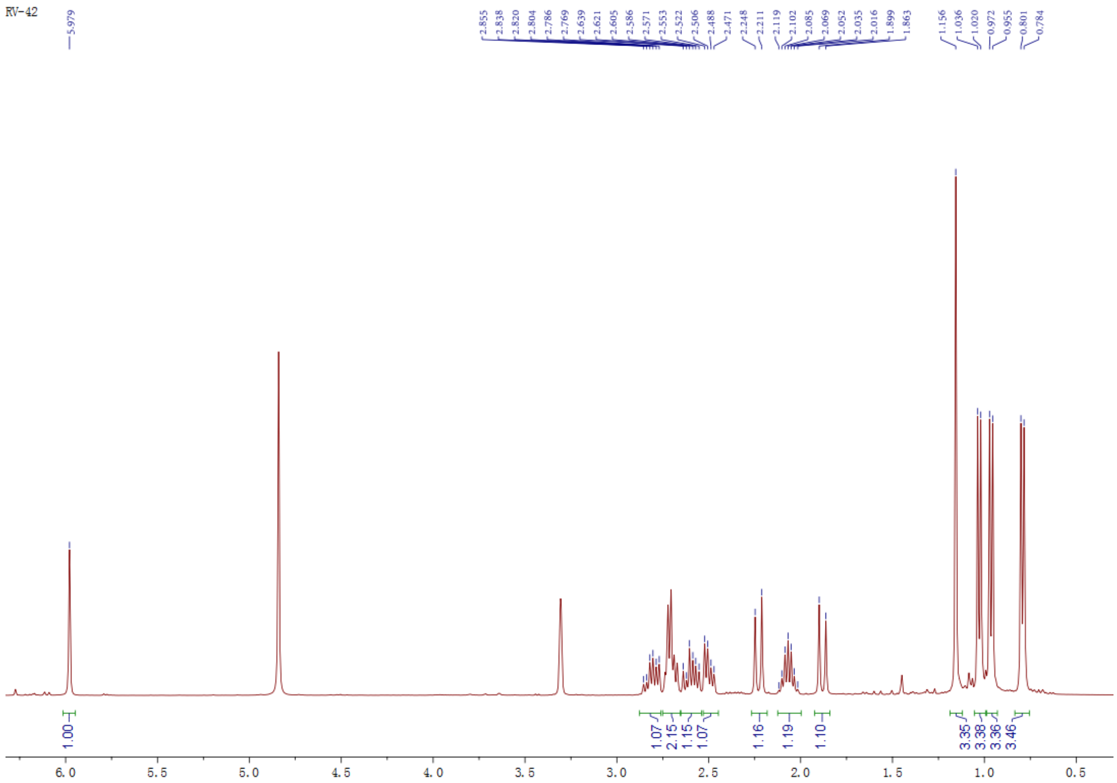

^{1}H NMR spectrum of **6**

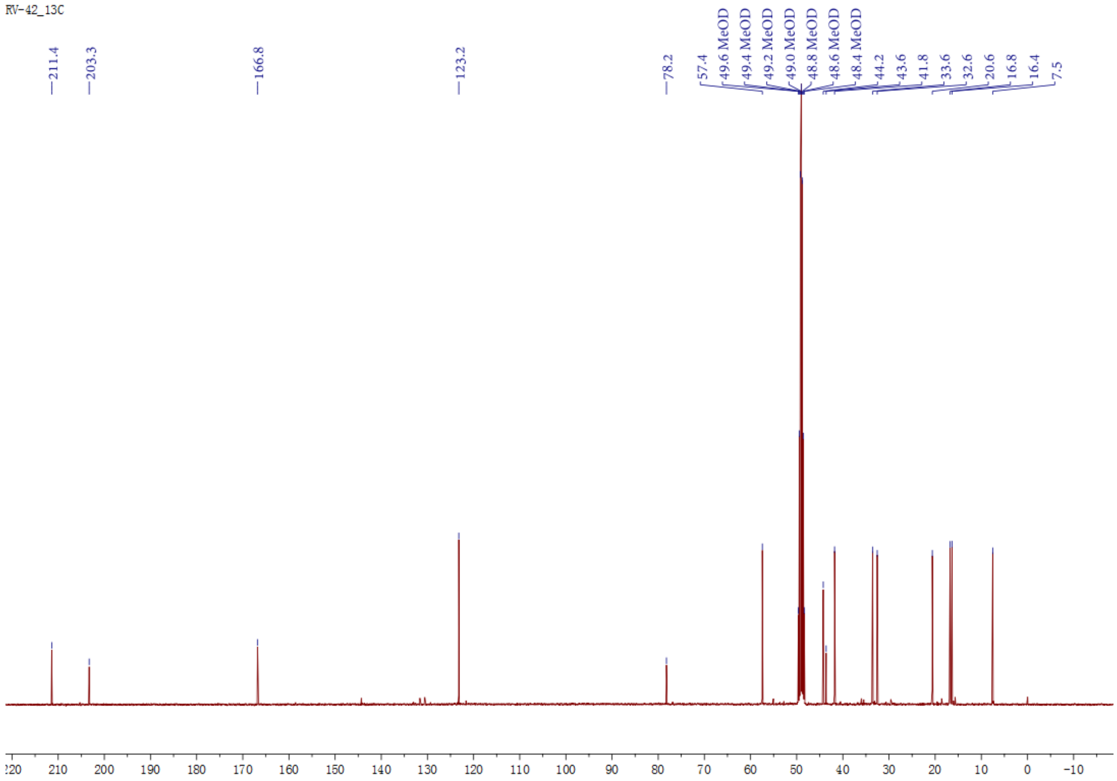

^{13}C NMR spectrum of **6**

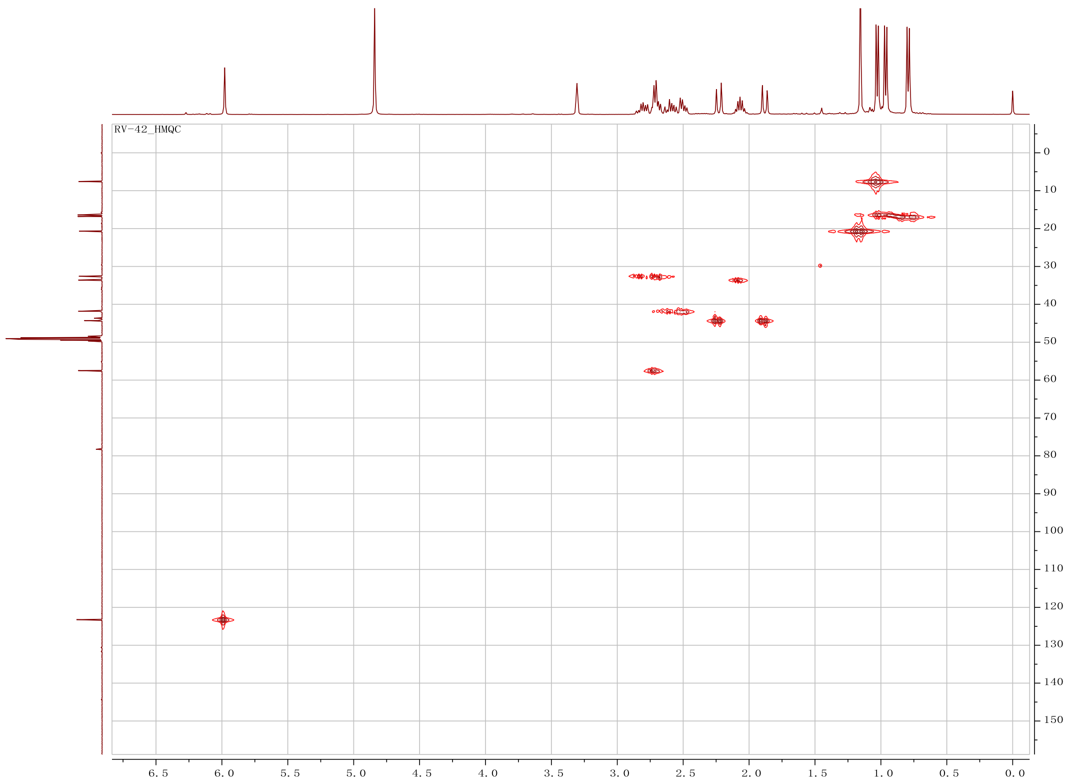

HMQC of **6**

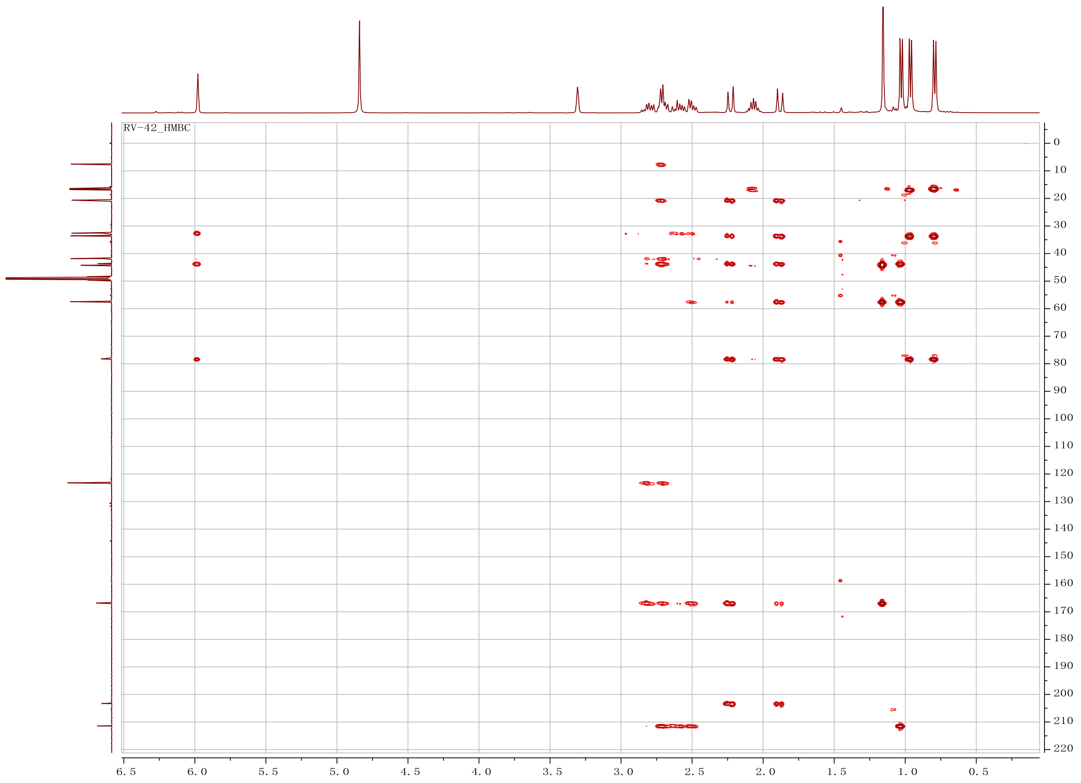

HMBC spectrum of **6**

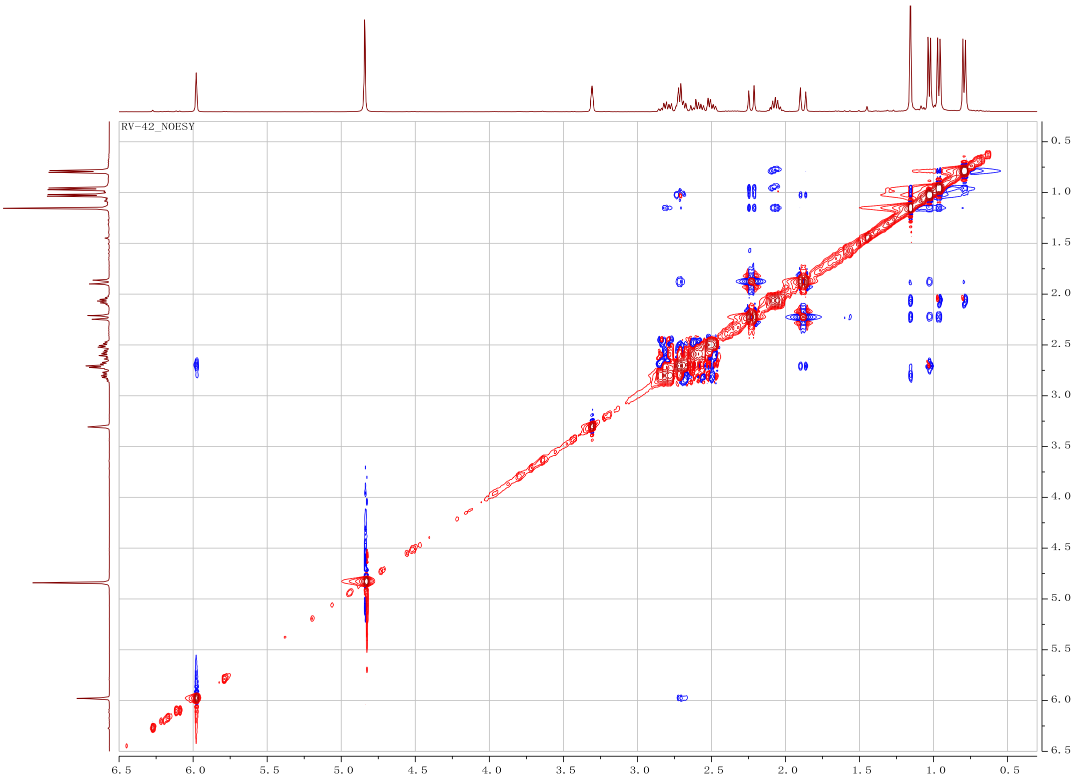

NOESY spectrum of **6**

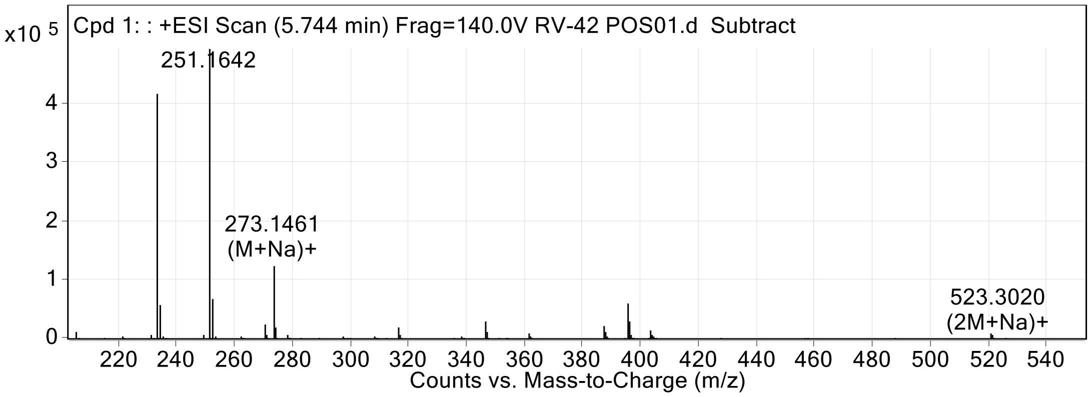

m/z	*Calc m/z*	Diff(ppm)	Z	Abund	Formula	Ion
233.1535	233.1536	-0.58	1	419769.5	$C_{15}H_{21}O_2$	$(M+H)^+[-H_2O]$
251.1642	251.1642	0.06		495166.2	$C_{15}H_{23}O_3$	$(M+H)^+$
273.1461	273.1461	-0.19	1	125710.6	$C_{15}H_{22}NaO_3$	$(M+Na)^+$
523.3020	523.3030	-1.94	1	695.4	$C_{30}H_{44}NaO_6$	$(2M+Na)^+$

HRESIMS spectrum of **6**

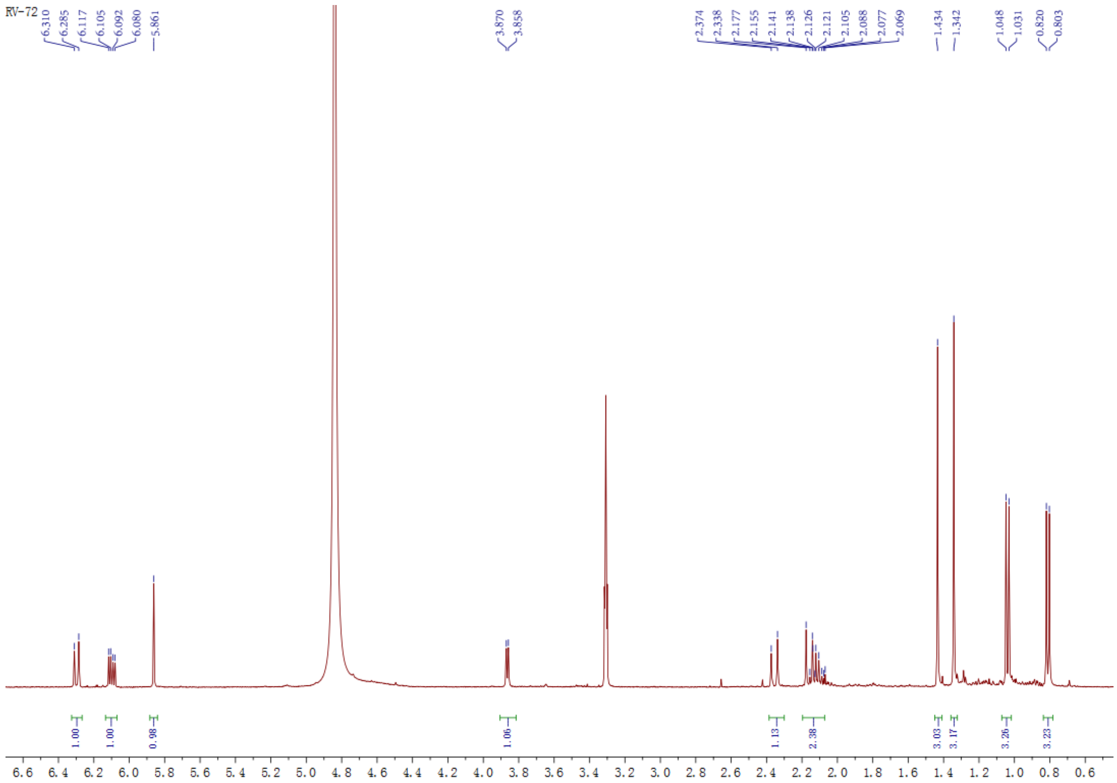

^{1}H NMR spectrum of **7**

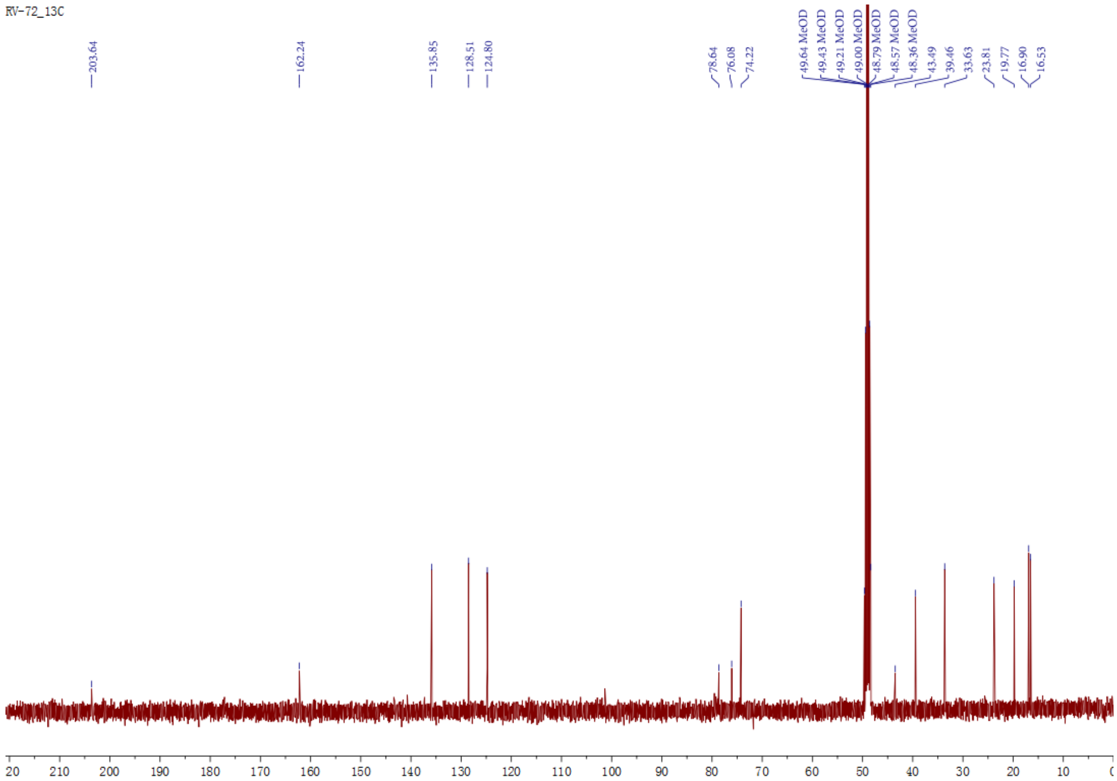

^{13}C NMR spectrum of **7**

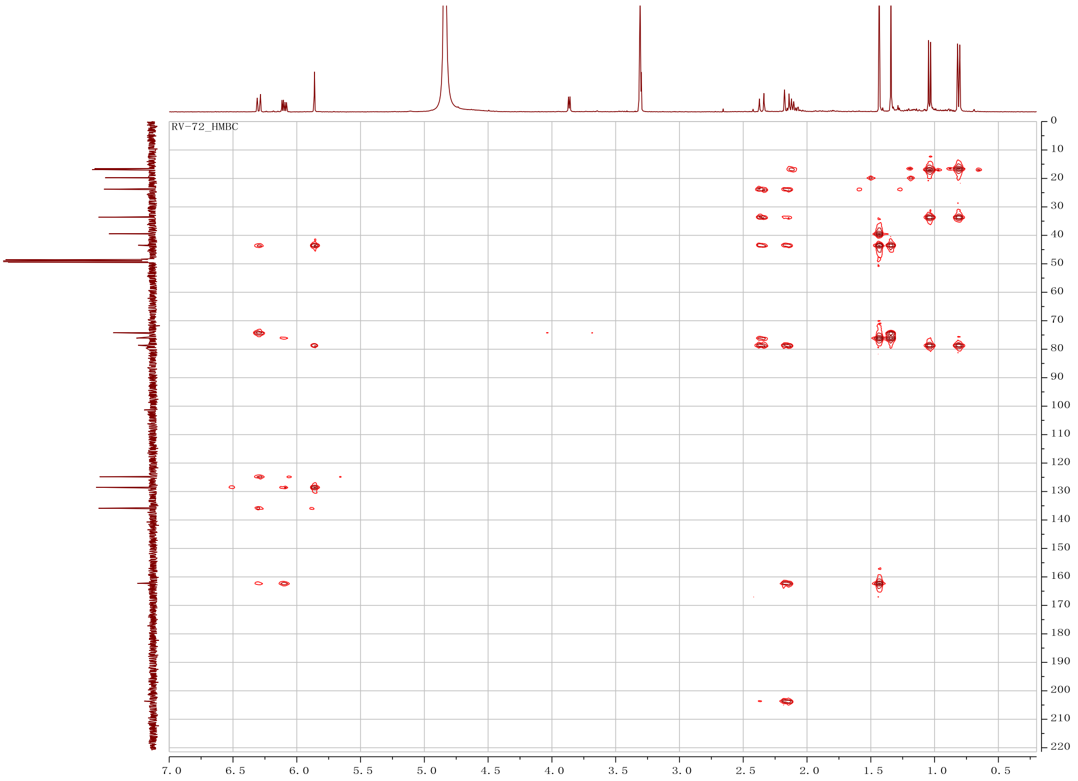

HMBC spectrum of **7**

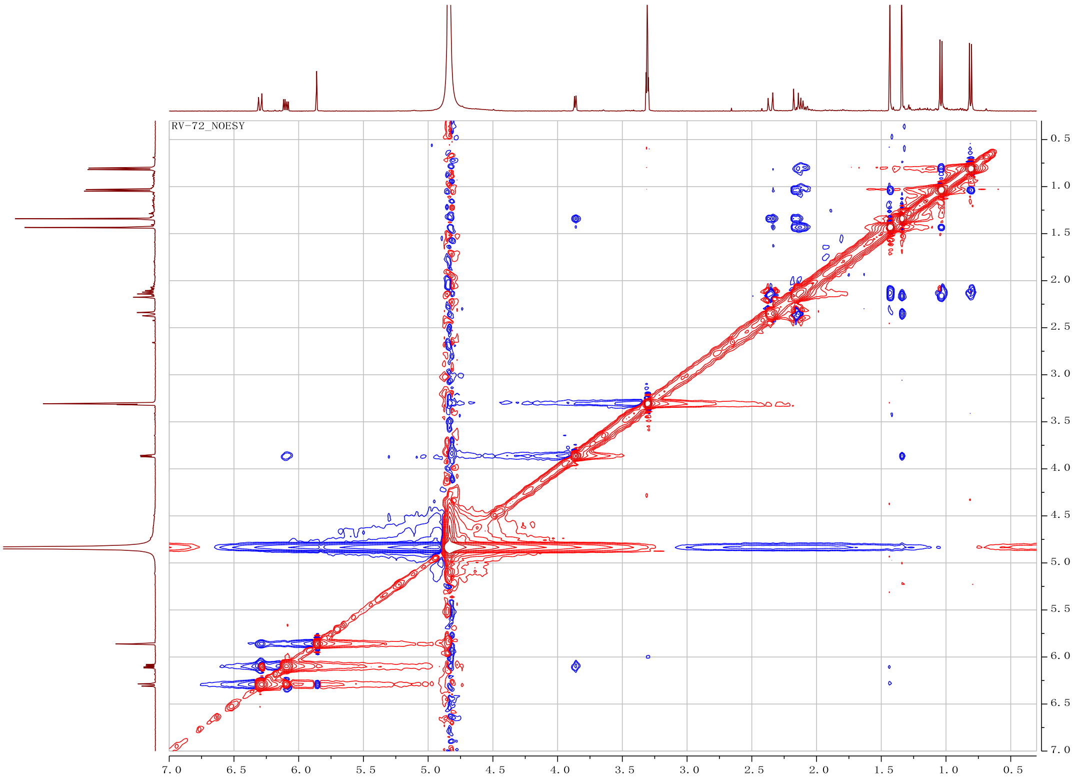

NOESY spectrum of **7**

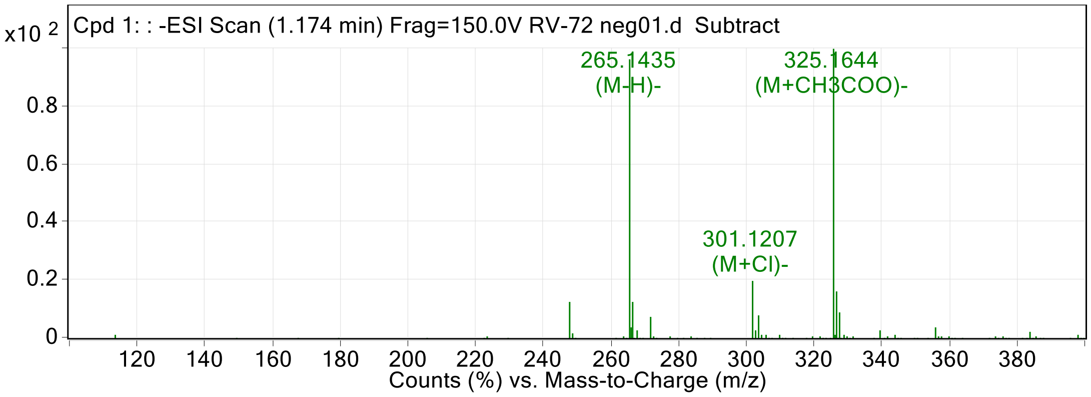

m/z	*Calc m/z*	Diff(ppm)	z	Abund	Formula	Ion
265.1435	265.1445	-3.79	-1	541208	$C_{15}H_{21}O_4$	$(M-H)^-$
301.1207	301.1212	-1.86	-1	113309.6	$C_{15}H_{22}ClO_4$	$(M+Cl)^-$
325.1644	325.1657	-3.87	-1	562816.9	$C_{17}H_{25}O_6$	$(M+CH_3COO)^-$

HRESIMS spectrum of **7**

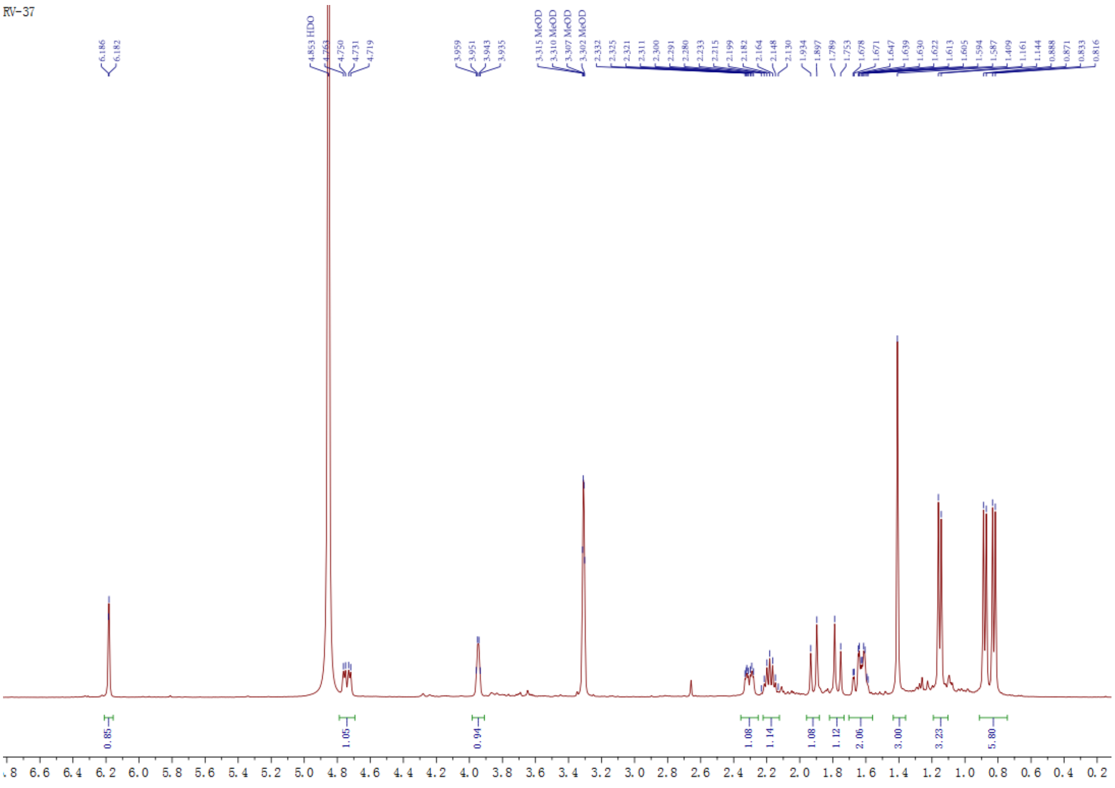

^{1}H NMR spectrum of **8**

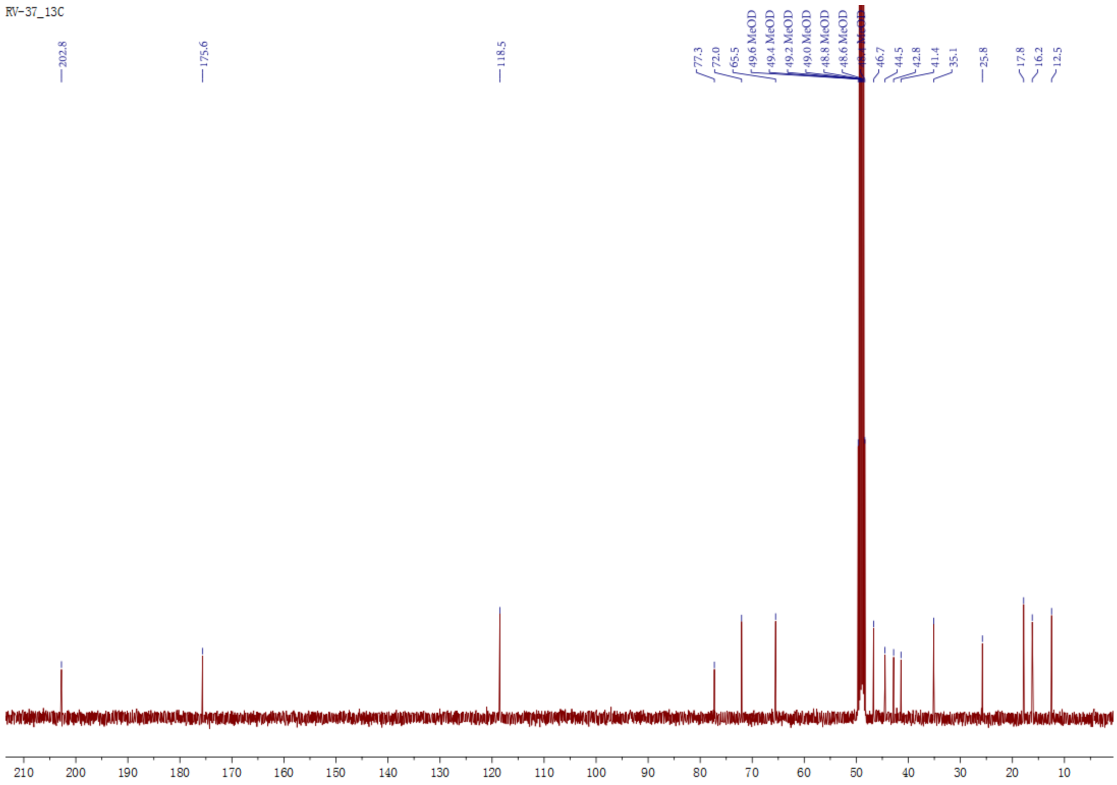

^{13}C NMR spectrum of **8**

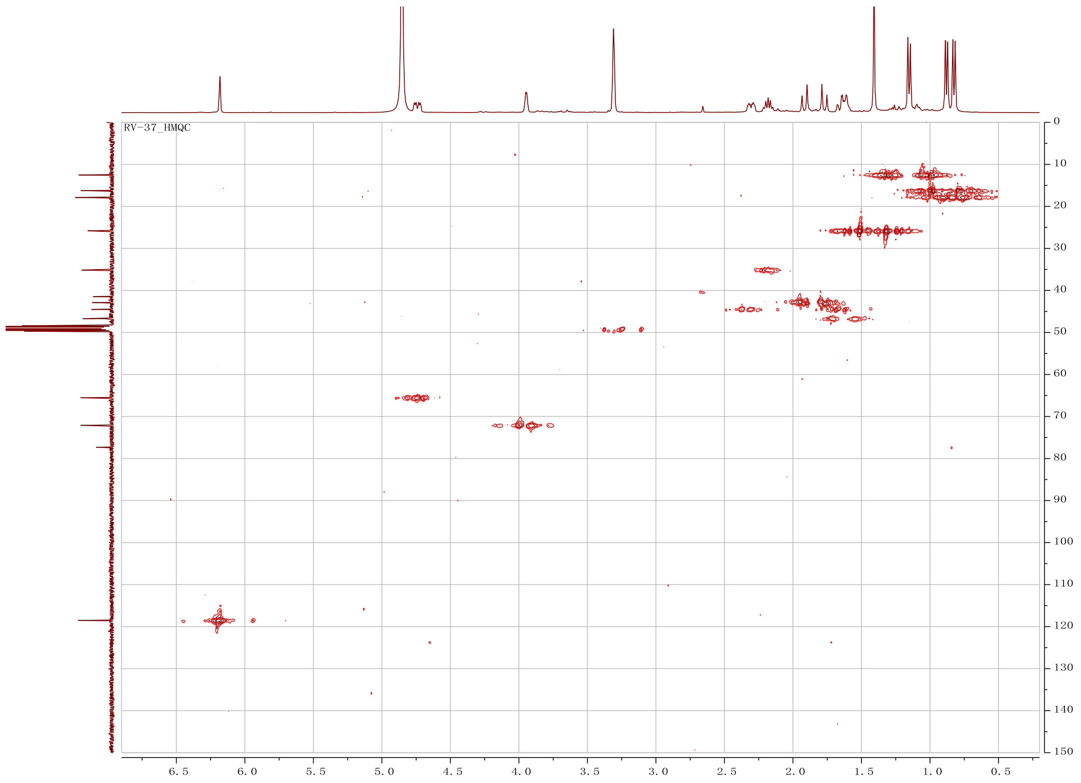

HMQC spectrum of **8**

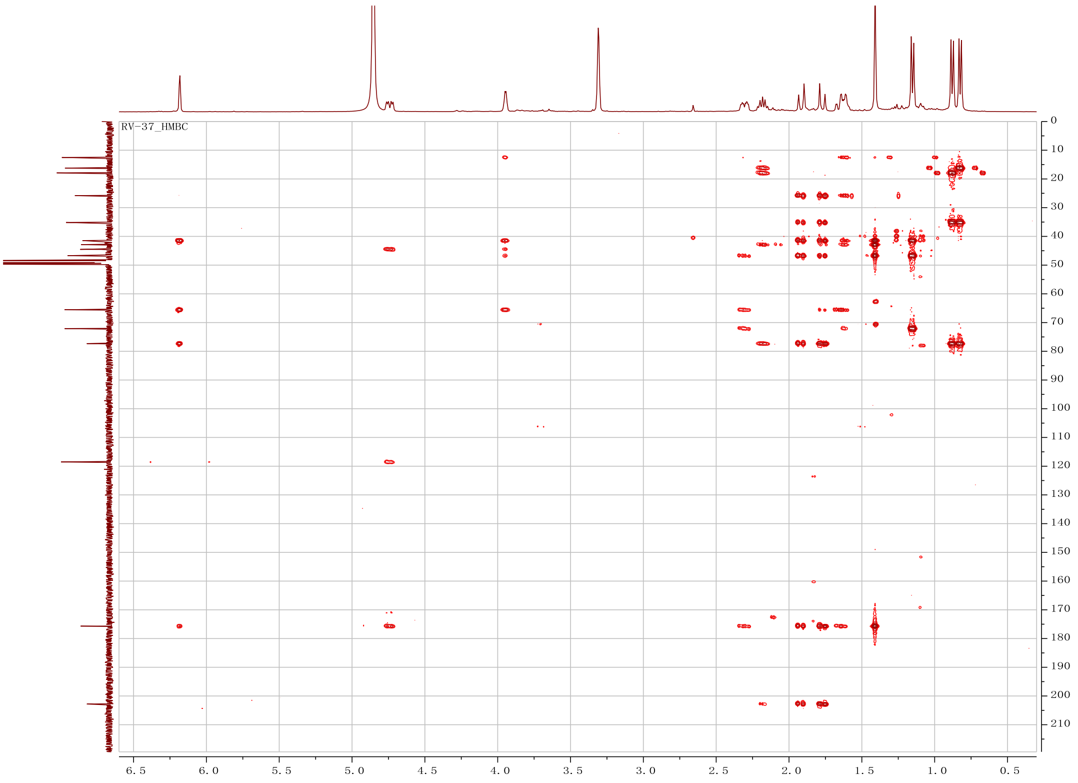

HMBC spectrum of **8**

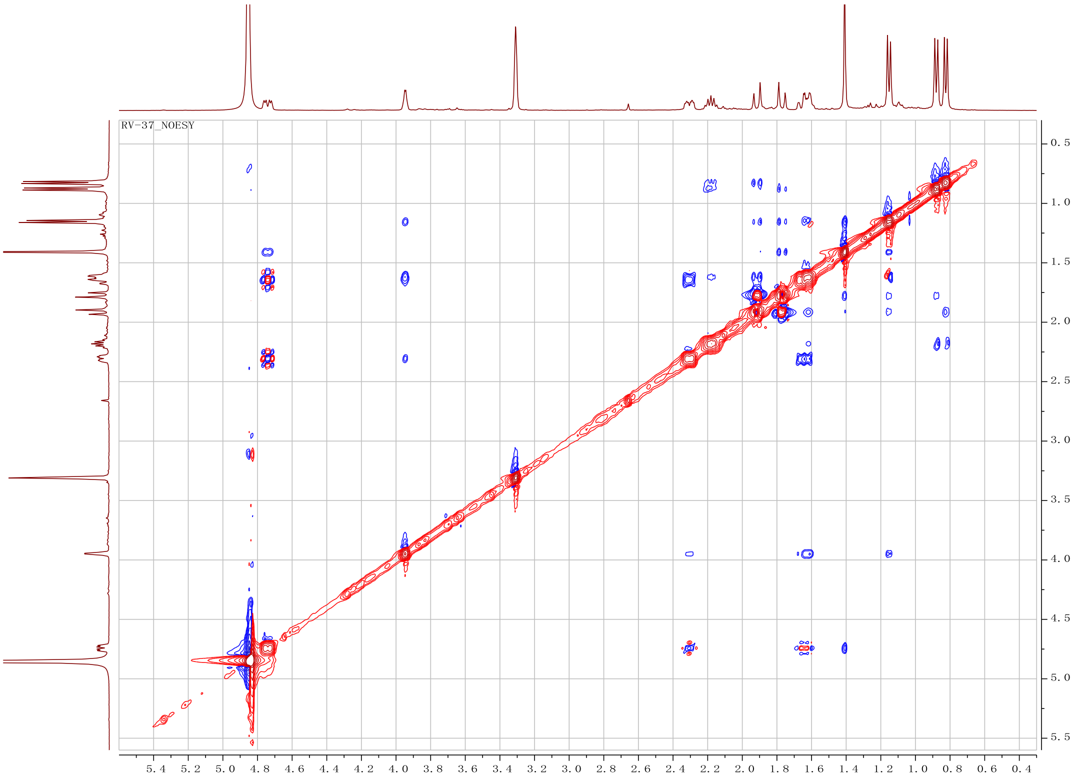

NOESY spectrum of **8**

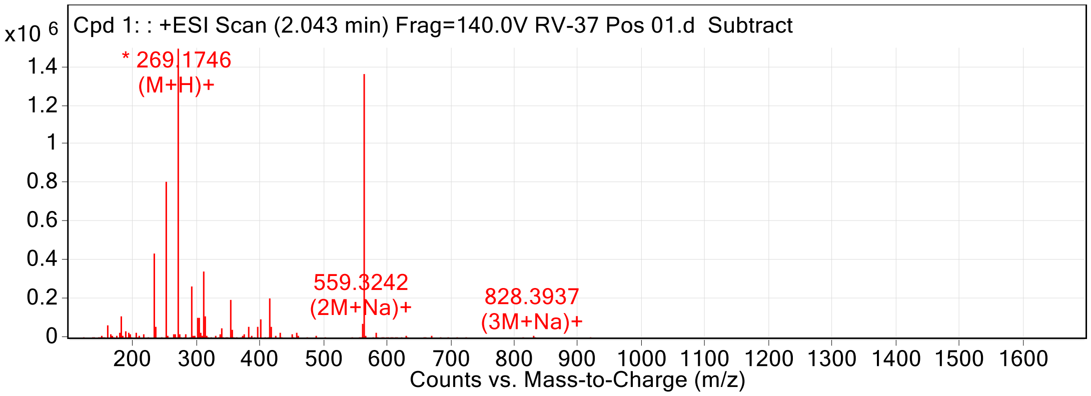

m/z	*Calc m/z*	Diff(ppm)	z	Abund	Formula	Ion
269.1746	269.1747	-0.57		1497296	$C_{15}H_{25}O_4$	$(M+H)^+$
291.1563	291.1567	-1.24	1	269051.4	$C_{15}H_{24}NaO_4$	$(M+Na)^+$
559.3242	559.3241	0.07	1	74474.2	$C_{30}H_{48}NaO_8$	$(2M+Na)^+$

HRESIMS spectrum of **8**

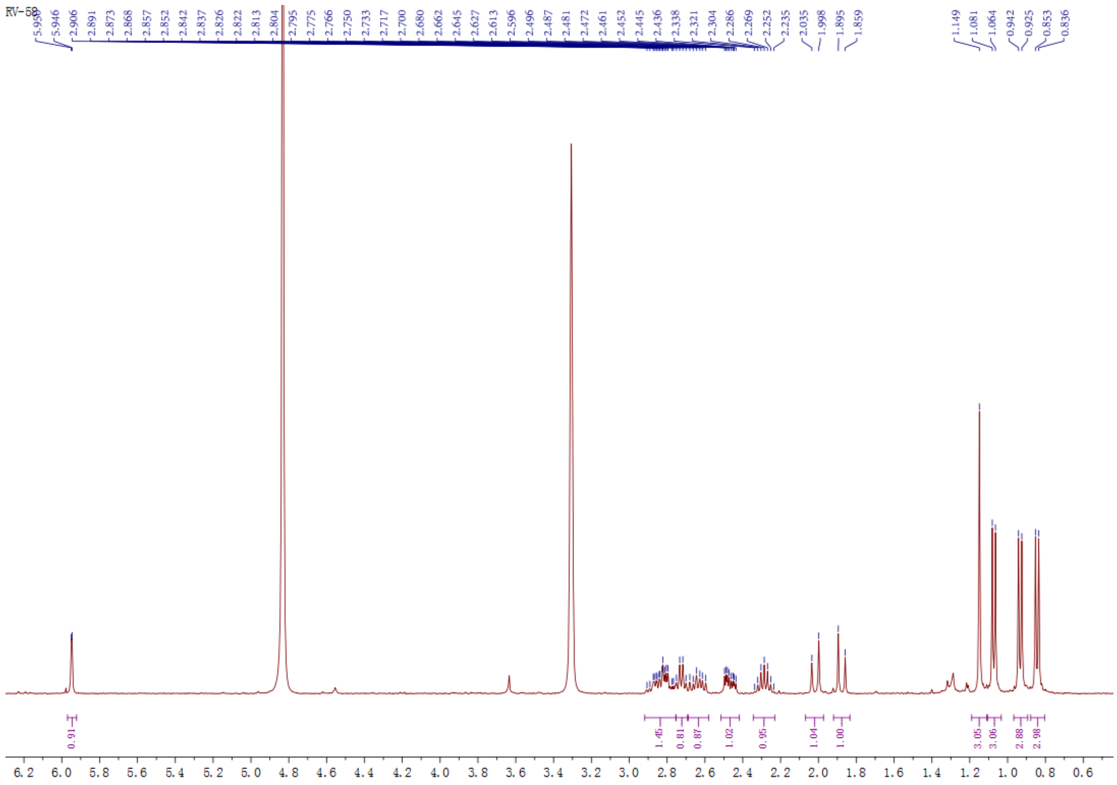

^{1}H NMR spectrum of **9**

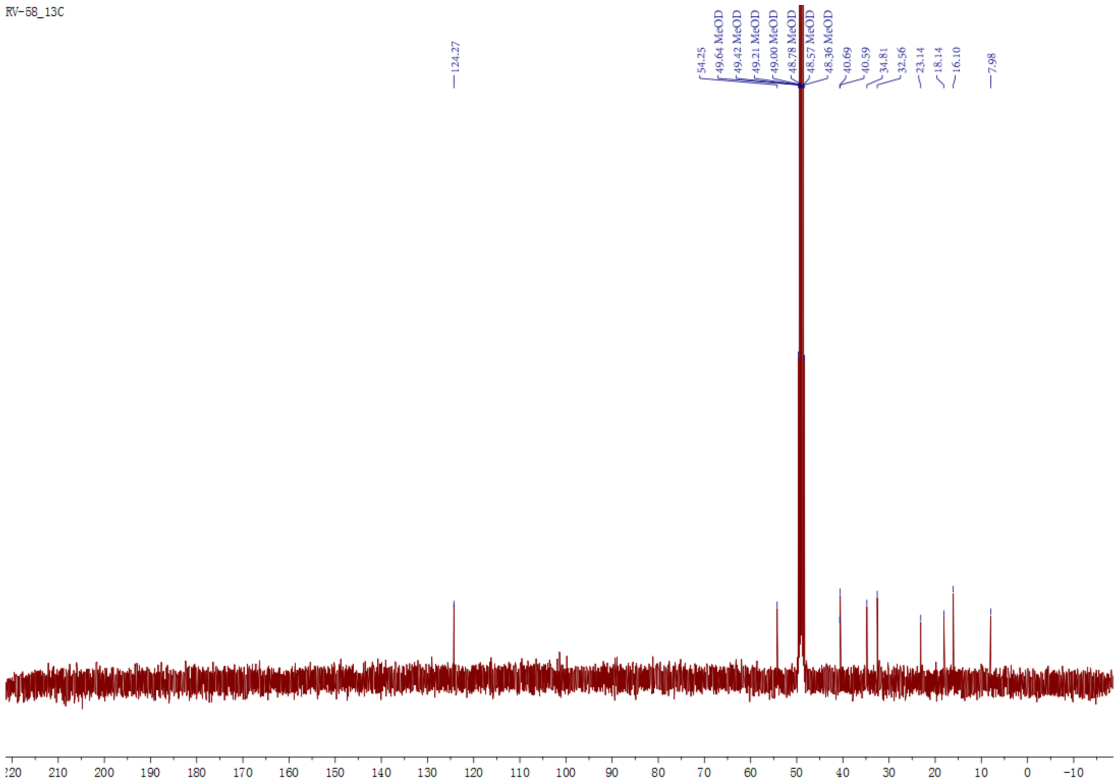

^{13}C NMR spectrum of **9**

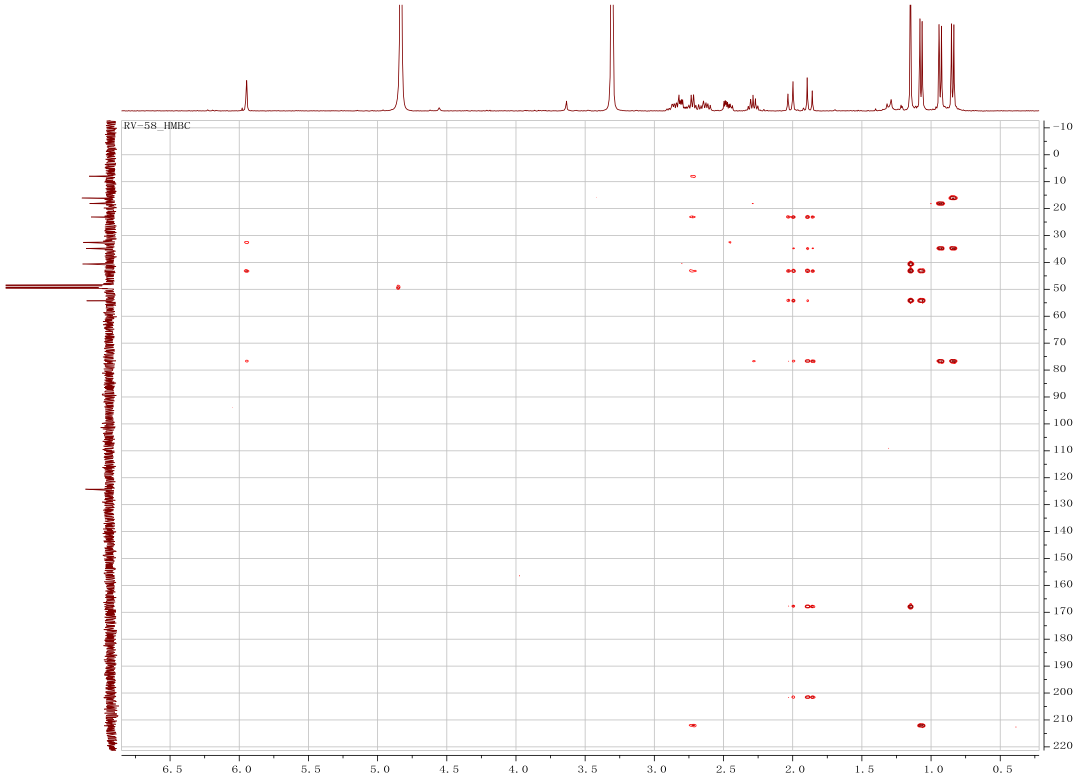

HMBC spectrum of **9**

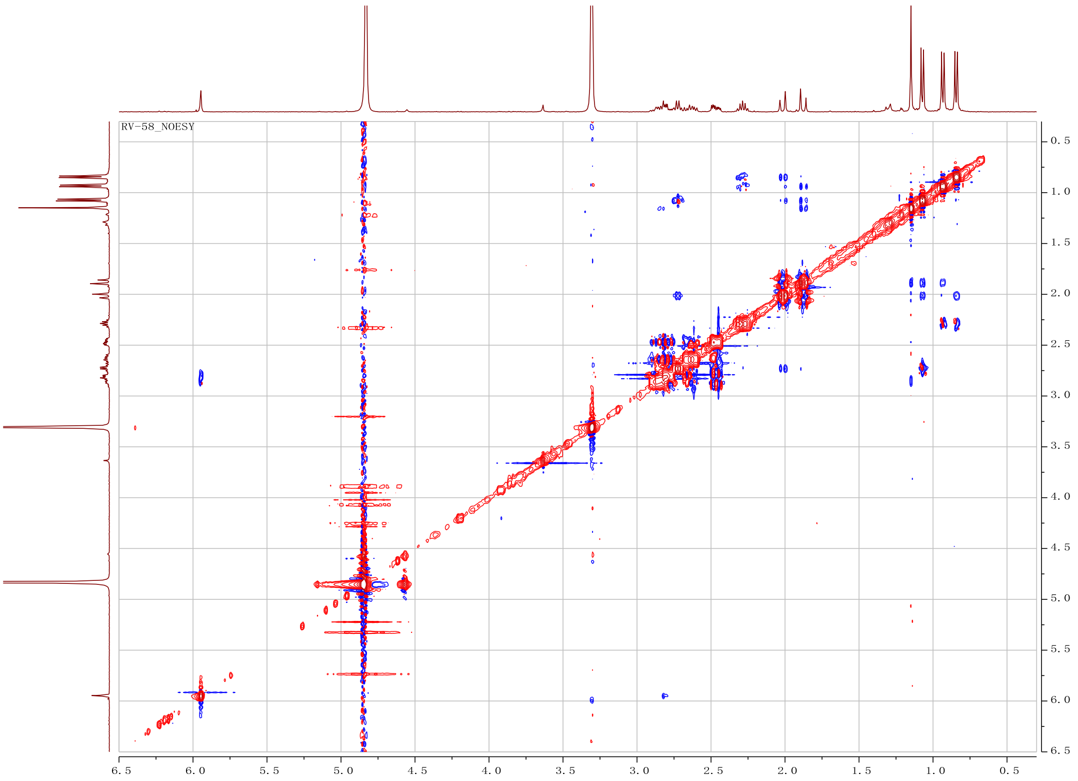

NOESY spectrum of **9**

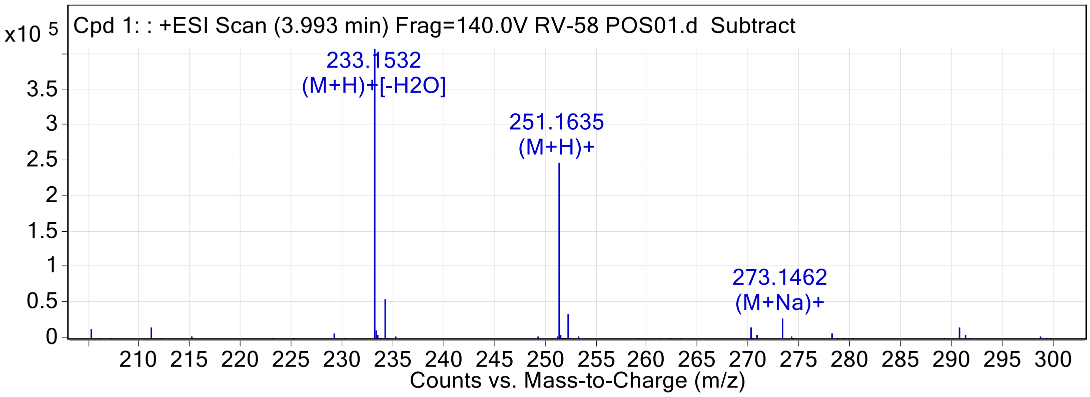

m/z	*Calc m/z*	Diff(ppm)	z	Abund	Formula	Ion
233.1532	233.1536	-1.89	1	408255.1	$C_{15}H_{21}O_2$	$(M+H)^+[-H_2O]$
251.1635	251.1642	-2.65	1	247640.1	$C_{15}H_{23}O_3$	$(M+H)^+$
273.1462	273.1461	0.25	1	28448.9	$C_{15}H_{22}NaO_3$	$(M+Na)^+$

HRESIMS spectrum of **9**

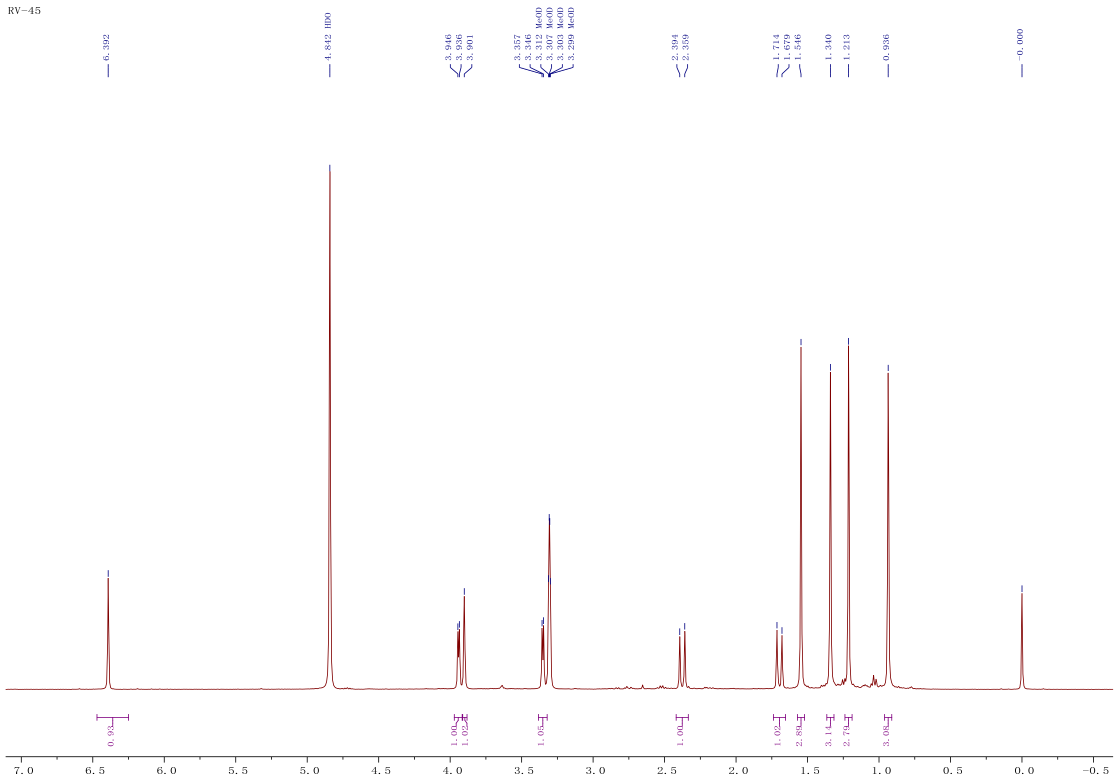

^{1}H NMR spectrum of **10**

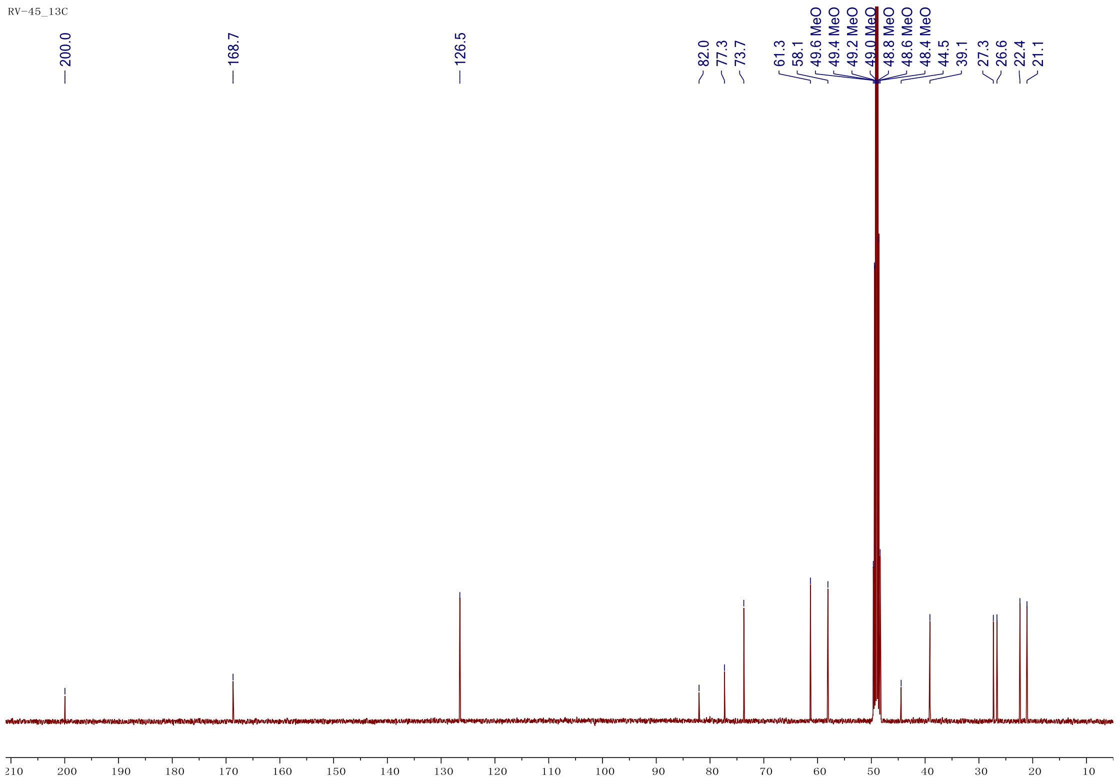

^{13}C NMR spectrum of **10**

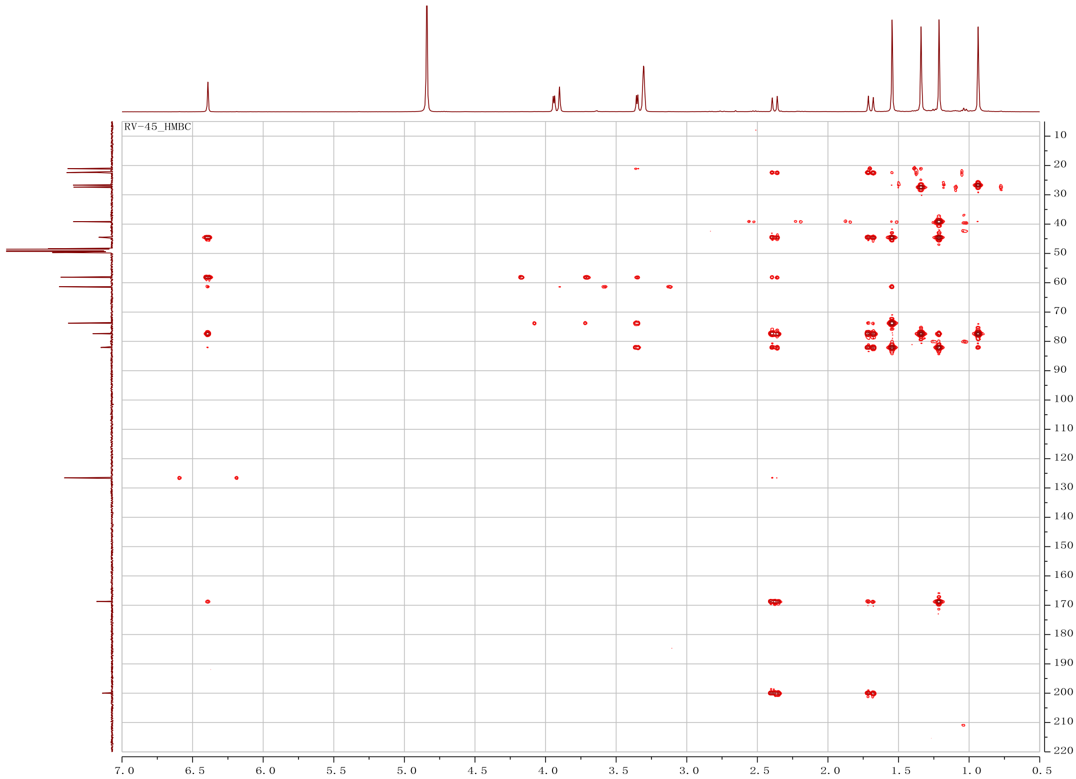

HMBC spectrum of **10**

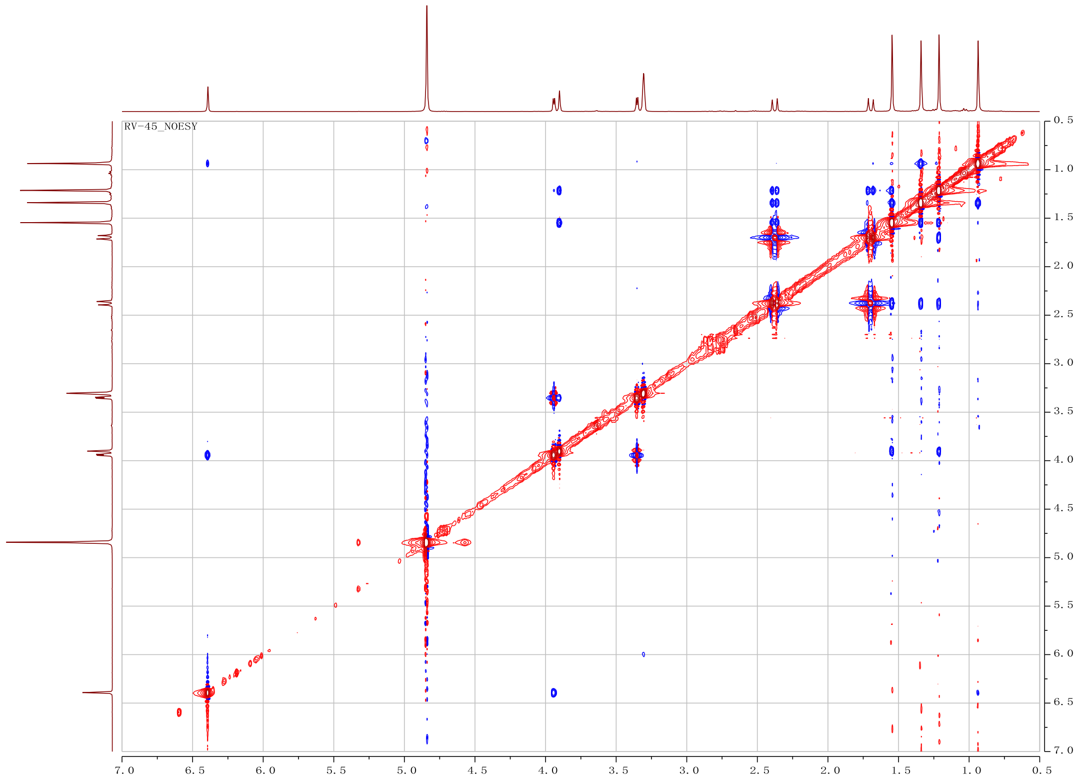

NOESY spectrum of **10**

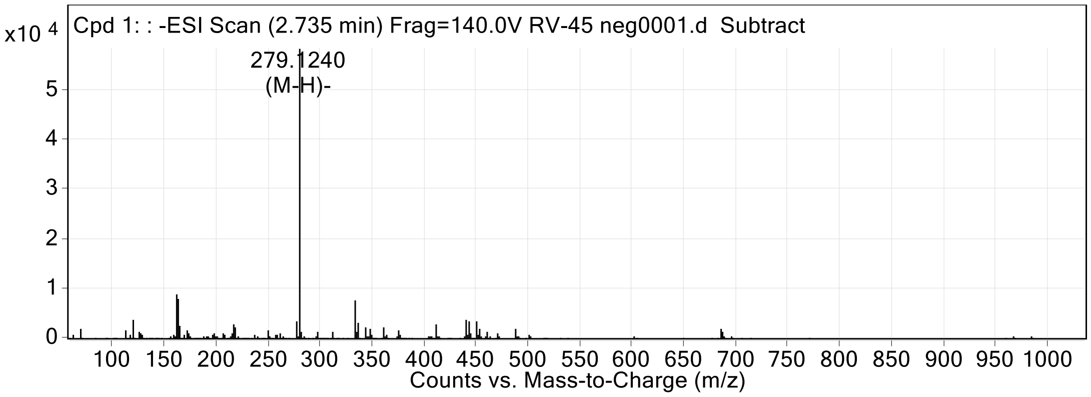

m/z	*Calc m/z*	Diff(ppm)	z	Abund	Formula	Ion
279.124	279.1238	0.72	-1	58422	$C_{15}H_{19}O_5$	$(M-H)^-$

HRESIMS spectrum of **10**

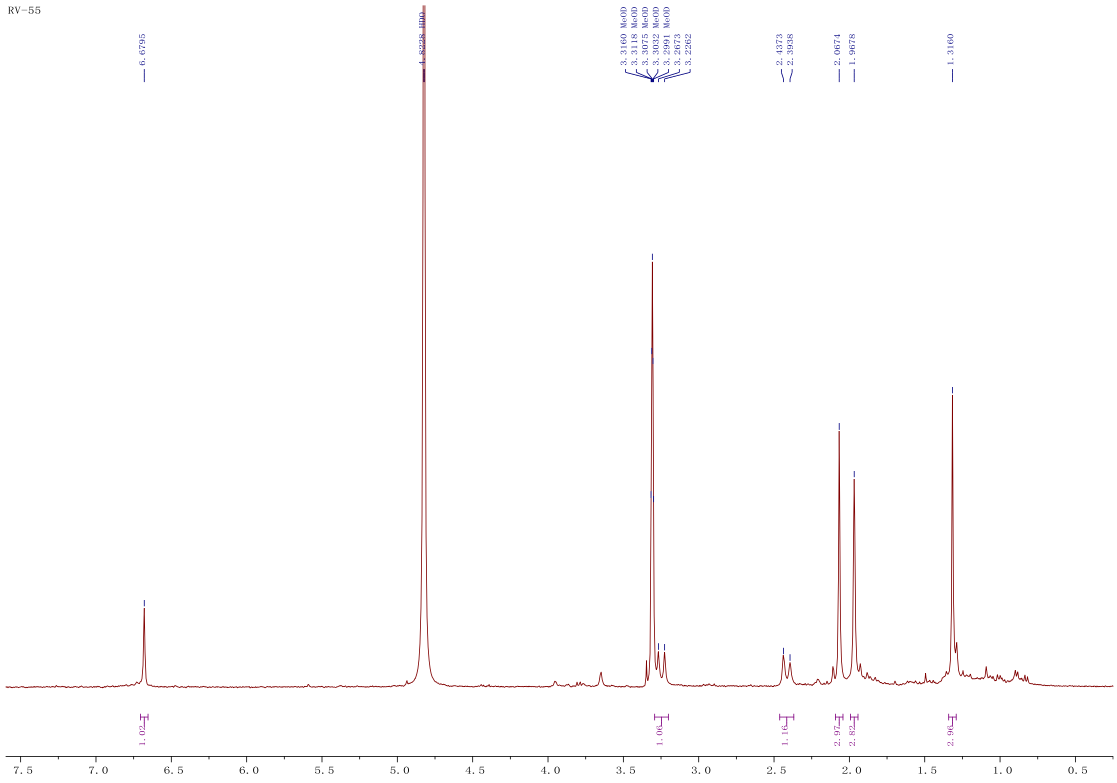

^{1}H NMR spectrum of **11**

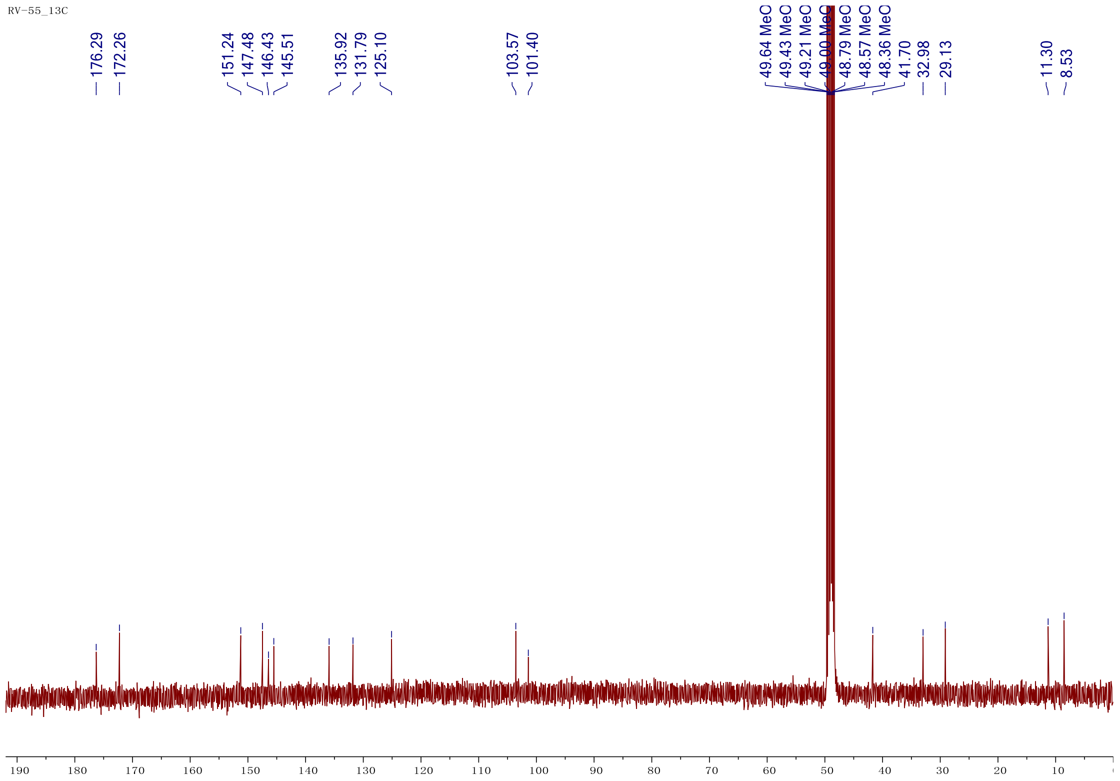

^{13}C NMR spectrum of **11**

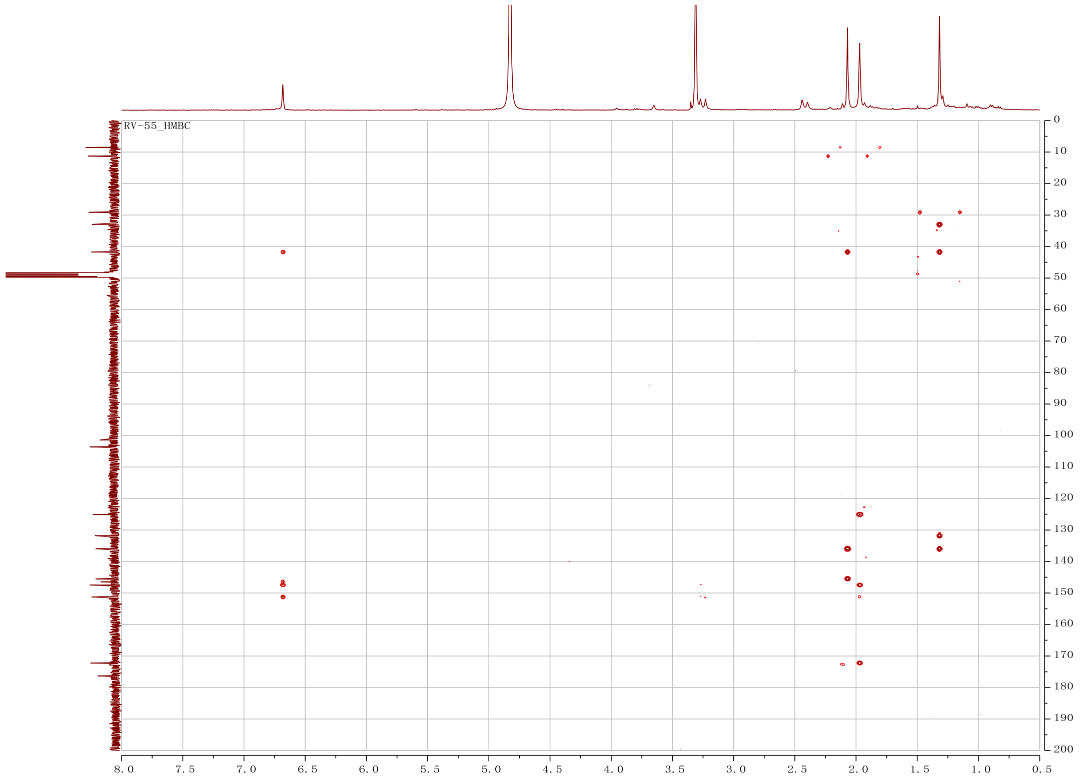

HMBC spectrum of **11**

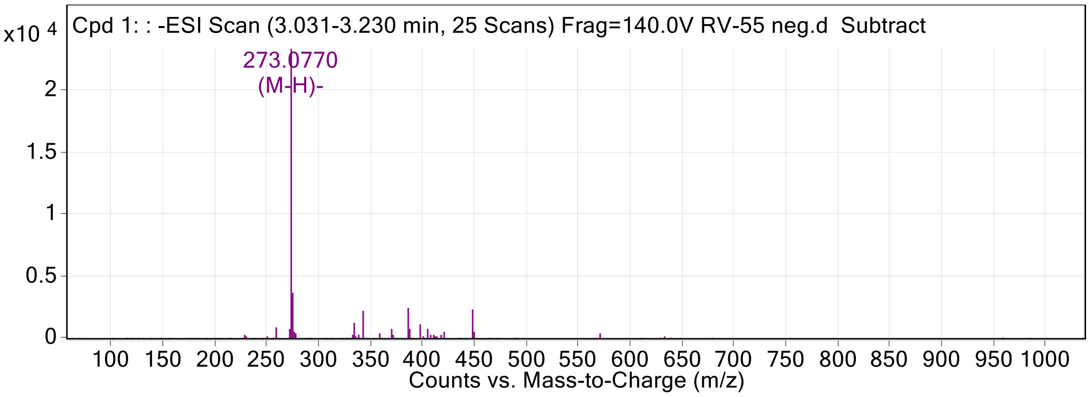

m/z	*Calc m/z*	Diff(ppm)	z	Abund	Formula	Ion
273.0770	273.0768	0.43	-1	23393	$C_{15}H_{13}O_5$	$(M-H)^-$

HRESIMS spectrum of **11**

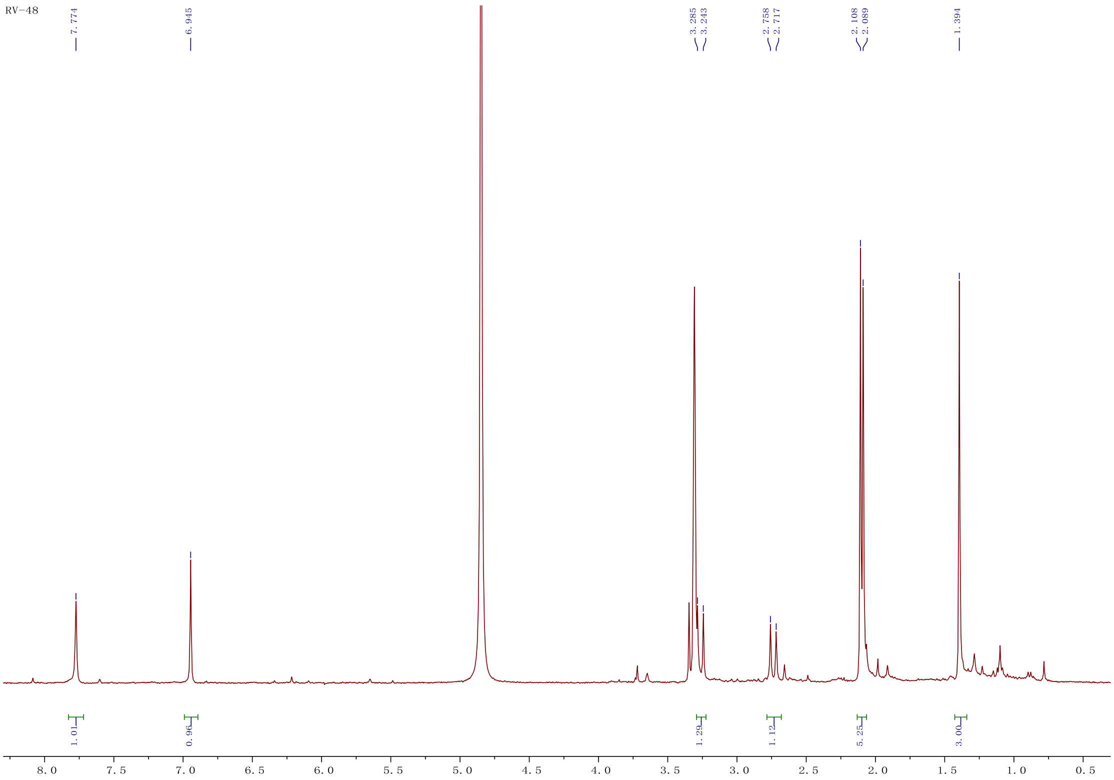

^{1}H NMR spectrum of **12**

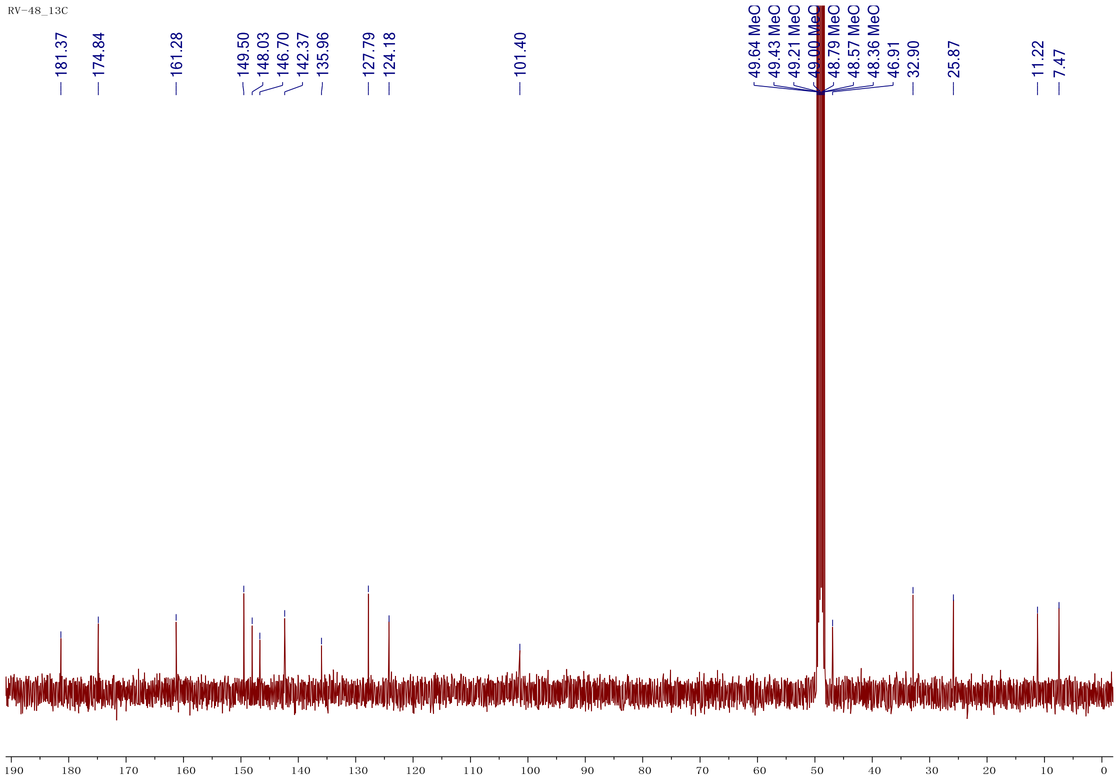

^{13}C NMR spectrum of **12**

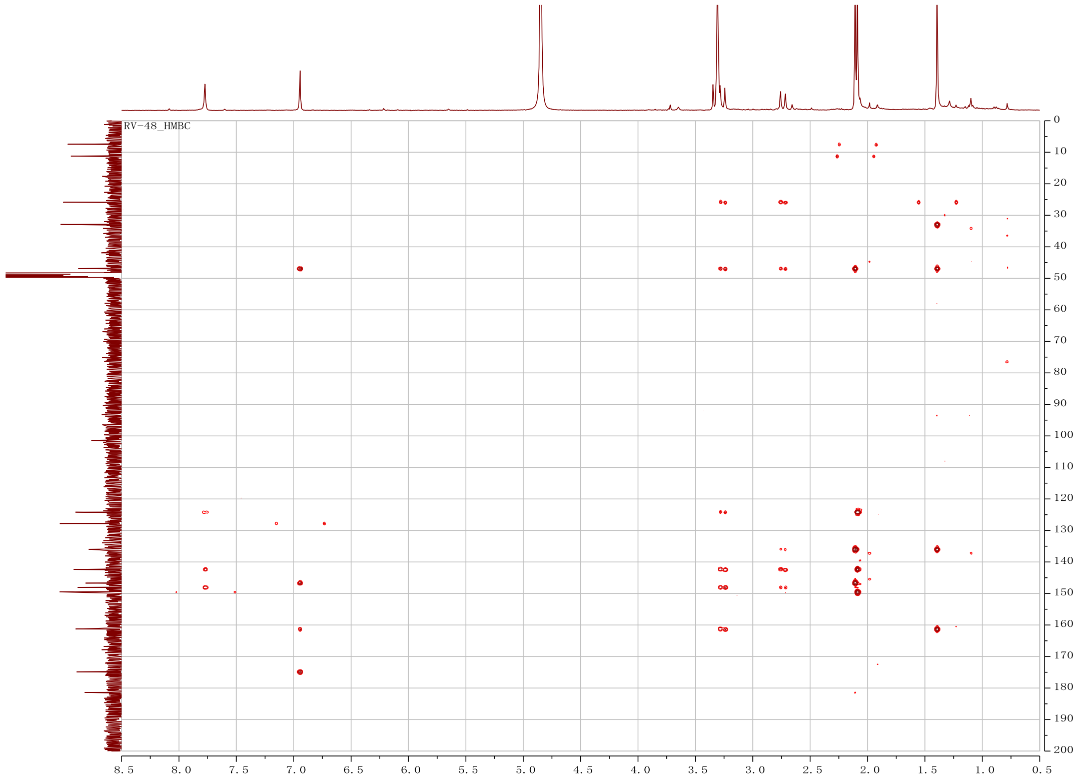

HMBC spectrum of **12**

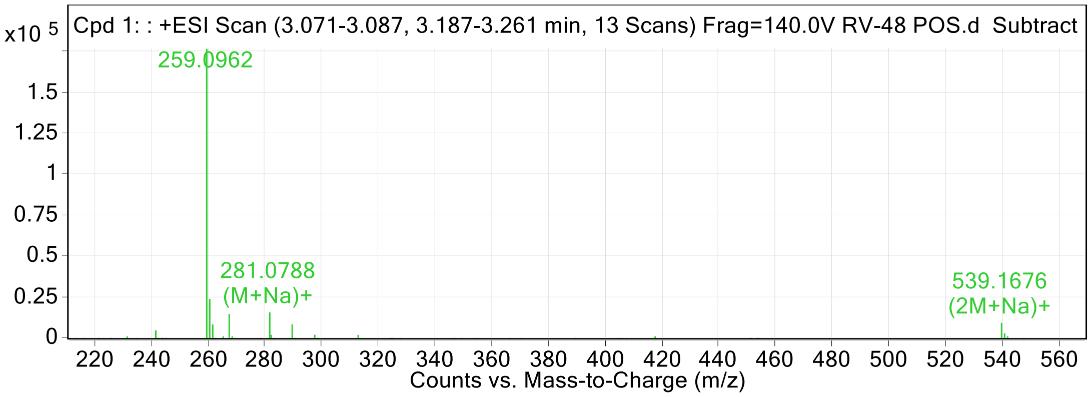

m/z	*Calc m/z*	Diff(ppm)	z	Abund	Formula	Ion
259.0962	259.0965	-1.21	1	176911.6	$C_{15}H_{15}O_4$	$(M+H)^+$
281.0788	281.0784	1.41	1	16374.6	$C_{15}H_{14}NaO_4$	$(M+Na)^+$
539.1676	539.1676	-0.13	1	10018.4	$C_{30}H_{28}NaO_8$	$(2M+Na)^+$

HRESIMS spectrum of **12**

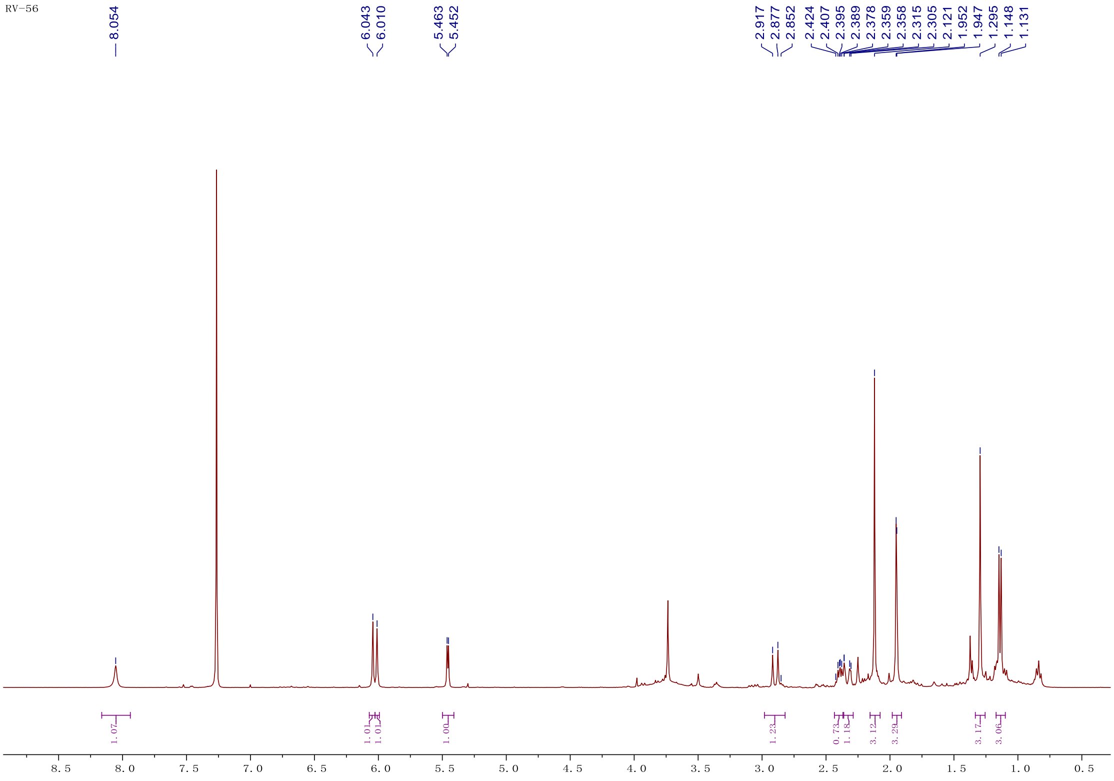

^{1}H NMR spectrum of **13** ($CDCl_3$, 400 MHz)

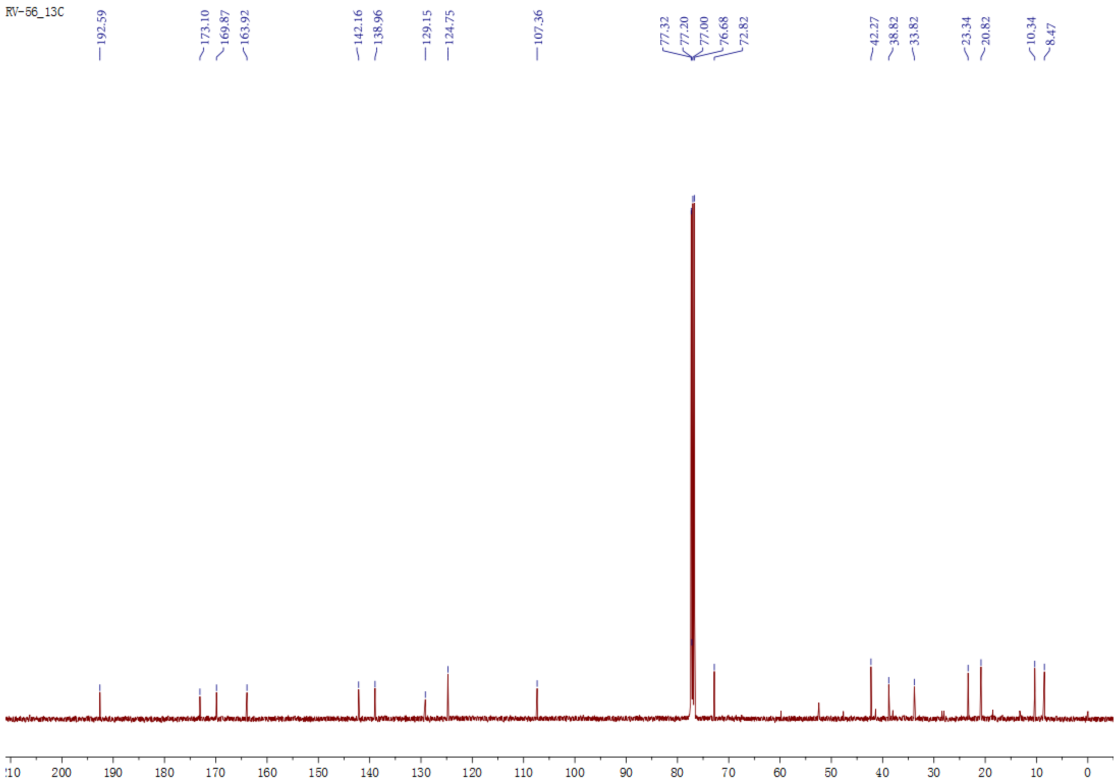

^{13}C NMR spectrum of **13** ($CDCl_3$, 100 MHz)

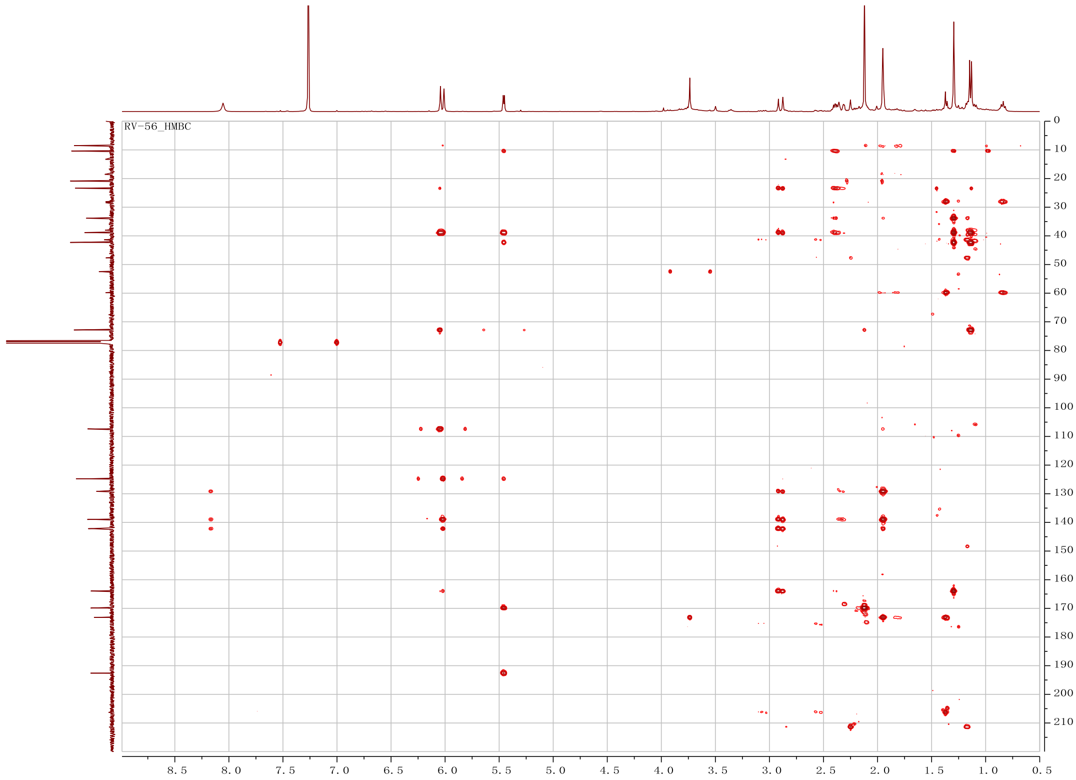

HMBC spectrum of **13**

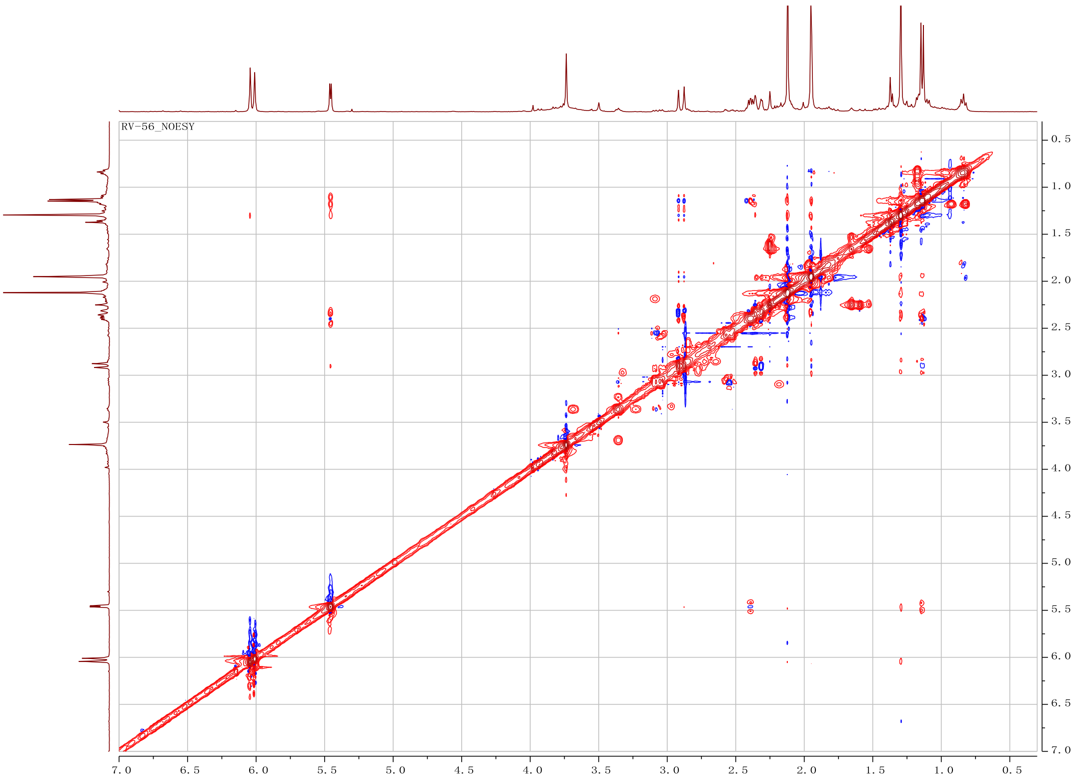

NOESY spectrum of **13**

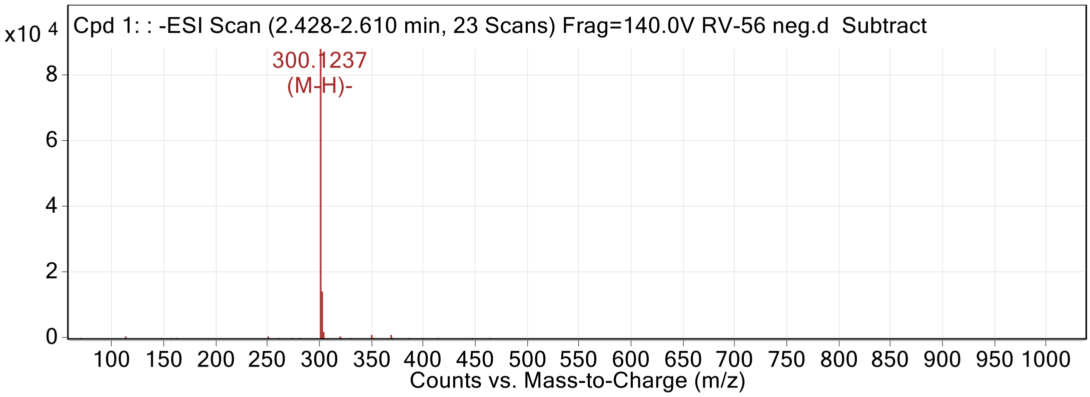

m/z	*Calc m/z*	Diff(ppm)	z	Abund	Formula	Ion
300.1237	300.1241	-1.34	-1	88045	$C_{17}H_{18}NO_4$	$(M-H)^-$

HRESIMS spectrum of **13**

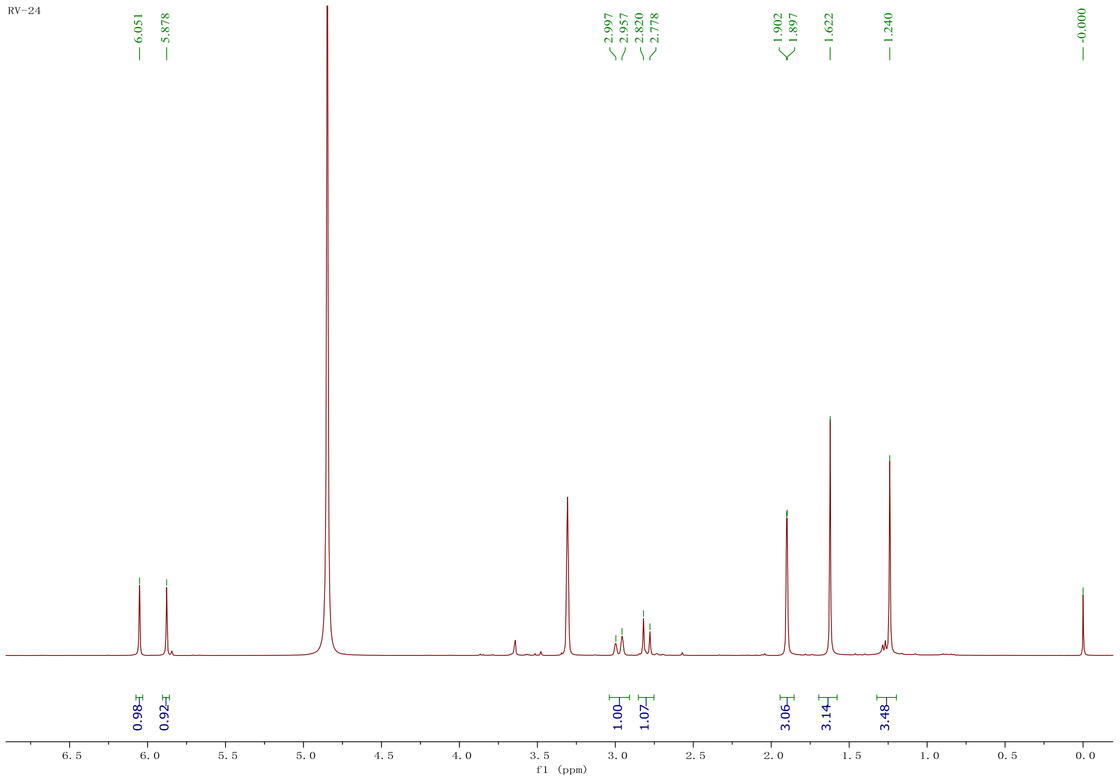

^{1}H NMR spectrum of **14** (CD_3OD, 400 MHz)

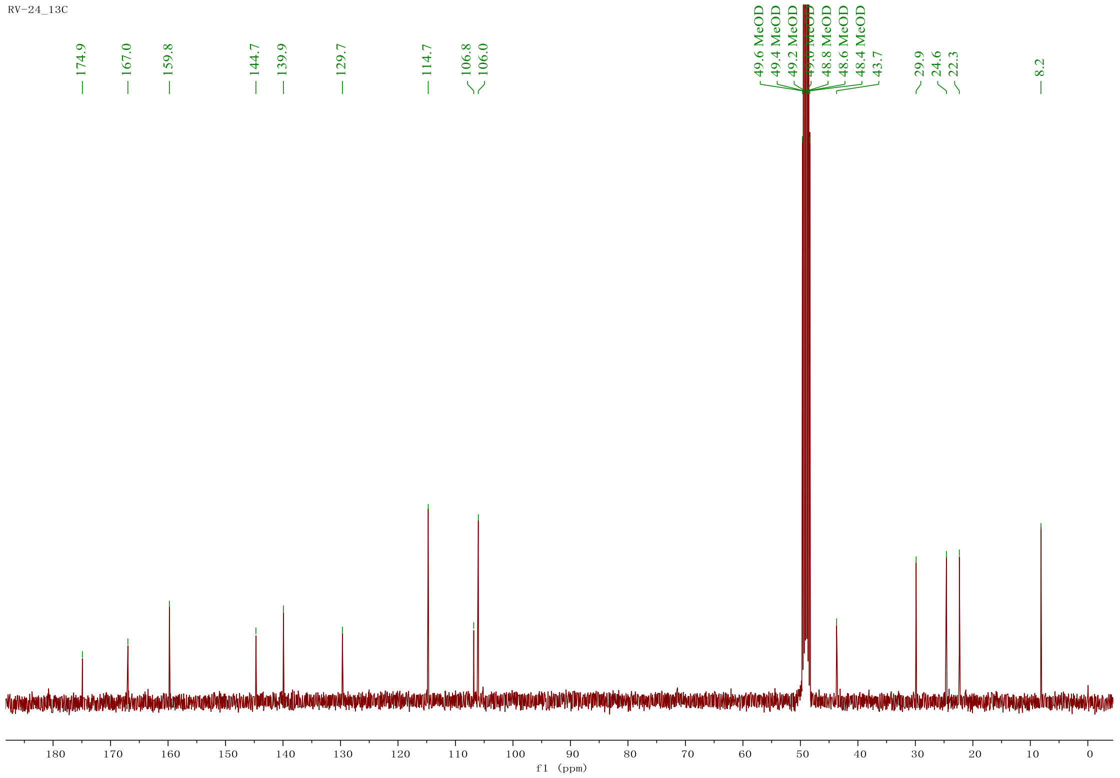

^{13}C NMR spectrum of **14** (CD_3OD, 100 MHz)

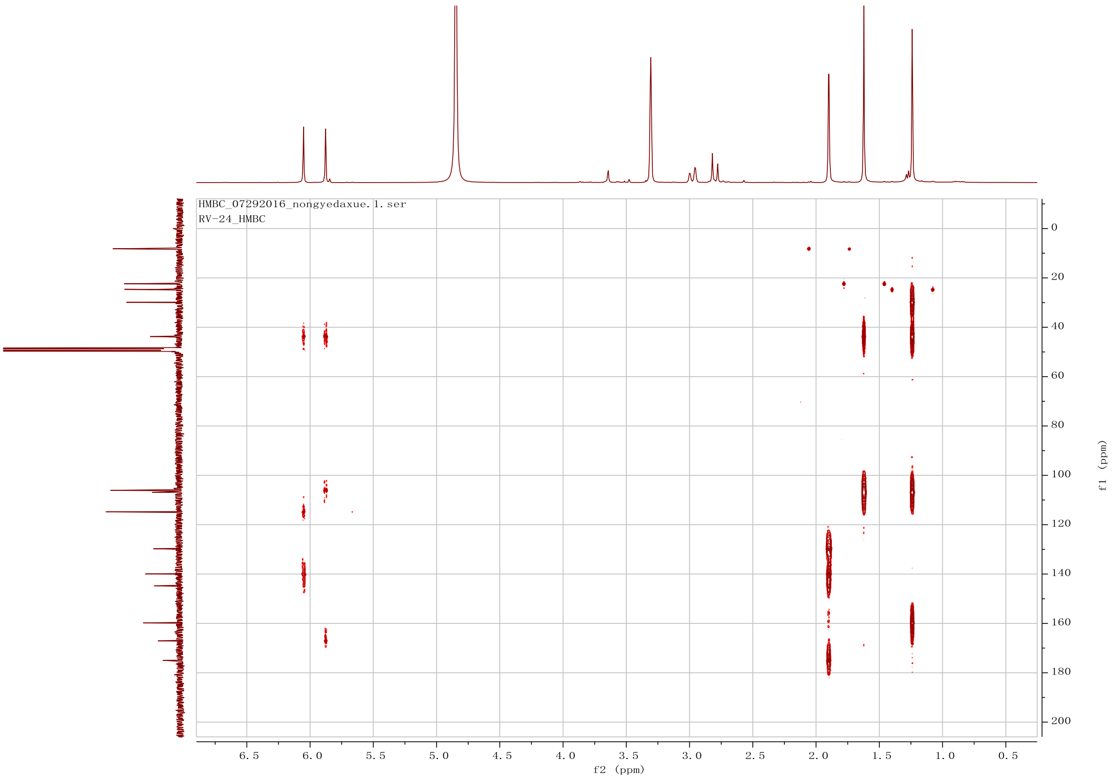

HMBC spectrum of **14** (CD_3OD)

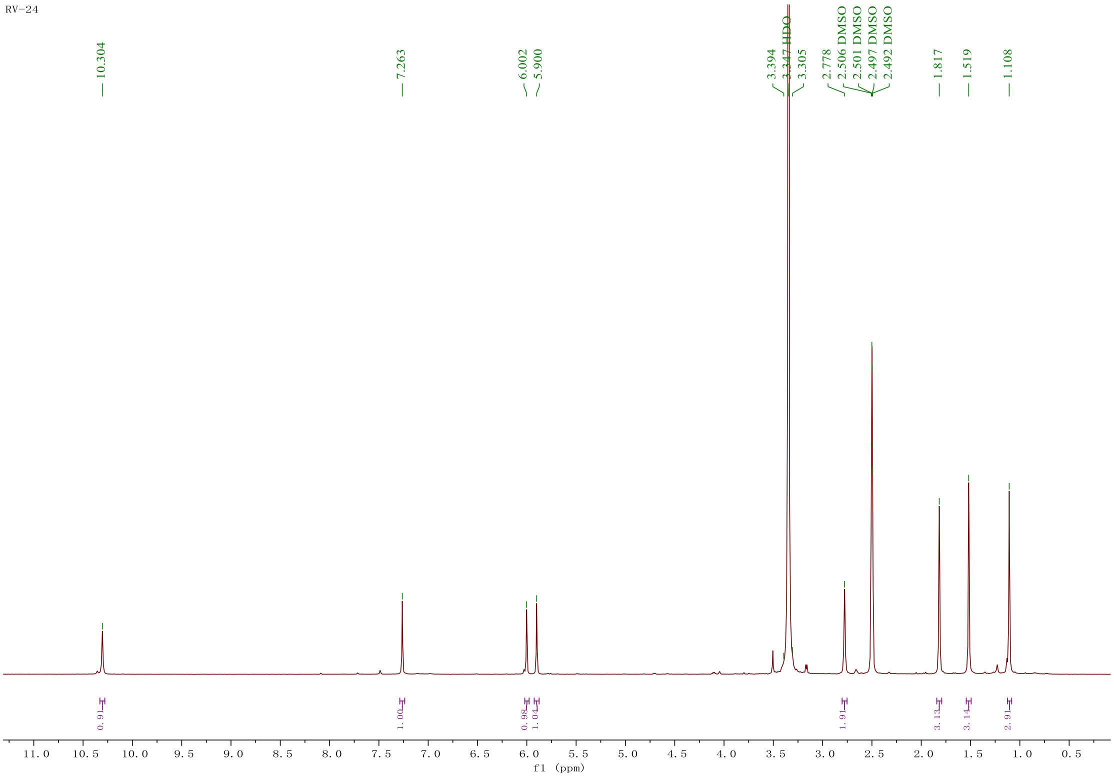

^{1}H NMR spectrum of **14** (DMSO-d_6, 400 MHz)

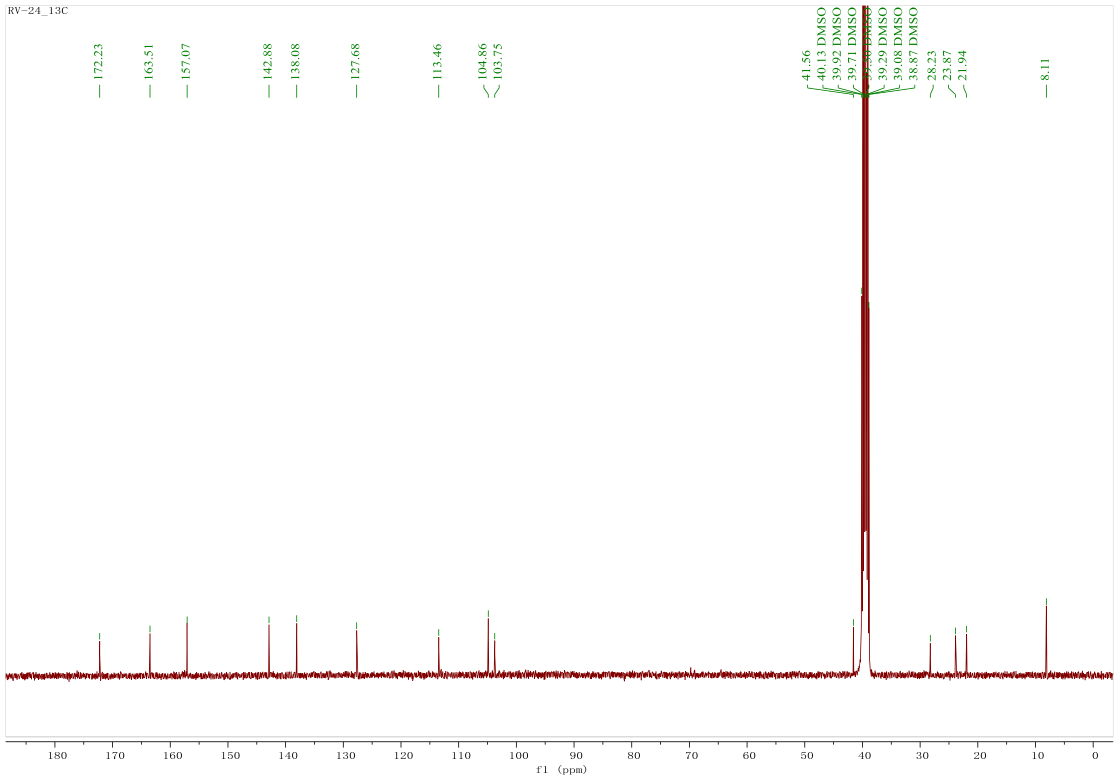

^{13}C NMR spectrum of **14** (DMSO-d_6, 100 MHz)

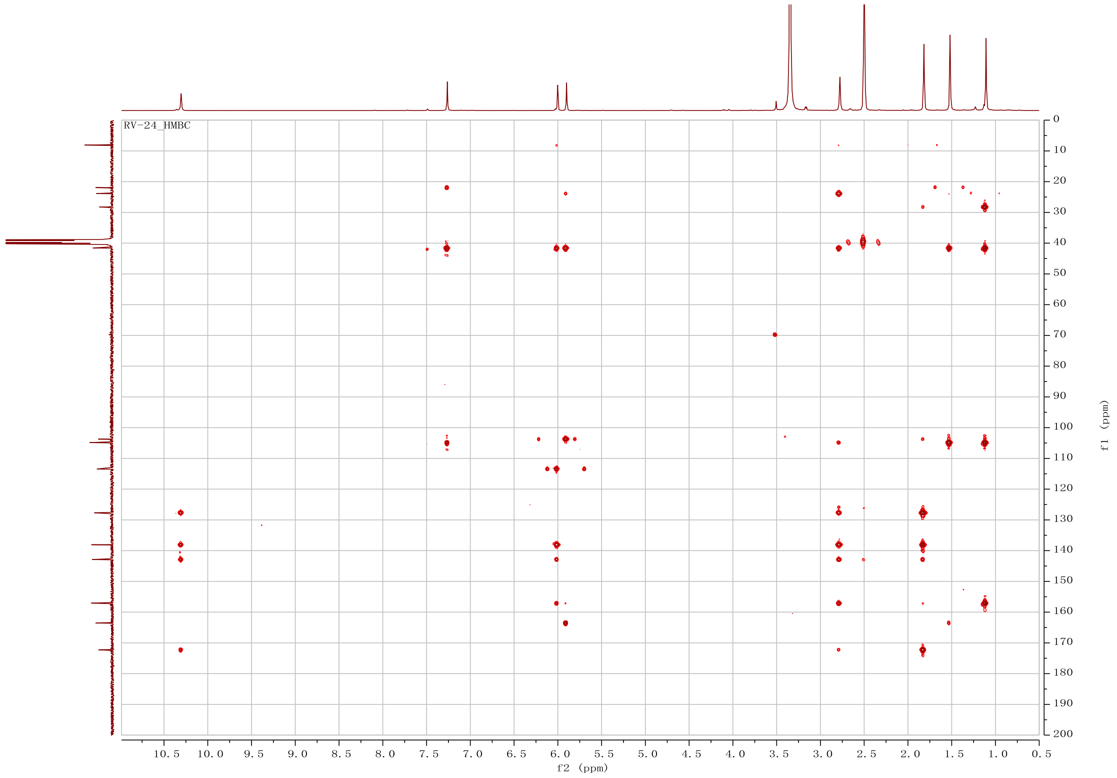

HMBC spectrum of **14** (DMSO-d_6)

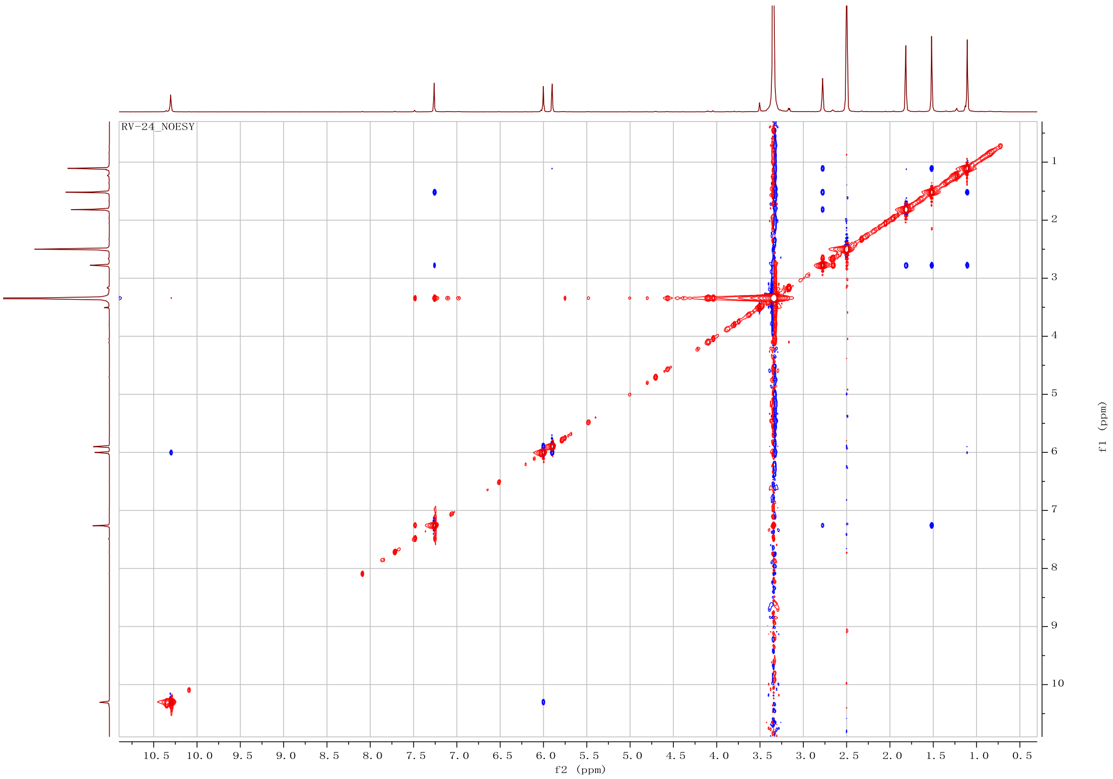

NOESY spectrum of **14** (DMSO-d_6)

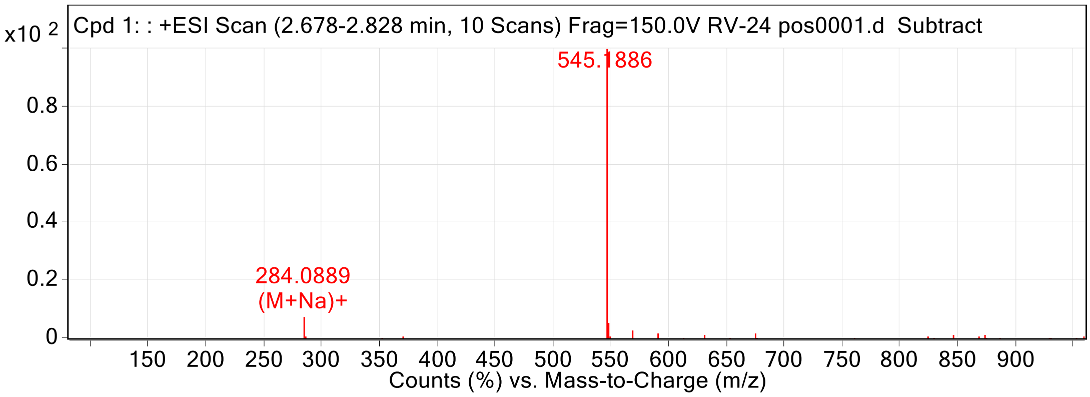

m/z	*Calc m/z*	Diff(ppm)	z	Abund	Formula	Ion
284.0889	284.0893	-1.55	1	2326.7	$C_{14}H_{15}NNaO_4$	$(M+Na)^+$
545.1886	545.1894	-1.51	1	30926	$C_{28}H_{30}N_2NaO_8$	$(2M+Na)^+$

HRESIMS spectrum of **14**
